# Inhibition of Pyrimidine Biosynthesis Pathway Suppresses Viral Growth through Innate Immunity

**DOI:** 10.1371/journal.ppat.1003678

**Published:** 2013-10-03

**Authors:** Marianne Lucas-Hourani, Daniel Dauzonne, Pierre Jorda, Gaëlle Cousin, Alexandru Lupan, Olivier Helynck, Grégory Caignard, Geneviève Janvier, Gwénaëlle André-Leroux, Samira Khiar, Nicolas Escriou, Philippe Desprès, Yves Jacob, Hélène Munier-Lehmann, Frédéric Tangy, Pierre-Olivier Vidalain

**Affiliations:** 1 Institut Pasteur, Unité de Génomique Virale et Vaccination, Paris, France; 2 CNRS, UMR3569, Paris, France; 3 Institut Curie, Centre de Recherche, Paris, France; 4 CNRS, UMR176, Paris, France; 5 Institut Pasteur, Unité de Chimie et Biocatalyse, Paris, France; 6 CNRS, UMR3523, Paris, France; 7 Institut Pasteur, Unité de Biochimie Structurale, Paris, France; 8 CNRS, UMR 3528, Paris, France; 9 Institut Pasteur, Unité Interactions moléculaires Flavivirus-Hôtes, Paris, France; 10 Institut Pasteur, Unité de Génétique Moléculaire des Virus à ARN, Paris, France; 11 Dana-Farber Cancer Institute, Center for Cancer Systems Biology (CCSB) and Department of Cancer Biology, Boston, Massachusetts, United States of America; Cleveland Clinic, United States of America

## Abstract

Searching for stimulators of the innate antiviral response is an appealing approach to develop novel therapeutics against viral infections. Here, we established a cell-based reporter assay to identify compounds stimulating expression of interferon-inducible antiviral genes. DD264 was selected out of 41,353 compounds for both its immuno-stimulatory and antiviral properties. While searching for its mode of action, we identified DD264 as an inhibitor of pyrimidine biosynthesis pathway. This metabolic pathway was recently identified as a prime target of broad-spectrum antiviral molecules, but our data unraveled a yet unsuspected link with innate immunity. Indeed, we showed that DD264 or brequinar, a well-known inhibitor of pyrimidine biosynthesis pathway, both enhanced the expression of antiviral genes in human cells. Furthermore, antiviral activity of DD264 or brequinar was found strictly dependent on cellular gene transcription, nuclear export machinery, and required IRF1 transcription factor. In conclusion, the antiviral property of pyrimidine biosynthesis inhibitors is not a direct consequence of pyrimidine deprivation on the virus machinery, but rather involves the induction of cellular immune response.

## Author Summary

Our therapeutic arsenal to treat viral diseases is extremely limited, and there is a critical need for molecules that could be used against multiple viruses. Among possible strategies, there is a growing interest for molecules stimulating cellular defense mechanisms. We recently developed a functional assay to identify stimulators of antiviral genes, and selected compound DD264 from a chemical library using this approach. While searching for its mode of action, we identified this molecule as an inhibitor of pyrimidine biosynthesis, a metabolic pathway that fuels the cell with pyrimidine nucleobases for both DNA and RNA synthesis. Interestingly, it was recently shown that inhibitors of this metabolic pathway prevent the replication of RNA viruses. Here, we established a functional link between pyrimidine biosynthesis pathway and the induction of antiviral genes, and demonstrated that pyrimidine biosynthesis inhibitors like DD264 or brequinar critically rely on cellular immune response to inhibit virus growth. Thus, pyrimidine deprivation is not directly responsible for the antiviral activity of pyrimidine biosynthesis inhibitors, which rather involves the induction of a metabolic stress and subsequent triggering of cellular defense mechanisms.

## Introduction

Infections by RNA viruses represent a major burden for public health. It includes major human pathogens such as influenza virus, measles virus, dengue virus or respiratory syncytial virus that are responsible for hundreds of thousands of human death every year. Although efficient prophylactic treatments, and in particular vaccines, can be used to protect individuals from some of these pathogens, our therapeutic arsenal is extremely limited [1]. Clinically used treatments are essentially based on ribavirin or recombinant type I interferons (IFN-α/β) that are of highly variable efficacy depending on targeted virus [1], [2]. Inhibitors of influenza virus or human respiratory syncytial virus have been developed, but such virus-specific treatments are useless against other RNA viruses [1]. Furthermore, RNA viruses are extremely diverse in terms of replication machinery, and this greatly complicates the design of broad-spectrum antiviral molecules. They also tend to escape drugs that target viral proteins through mutations, thus calling for innovative therapeutic approaches. Among possible strategies, chemical modulators of host pathways [3], [4], [5], [6], and in particular stimulators of innate immune response that boost cellular defenses to eliminate viral pathogens are of growing interest [7], [8], [9], [10]. In principle, such molecules would be efficient against a large panel of viral pathogens since the host immune response relies on a multiplicity of antiviral effectors that block viruses at several steps of their replication, and cover the variety of replication strategies they use.

The innate immune response is initiated by the recognition of Pathogen-Associated Molecular Patterns (PAMPs) by different classes of Pattern Recognition Receptors (PRRs). Along their replication cycle, RNA viruses produce several well-characterized PAMPs, such as double-stranded RNA, uncapped 5′-triphosphate RNA or single-stranded RNA molecules [11], [12]. PRRs that recognize such virus-associated molecular motifs essentially belong to two protein families: toll-like receptors (TLRs) and RIG-I like receptors (RLRs). TLRs are transmembrane receptors, and only three members of this family have been reported to detect RNA molecules with their extracellular domain: TLR3 that binds double-stranded RNA and TLR7/8 that are activated by G/U rich single-stranded RNA [13]. RIG-I and IFIH1/MDA5 are best-characterized members of the RLR family. These cytosolic sensors are expressed by virtually all cell types to detect short 5′-triphosphate and long double-stranded RNA molecules, respectively [14]. Upon activation by their ligands, TLRs and RLRs initiate signaling cascades that converge on three families of transcription factors (NF-κB, IRF3/7, and ATF-2/Jun) to induce genes encoding antiviral effectors and type I IFN (IFN-α/β) secretion. Secreted IFN-α/β subsequently amplify the antiviral response through binding to their membrane receptor at the surface of both infected cells and neighboring cells [11], [15]. This activates a Jak/STAT signaling cascade that further stimulates the expression of antiviral genes in the infected organ. Human genome contains hundreds of IFN-inducible genes, and a large fraction encode for restriction factors to target viruses at multiple steps of their replication cycle [16], [17].

To identify chemical compounds stimulating this pathway, several strategies have been developed. Molecules that engage TLRs or IFN-α/β receptors have been identified using various combinations of functional screens, *in silico* molecular docking and binding assays [18], [19]. Phenotypic screens have also been used to identify stimulators of the antiviral gene cluster [9], [20], [21], [22], [23], [24], [25]. Several groups have recently described similar assays based on cells transfected with a reporter gene under control of IFN-stimulated response elements (ISRE) [21], [22], [23]. This led to the identification of small molecules showing some antiviral activity *in vitro*. In the current report, we used a similar strategy to screen a compound collection of 41,353 molecules, and identified compound DD264 as a molecule stimulating the expression of antiviral genes in treated cells and exhibiting a potent antiviral activity *in vitro*. While searching for its mode of action, we found that compound DD264 targets the *de novo* pyrimidine biosynthesis pathway. This allowed us to establish for the first time a link between inhibition of pyrimidine biosynthesis, amplification of antiviral gene expression, and inhibition of RNA virus infections.

## Results

### High-throughput screening for stimulators of ISRE-regulated genes

**Figure 1:**
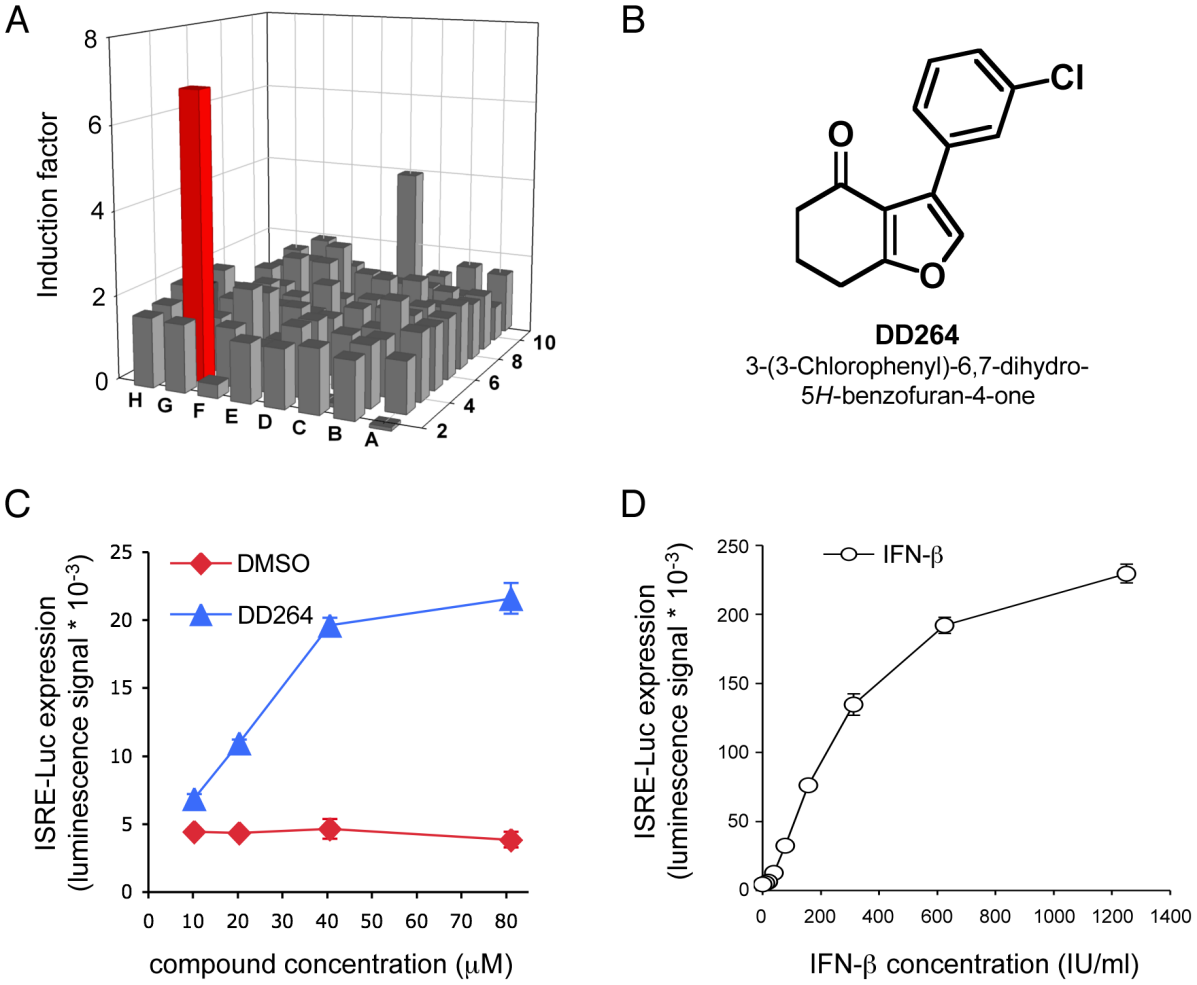
DD264 activates the ISRE-luciferase reporter gene. (**A**) Bar chart showing results for one 96-well screening plate. ISRE-luciferase reporter gene induction is expressed as a fold change compared to control wells treated with DMSO alone. Each bar represents one compound and the red bar corresponds to DD264 (induction factor = 6.9). Compounds from this representative plate were from Institut Curie library and were screened at 20 µg/ml, thus corresponding to a final concentration of 81.1 µM for DD264. (**B**) Chemical structure of DD264. (**C**) HEK-293 cells that express luciferase under control of five interferon-stimulated response elements (ISRE) were incubated with increasing doses of DD264 or DMSO alone. After 24 hours, luciferase expression was determined. (**D**) Same experiment as in (C), but cells were stimulated with increasing doses of recombinant IFN-β. Except for (A) that corresponds to one representative experiment, all experiments were performed in triplicate, and data represent means ± SD.

To identify chemical compounds that stimulate expression of IFN-inducible genes, we have developed a high-throughput screening assay based on human HEK-293T cells transiently transfected with a luciferase reporter gene under control of five IFN-stimulated response elements (ISRE). A total of 41,353 chemical compounds, with final concentrations ranging from 30 to 130 µM depending on the library, were screened with this assay for their capacity to stimulate ISRE-luciferase expression in human cells (see Materials and Methods and Figure S1 for details). For each screening plate, the amplification of luciferase signal in control wells was found >45 when recombinant IFN-β was added. Although most tested molecules were not inducers of reporter gene expression, five compounds showed a reproducible although modest >4-fold amplification of luciferase activity (data not shown). Three of them showed a strong toxicity in culture and were discarded. Two compounds from the chemical library of Institut Curie were finally selected for further studies including DD264 (Figure 1A and 1B) and DD363, which will be described elsewhere.

To further investigate DD264 biological activity, we established HEK-293 cells stably transfected with the ISRE-luciferase reporter gene. In agreement with screening data, DD264 induced some significant expression of the ISRE-luciferase reporter gene in this system (Figure 1C), but was much less efficient than recombinant IFN-β to stimulate the ISRE promoter (Figure 1D). Interestingly, ISRE stimulation by DD264 was independent of IFN-α, β, or γ induction as assessed by qRT-PCR and ELISA (Table S1 and S2). A cocktail of blocking antibodies against IFN-α/β also had no effect on ISRE-luciferase induction by DD264 (data not shown). Altogether, this demonstrated that a different pathway is involved.

Visual examination of treated cultures suggested that DD264 inhibited cellular proliferation. This was confirmed by quantifying over several days the number of living cells in cultures treated or not with increasing amounts of DD264 (Figure S2A). Cells proliferated in control wells and did not when treated with DD264, but the number of living cells remained stable (did not collapse). Inhibition of cellular proliferation was also shown by propidium iodide labeling and flow cytometry analysis of cellular DNA content (Figure S2B). We finally determined that DD264 did not induce cell death even at the highest concentration tested (80 µM) as assessed by quantifying DNA fragmentation (Figure S2B).

### DD264 has a broad-spectrum antiviral activity

**Figure 2:**
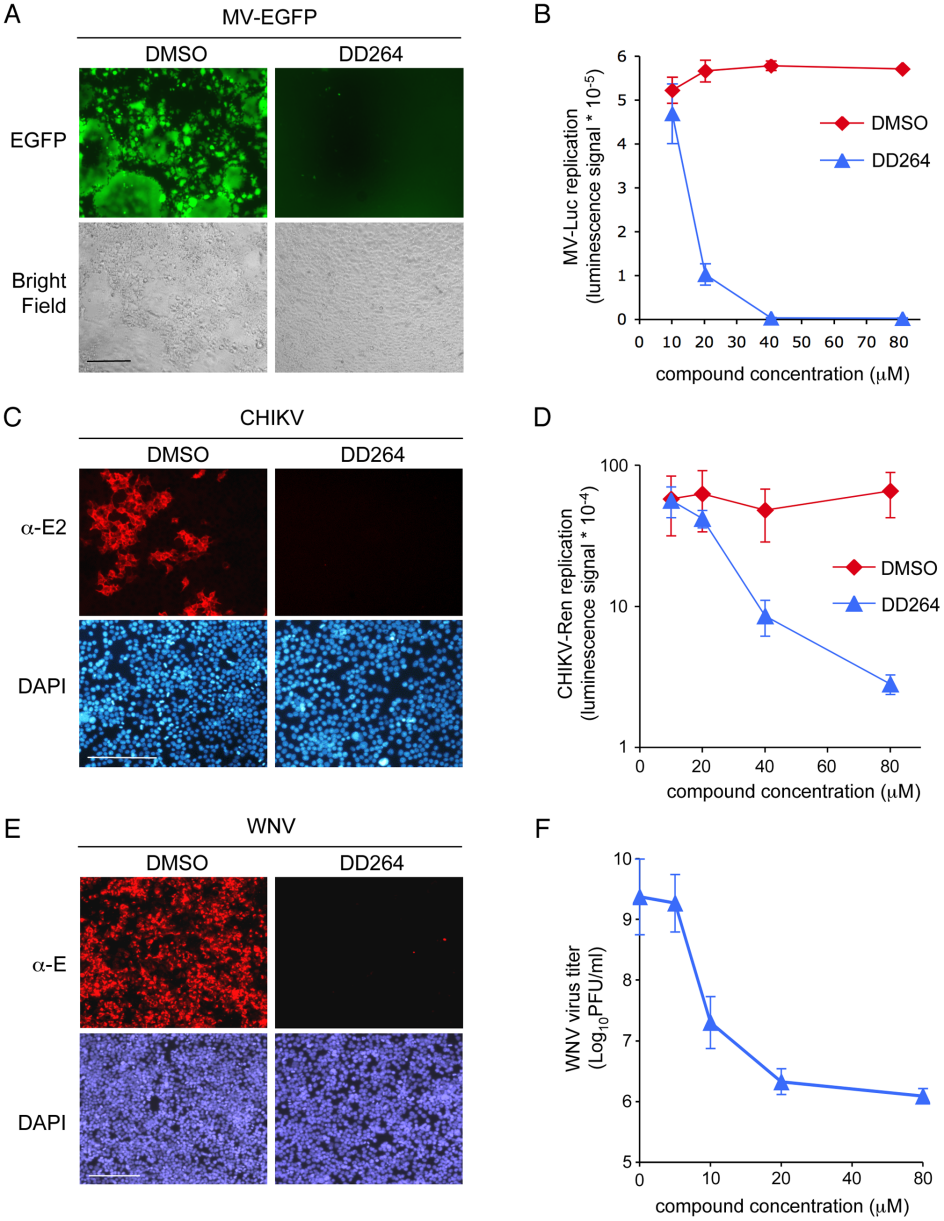
DD264 is a broad-spectrum antiviral molecule. (**A**) HEK-293T cells were infected with a recombinant strain of MV expressing EGFP (MOI = 0.1), and incubated for 48 hours in the presence of DD264 at 40 µM or DMSO alone. Scale bar = 200 µm. (**B**) HEK-293T cells were infected with a recombinant strain of MV expressing luciferase (MOI = 0.1), and incubated with increasing doses of DD264 or DMSO alone. After 24 hours, luciferase expression was determined. (**C**) HEK-293T cells were infected with CHIKV (MOI = 0.1), and incubated with DMSO alone or DD264 at 40 µM. After 24 hours, cells were fixed, and CHIKV E2 glycoprotein was detected by immunostaining. Cell nuclei were stained with DAPI. Scale bar = 200 µm. (**D**) HEK-293T cells were infected with a recombinant strain of CHIKV expressing *Renilla* luciferase (MOI = 0.2), and incubated with increasing doses of DD264 or DMSO alone. After 24 hours, *Renilla* luciferase expression was determined. Experiments in (B) and (D) were performed in triplicate, and data represent means ± SD. (**E**) HEK-293T cells were infected with WNV (MOI = 1), and incubated with DMSO alone or DD264 at 40 µM. After 24 hours, cells were fixed and WNV E glycoprotein was detected by immunostaining. Cell nuclei were stained with DAPI. Scale bar = 200 µm. (**F**) HEK-293T cells were infected with WNV (MOI = 10), washed, and incubated with increasing doses of DD264 or matching volumes of DMSO alone. After 24 hours, supernatants were recovered, clarified by centrifugation and titrated. Results are expressed as log_10_ PFU (plaque-forming units) per ml. Experiment was performed twice, and data represent means ± SD. (**A–F**) In all experiments, DD264 (or control DMSO) was added to cell cultures at the time or few minutes after infection.

DD264 was tested for its inhibitory effect on the replication of several human viruses of clinical importance and from different families. DD264 was first tested on measles virus (MV), a *Paramyxoviridae* that can be considered as a prototype of negative-strand RNA viruses. Human HEK-293T cells were infected with a recombinant MV strain expressing either EGFP (MV-EGFP) or luciferase (MV-Luc) from an additional transcription unit, and then treated with DD264. As shown in Figure 2A and 2B, DD264 suppressed MV replication as assessed by inhibition of EGFP or luciferase expression. As shown in Figure 2B, IC_50_ of DD264 was about 15 µM in this assay. DD264 also inhibited the production of infectious viral particles (Figure S3A). Finally, we also established that DD264 antiviral activity was not restricted to HEK-293T cells, and could be observed on other cell lines like HeLa and MRC5 cells (Figure S3B and S3C).

We then tested DD264 activity on the growth of a positive-strand RNA virus, chikungunya virus (CHIKV), an emerging mosquito-transmitted *Alphavirus* (*Togaviridae* family) responsible for arthralgia in human. HEK-293T cells were infected with wild-type CHIKV or a recombinant variant expressing *Renilla* luciferase as a reporter [26], [27], [28], and viral infection was determined by immunostaining with anti-E2 mAb or measuring luciferase activity. DD264 efficiently suppressed CHIKV growth in infected cell cultures (Figure 2C and 2D). Similar results were obtained on West Nile virus, an emerging mosquito-transmitted *Flavivirus* (*Flaviviridae* family) associated to acute encephalitis (Figure 2E). DD264 also inhibited the production of infectious WNV particles in a dose-dependent manner (Figure 2F). Antiviral activity of DD264 was also tested on a non-enveloped positive-strand RNA virus, coxsackievirus B3 (CVB3), which is a prototype of *Picornaviridae* family. Following DD264 treatment, CVB3 viral production in cell cultures was strongly impaired (Figure S3D). Therefore, DD264 has a potent antiviral activity against various unrelated RNA viruses in cell cultures.

### Structure/activity analysis of DD264 analogs

To identify chemical features of DD264 accounting for its antiviral activity, a broad set of about 70 structural analogs were synthesized from appropriate β-chloro-β-nitrostyrenes according to known procedures ([29], [30], [31], [32] and Protocol S1), and then tested for their antiviral activity on MV. All 2,3-dihydro-2-nitro derivatives (Table S3, formula A) were almost inactive. Regarding compounds with general formula B, a larger halogen (DD700, GAC25), a vinyl (GAC50) or an alkyne group (JP4, JP6) could replace the chlorine atom at position R^8^ of DD264. Other substituents led to a significant or total loss of antiviral activity. In addition, the meta position of the chlorine atom was found to be optimal, as compounds bearing a chloro substituent in ortho (DD706) or para (DD703) were much less active. Interestingly, the chlorine atom at position R^8^ could be replaced by a much larger group such as 3-chlorophenyl (JP13) or 3-bromophenyl (JP33) without any loss of activity. It was also possible to add a halogen at position R^1^ (*e.g.* a bromine atom) and this even increased the compound potency. Unfortunately, the synthesis of this bromo derivative (JP61f2) only proceeded in a poor yield, and further functional analyses were thus performed on DD264. Finally, we found that the carbonyl function can be moved from position 4 to position 7 of the tetrahydrobenzofuran skeleton without any significant loss of potency (DD829). All other tested structural modifications, substitutions or additions performed on the cyclohexanone nucleus induced a decrease or a complete loss of activity. Altogether, this established a clear structure/activity relationship for this series, and provides a chemical framework to further improve activity and pharmacological properties of DD264.

### DD264 amplifies cellular innate immune response

**Figure 3:**
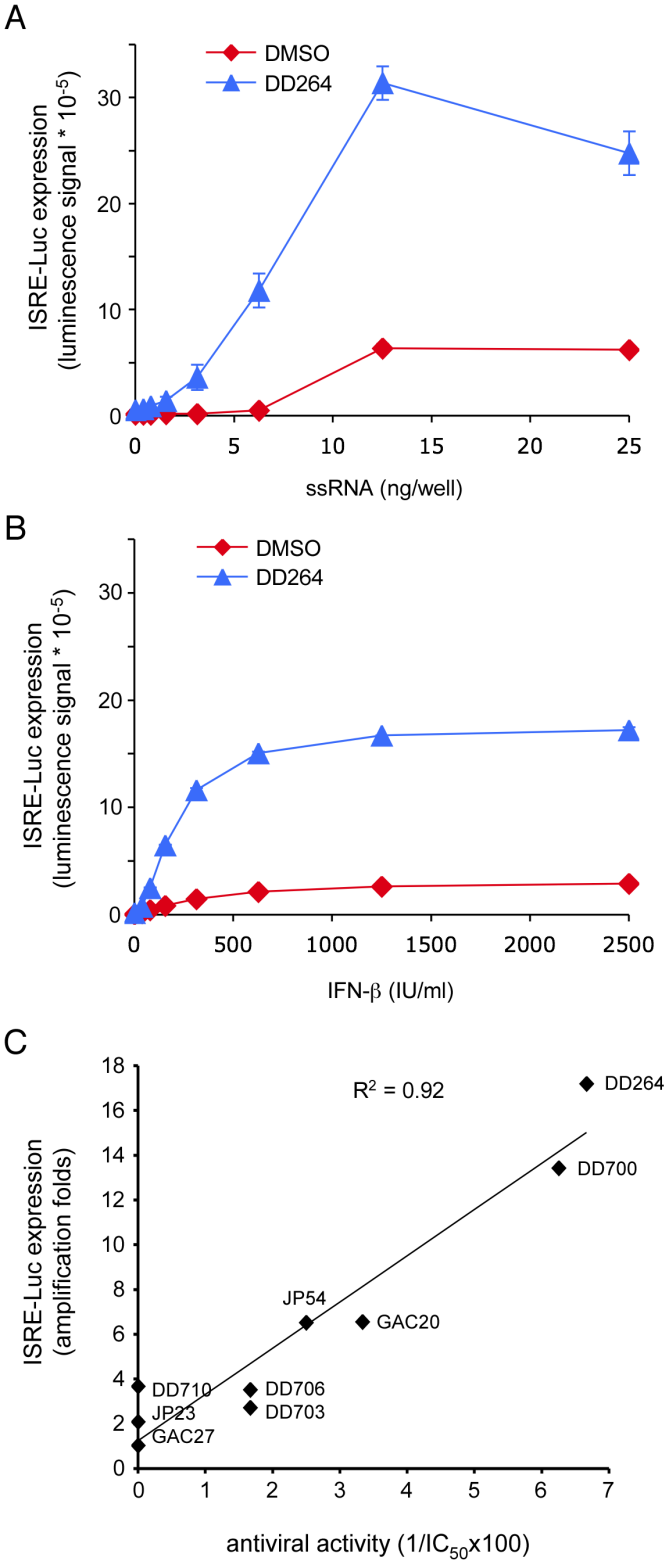
DD264 amplifies cellular response to transfection of PAMP-like ssRNA molecules or IFN-β stimulation, and this correlates with its antiviral activity. (**A**) HEK-293 cells with the ISRE-luciferase reporter gene (STING-37 cells) were transfected with increasing doses of synthetic 5′-triphosphate RNA molecules (ssRNA), and incubated in the presence of DD264 (80 µM) or DMSO alone in 96-well cultures plates. After 24 hours, luciferase expression was determined. (**B**) HEK-293 cells with the ISRE-luciferase reporter gene (STING-37 cells) were transfected with increasing doses of recombinant IFN-β, and incubated in the presence of DD264 (80 µM) or DMSO alone in 96-well culture plates. After 24 hours, luciferase expression was determined. Experiments (A) and (B) were performed in duplicate, and data represent means ± SD. (**C**) Eight molecules were randomly picked among analogs of DD264 (see Table S2) to build a set of compounds with a range of antiviral activities from null to high as determined by their potency to inhibit MV-Luc replication. Then, the selected molecules were tested for the amplification ISRE-luciferase expression when stimulating cells with suboptimal doses of ssRNA (6 ng/well) as described in (A). Finally, for DD264 and selected analogs, the capacity to amplify cellular response to ssRNA was plotted as a function of the antiviral activity by means of the experimental IC_50_ values (antiviral activity = 1/IC_50_*100).

Despite a poor capacity to induce the ISRE-luciferase reporter gene by itself, DD264 has a broad-spectrum antiviral activity. This led us to hypothesize that DD264 rather amplifies the cellular response to viral PAMPs and/or IFN signaling, which would account for its potent antiviral activity. To test this hypothesis, cells stably transfected with the ISRE-luciferase reporter gene were transfected with increasing doses of short synthetic 5′-triphosphate RNA molecules (ssRNA) or treated with recombinant IFN-β, then incubated in the presence of DD264 or DMSO alone. Because ssRNA molecules mimic a viral PAMP, they activated the host antiviral response and induced expression of the ISRE-luciferase reporter gene (Figure 3A). Cellular response to suboptimal doses of ssRNA or IFN-β was strongly amplified in the presence of DD264 (Figure 3A and 3B). Interestingly, ISRE-luciferase induction by ssRNA transfection was partially dependent on the IFN-α/β secretion loop, as assessed by the addition of blocking antibodies against these cytokines (Figure S4A). However, amplification of cellular response by DD264 was still observed when blocking antibodies were added, establishing that DD264 activity is essentially independent of the IFN-α/β loop (Figure S4A). Accordingly, DD264 antiviral activity was not affected by the same cocktail of antibodies directed against IFN-α/β (Figure S4B). We also tested a subset of DD264 analogs ranging from strong antiviral to inactive molecules for their capacity to amplify cellular response to ssRNA. A clear correlation was found between their antiviral activity and their capacity to amplify cellular response to ssRNA (Figure 3C), thus supporting a functional link between these two activities.

**Figure 4:**
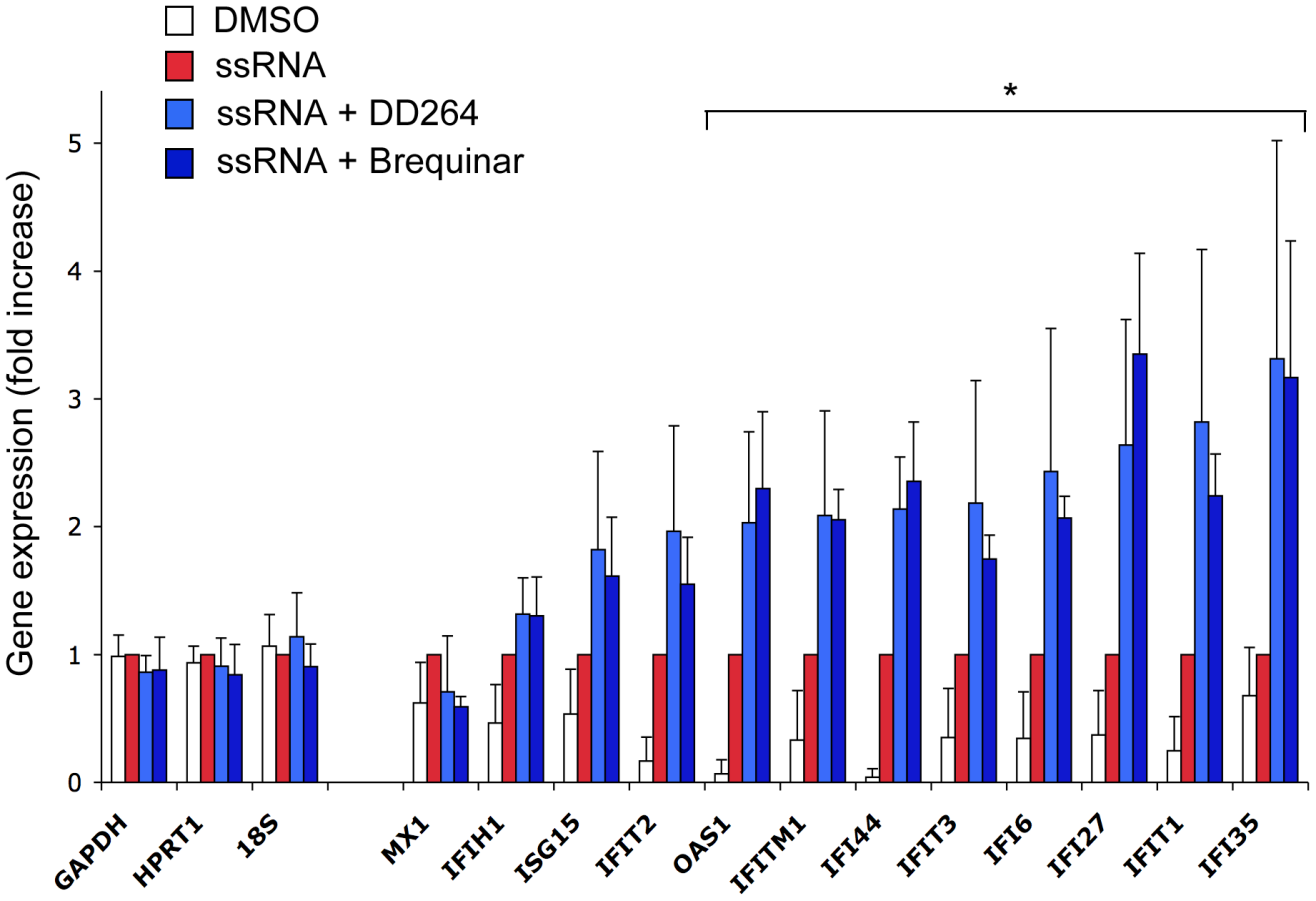
Expression of IFN-inducible genes (ISGs) is amplified by DD264 and brequinar. HEK-293 cells with the ISRE-luciferase reporter gene (STING-37 cells) were transfected with suboptimal doses of ssRNA (50 ng/well) in 24-well culture plates, and incubated in the presence of DD264 (40 µM), brequinar (100 nM) or DMSO alone. After 24 h of culture, total RNAs were extracted, and expression levels of indicated genes were quantified by qRT-PCR. *GAPDH*, *HPRT1* and *18S* correspond to control housekeeping genes, whereas others are well-known ISGs. Experiment was performed four times and for each gene, data were normalized using cells stimulated with ssRNA alone as a reference (red bars).

To further support this observation, we determined by quantitative RT-PCR the transcriptional level of twelve interferon-stimulated genes (ISGs) when activating cells with ssRNA (Figure 4). We observed a significant 2 to 3-fold increase in expression levels of most tested ISGs when ssRNA-transfected cells were cultured in the presence of DD264. In contrast, control genes like *18S*, *GAPDH* or *HPRT1* were not affected. Altogether, this demonstrated that DD264 amplified cellular response to ssRNA and expression levels of antiviral genes, and this correlated with its antiviral activity.

### DD264 is an inhibitor of pyrimidine biosynthesis

**Figure 5:**
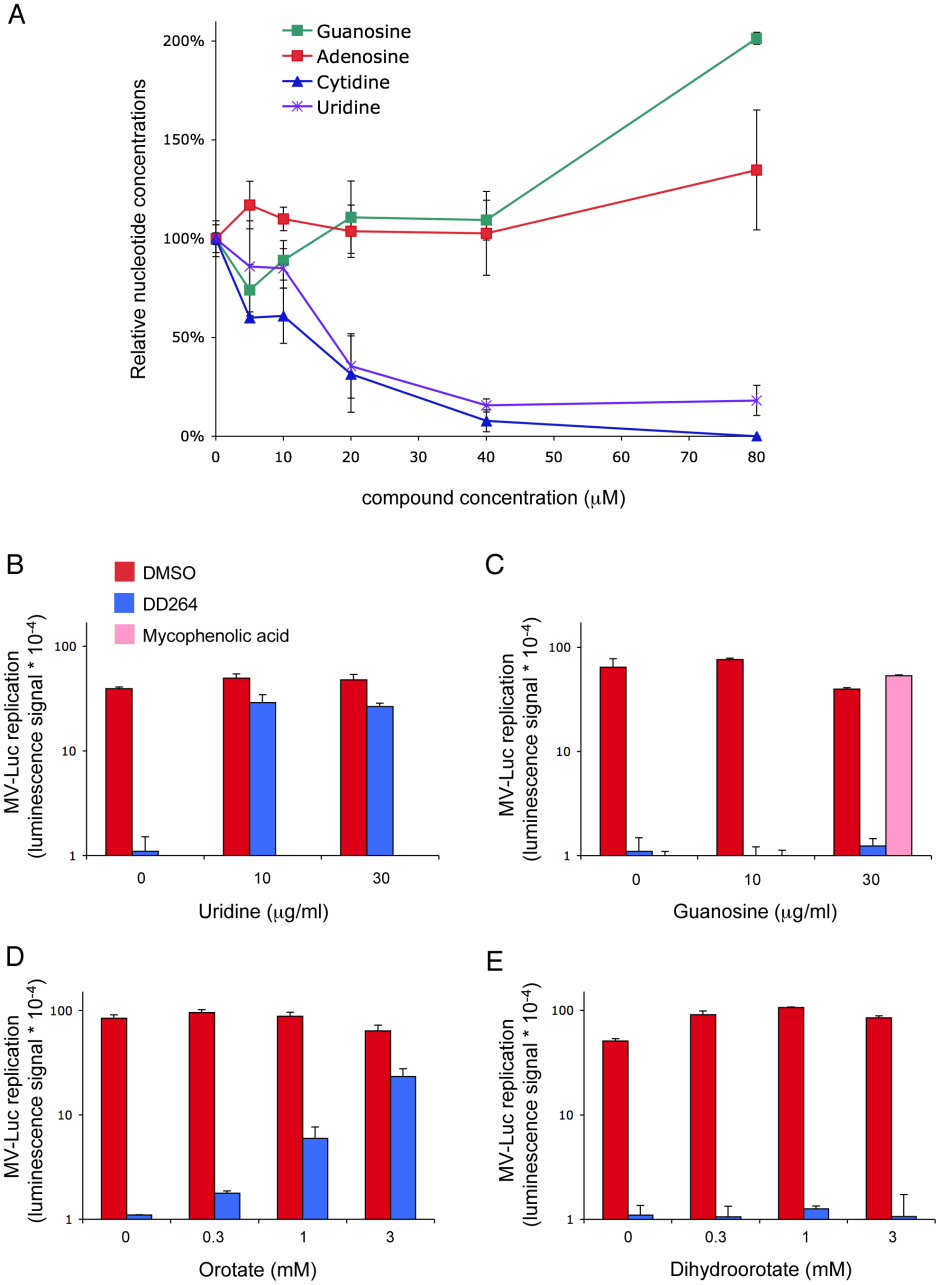
DD264 is an inhibitor of pyrimidine biosynthesis pathway. (**A**) Inhibition of uridine and cytidine in DD264-treated cells. HEK-293T cells were treated with DMSO alone or increasing concentrations of DD264. After 24 hours, cells were harvested and nucleoside concentrations were determined by HPLC. Concentration is expressed as a percentage relative to DMSO-treated cells. (**B–E**) HEK-293T cells were infected with recombinant MV strain expressing luciferase (MOI = 0.1), and incubated with DD264 (40 µM), mycophenolic acid (15 µM) or DMSO alone, and culture medium was supplemented with uridine (**B**), guanosine (**C**), orotate (**D**) or dihydroorotate (**E**). After 24 hours, luciferase expression was determined. Experiment was performed in duplicate, and data represent means ± SD.

Several recent reports have shown that inhibitors of pyrimidine biosynthesis pathway are potent broad-spectrum antiviral molecules [33], [34], [35], [36]. Although DD264 is structurally unrelated to these molecules, we tested if DD264 could interfere with this particular metabolic pathway. We first determined intracellular concentrations of purine and pyrimidine nucleosides by HPLC and spectrophotometry analysis. As shown in Figure 5A, DD264 treatment decreased intracellular concentrations of uridine and cytidine, whereas purines were either unchanged (adenosine) or slightly increased (guanosine) likely as a consequence of the control loops connecting purine and pyrimidine metabolic pathways. Similar changes in nucleosides profile were previously reported in RKO cells treated with leflunomide, which is a clinically used inhibitor of pyrimidine biosynthesis pathway [37].

To determine whether inhibition of pyrimidine biosynthesis by DD264 is actually linked to viral growth inhibition, cells infected with MV-luciferase were treated or not with DD264 and the culture medium was either supplemented or not with increasing doses of pyrimidine (uridine) or purine (guanosine) nucleosides. Adding uridine fully restored viral replication in DD264-treated cells as assessed by luciferase expression (Figure 5B), whereas guanosine had no effect (Figure 5C). Conversely, guanosine but not uridine abolished the antiviral activity of mycophenolic acid, a molecule that was previously reported to block viral replication by inhibiting purine biosynthesis [38], [39], [40]. Altogether, this demonstrated that a lowered level of pyrimidine nucleosides is responsible for the antiviral activity of DD264.

Different intermediates of the pyrimidine nucleosides *de novo* biosynthesis pathway were added to the culture medium to seek for those able to counteract the inhibition of viral growth by DD264. It turned our that in DD264-treated cells, MV replication was restored in the presence of orotate but not dihydroorotate (Figure 5D and 5E). Along pyrimidine biosynthesis, dihydroorotate is oxidized into orotate by dihydroorotate dehydrogenase (DHODH), the fourth enzyme of this metabolic pathway. Although this is not a formal demonstration because we could not control for equal entry and stability of orotate and dihydroorotate in HEK-293T cells, these data strongly suggested that within pyrimidine biosynthesis pathway, DHODH is the target of DD264. This enzyme has also been described recently as the cellular target for several newly identified compounds with a broad-spectrum antiviral activity [33], [34], [35], [36].

### Inhibition of pyrimidine synthesis induces the amplification of cellular response to ssRNA

**Figure 6:**
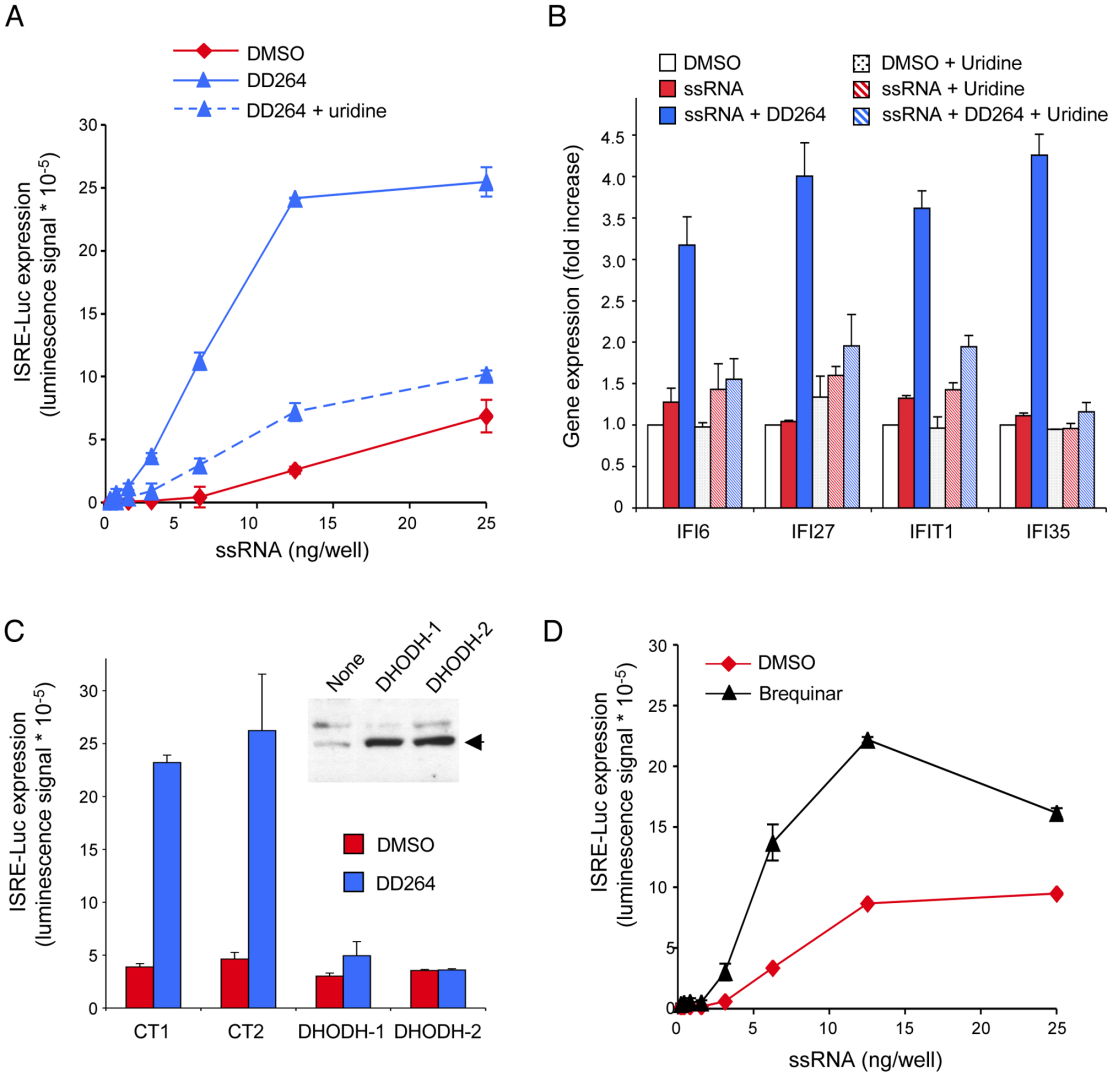
Inhibition of pyrimidine biosynthesis accounts for the amplified cellular response to ssRNA. (**A**) HEK-293 cells with the ISRE-luciferase reporter gene (STING-37 cells) were transfected with increasing doses of ssRNA, and incubated in the presence of DD264 (80 µM) or DMSO alone in 96-well cultures plates. Culture medium was supplemented or not with uridine. After 24 hours, luciferase expression was determined. Experiment was performed in triplicate, and data represent means ± SD. (**B**) Cells were transfected with 20 ng/well of ssRNA in 12-well plates, and incubated in the presence of DD264 (80 µM) or DMSO alone. Culture medium was supplemented or not with uridine. After 24 hours, expression levels of IFI6, IFI27, IFIT1 and IFI35 were determined by qRT-PCR. Data were normalized relative to control housekeeping genes (GAPDH, HPRT1, and 18S). Experiment was performed in duplicate, and data represent means ± SD. (**C**) HEK-293 cells with the ISRE-luciferase reporter gene (STING-37 cells) were transfected in 96-well plates with 50 ng of an expression vector encoding for DHODH (using two independent plasmid preparations of the same construct, #1 and #2). DHODH overexpression was assessed by western-blot analysis (upper right panel). Alternatively, cells were transfected with expression vectors encoding for control proteins (CT1 and CT2, see Materials and Methods for details). After 48 hours, cells were transfected with 6 ng/well of ssRNA, and incubated in the presence of DD264 (80 µM) or DMSO alone. After 24 hours, luciferase expression was determined. Experiment was performed in duplicate, and data represent means ± SD. (**D**) HEK-293 cells with the ISRE-luciferase reporter gene (STING-37 cells) were transfected with increasing doses of ssRNA, and incubated in the presence of brequinar (200 nM) or DMSO alone in 96-well cultures plates. After 24 hours, luciferase expression was determined. Experiment was performed in duplicate, and data represent means ± SD.

We have shown that the antiviral activity of DD264 relies on some imbalance in the pool of pyrimidines, which is mediated by the inhibition of the pyrimidine biosynthesis pathway probably *via* DHODH. However, DD264 was originally identified for its capacity to amplify expression of ISRE-regulated genes. We thus sought for a functional relationship existing between these two mechanisms. We first determined the ISRE-luciferase expression when culture medium was supplemented with uridine. As shown in Figure 6A, DD264-mediated amplification of cellular response to ssRNA was impaired in the presence of uridine. Similarly, uridine abolished the amplification effect of DD264 on the expression of ISGs in cells treated with low doses of ssRNA (Figure 6B). Finally, we determined if DHODH overexpression could compensate for DD264 treatment, and prevent the amplification of ISRE-luciferase expression in ssRNA-transfected cells. As shown in Figure 6C, the effect of DD264 on ISRE-luciferase expression was abolished when DHODH was overexpressed. This established a functional link between the lack of pyrimidine nucleosides in cells treated with DD264 and the amplification of cellular response to ssRNA. To further demonstrate that DHODH inhibition could amplify cellular response to ssRNA, we tested in our functional assay a well-known inhibitor of this enzyme: brequinar [41]. Cells were transfected with increasing doses of ssRNA and culture medium was supplemented with brequinar or DMSO alone. As shown in Figure 6D, brequinar amplified cellular response to ssRNA in a similar manner to DD264. Accordingly, brequinar also increased the expression of ISGs when stimulating cells with ssRNA (Figure 4). Altogether, these results demonstrated that inhibition of pyrimidine biosynthesis directly amplified cellular response to ssRNA and expression of ISGs.

### Antiviral potency of DD264 relies on cellular gene expression, nuclear export machinery and IRF1 transcription factor

**Figure 7:**
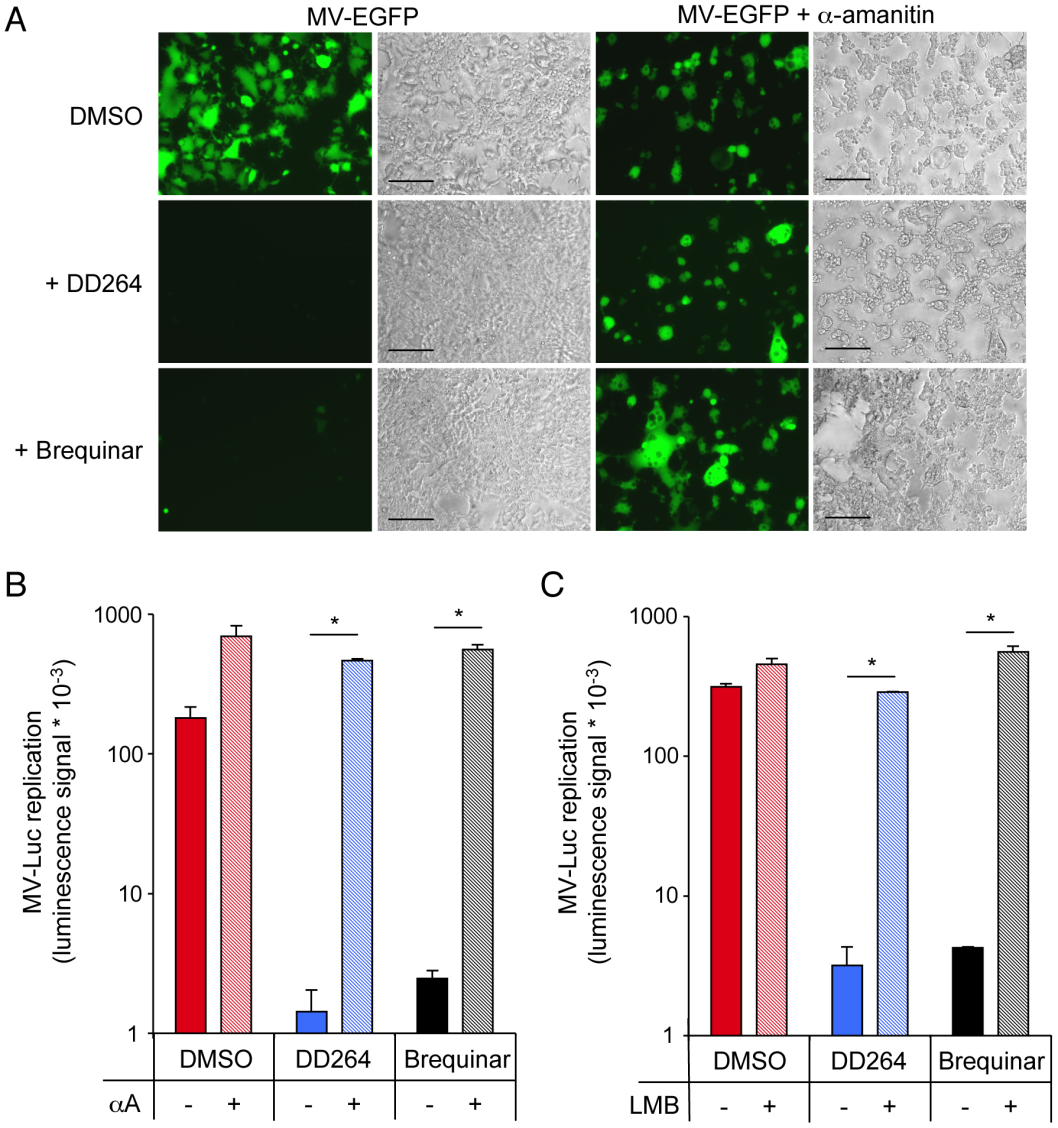
Antiviral activity of DD264 and brequinar is abrogated by α-amanitin and leptomycin B. (**A**) HEK-293T cells were infected with a recombinant strain of MV expressing EGFP (MOI = 0.1), and incubated with DD264 (40 µM), brequinar (200 nM) or DMSO alone (None), and culture medium was supplemented or not with α-amanitin (5 µg/ml). After 48 hours, EGFP expression in cell cultures was determined using fluorescence (left panels) and bright-field (right panels) microscopy. Scale bar = 200 µm. (**B**) HEK-293T cells were infected with recombinant MV strain expressing luciferase (MOI = 0.1), and incubated with DD264 (80 µM), brequinar (200 nM) or DMSO alone, and culture medium was supplemented or not with α-amanitin at 5 µg/ml (+/− αA). After 24 hours, luciferase expression was determined. Experiment was performed in duplicate, and data represent means ± SD. (**C**) HEK-293T cells were infected with recombinant MV strain expressing luciferase (MOI = 0.1), and incubated with DD264 (80 µM), brequinar (200 nM) or DMSO alone, and culture medium was supplemented or not with leptomycin B at 2.5 ng/ml (+/− LMB). After 24 hours, luciferase expression was determined. Experiment was performed in duplicate, and data represent means ± SD. * corresponds to p-values<0.05.

We have established a link between pyrimidine biosynthesis pathway and innate immune response. This opened the possibility that antiviral activity of DD264 is not a direct consequence of pyrimidine nucleoside deprivation that prevents viral transcription or replication, but rather relies on the amplification of the host antiviral response. To test this hypothesis, we treated HEK-293T cells with α-amanitin, a molecule that inhibits human RNA polymerase II and blocks transcription of cellular genes. As expected, α-amanitin showed some toxicity but did not impair MV replication. Most interestingly, MV inhibition by DD264 was abrogated in the presence of α-amanitin (Figure 7A and 7B), thus demonstrating that the antiviral activity of DD264 required the transcription of cellular genes. Similarly, α-amanitin blocked the antiviral activity of brequinar (Figure 7A and 7B). This argued against a direct impact of pyrimidine deprivation on viral replication, and rather involved the host response as assessed by the need for cellular gene expression.

This conclusion was further supported by experiments performed with leptomycin B (LMB), a potent inhibitor of Crm1-dependent nuclear export. Since cellular gene transcription is required for DD264 or brequinar to block viral growth, we reasoned that inhibiting mRNA export out of the nucleus should have similar effects. Thus, HEK-293T cells were infected with MV strain expressing luciferase, and cultured with DD264 or brequinar in the presence of LMB. As shown in Figure 7C, LMB efficiently restored viral replication in DD264 or brequinar-treated cells. Together with experiments performed with α-amanitin, this clearly involved both cellular gene transcription and nuclear export in the antiviral activity of DD264 and brequinar.

**Figure 8:**
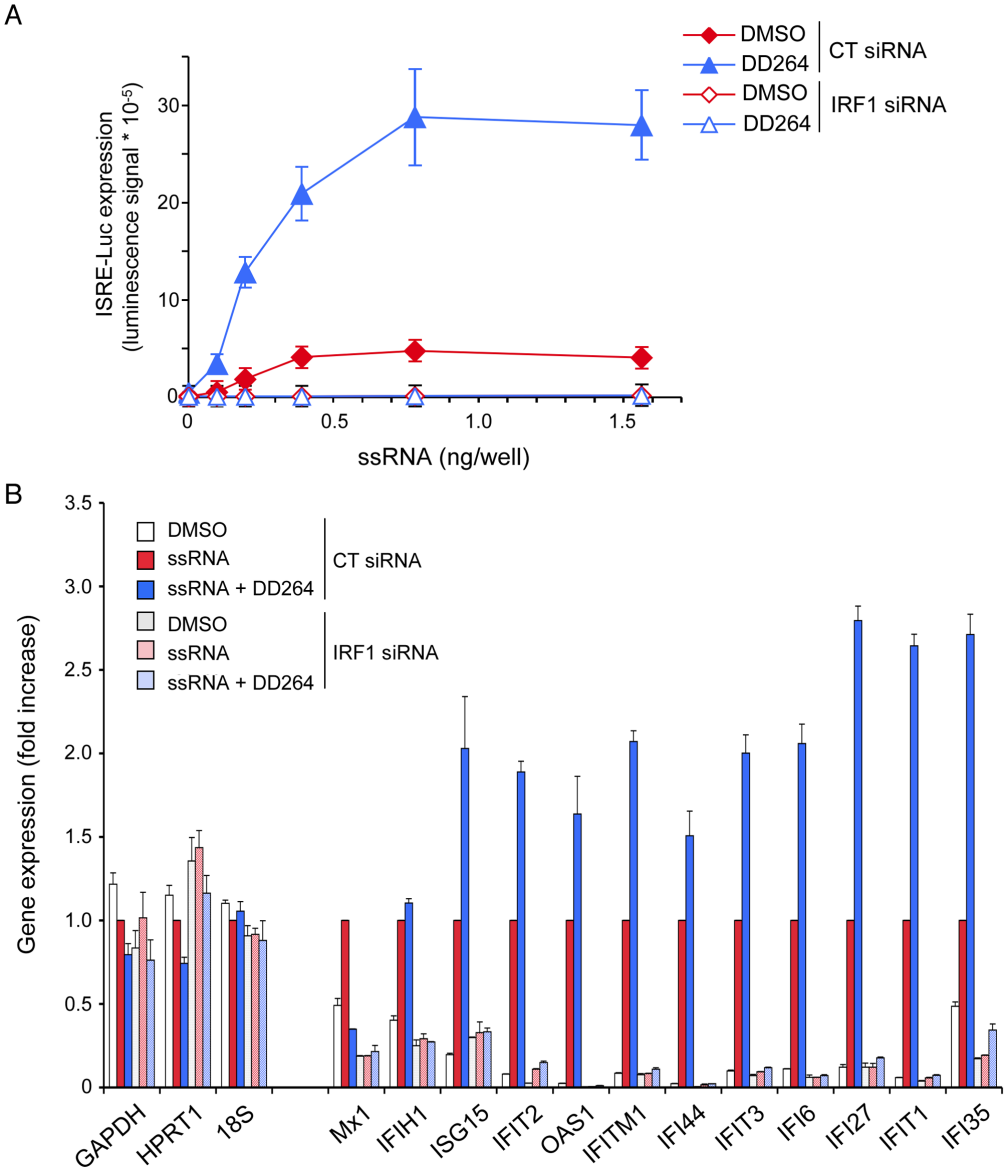
IRF1 is required for the expression of ISGs. (**A**) HEK-293 cells with the ISRE-luciferase reporter gene (STING-37 cells) were transfected with control siRNA (CT) or siRNA directed against *IRF1* and cultured for 48 hours in 96-well culture plates. Then, cells were transfected with increasing doses of ssRNA, and incubated in the presence of DD264 (80 µM) or DMSO alone. After 24 hours, luciferase expression was determined. Experiment was performed in triplicate, and data represent means ± SD. (**B**) HEK-293 cells with the ISRE-luciferase reporter gene (STING-37 cells) were transfected with control siRNA (CT) or siRNA directed against *IRF1* and cultured for 48 hours in 12-well culture plates. Cells were transfected with ssRNA (20 ng/well), and incubated in the presence of DD264 (40 µM) or DMSO alone. After 24 h of culture, total RNAs were extracted, and expression levels of indicated genes were quantified by qRT-PCR. *GAPDH*, *HPRT1* and *18S* correspond to control housekeeping genes, whereas others are well-known ISGs. Data were normalized using cells stimulated with ssRNA alone as a reference (red bars).

**Figure 9:**
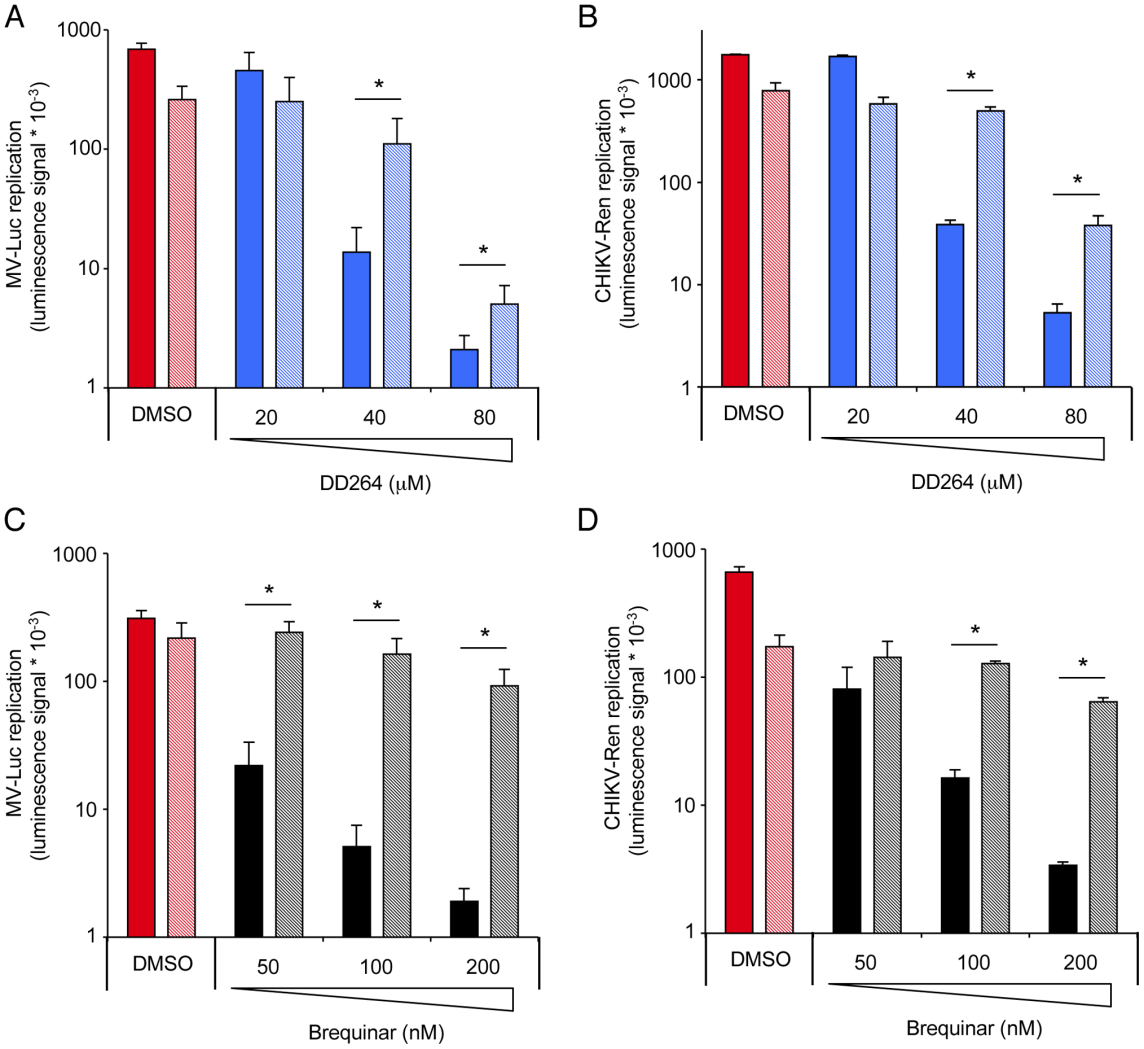
Antiviral activity of DD264 and brequinar is abrogated when silencing IRF1 expression in HEK-293T cells. (**A–B**) HEK-293T cells were transfected with control siRNA (solid colors) or siRNA directed against *IRF1* (shaded colors) and cultured for 48 hours. Then, cells were infected with recombinant MV strain expressing luciferase (MOI = 0.1) or CHIKV strain expressing luciferase (MOI = 0.2), and incubated with increasing concentrations of DD264 or DMSO alone. After 24 hours, luciferase expression was determined. Experiment was performed in triplicate, and data represent means ± SD. (**C–D**) Same experiment was performed but cells were treated with increasing concentrations of brequinar instead of DD264. * corresponds to p-values<0.05.

Expression of ISGs is essentially controlled by members of the interferon regulatory transcription factor (IRF) family that bind IRE (IRF regulatory elements) or ISRE in promoter sequences of antiviral genes. In particular, expression of IRF1 was shown to drive the expression of many ISGs and confer resistance to various viruses [16], [42], thus demonstrating a key role in host resistance to viral infections. Therefore, we investigated the role of IRF1 in ISRE-luciferase and ISG expression, and in the antiviral state induced by DD264. First, *IRF1* gene silencing by siRNA transfection was found to abolish ISRE-luciferase expression in ssRNA-treated cells, whether DD264 was added or not (Figure 8A). It should be noticed that when compared to Figure 3A, lower amounts of ssRNA were required to stimulate the ISRE-luciferase, suggesting that siRNA transfection had somehow sensitized cells to ssRNA in line with a recent report [43]. Furthermore, IRF1 silencing also suppressed the induction of various ISGs in ssRNA-treated cells, whether DD264 was added or not, whereas housekeeping genes were not affected (Figure 8B). Similarly, IRF1 silencing suppressed the induction of ISGs by MV infection (Figure S5A–L). Altogether, this demonstrated that IRF1 is essential for the expression of ISGs in these cells. If the antiviral activity of DD264 relied on the amplification of ISG expression as we hypothesized, knocking-down IRF1 was expected to suppress its antiviral activity. As shown in Figure 9A and 9B, IRF1 silencing partially restored MV and CHIKV replication in DD264-treated HEK-293T cells. IRF1 knockdown also restored viral growth in brequinar-treated cells (Figure 9C and 9D). Similar results were obtained when performing this experiment with HeLa cells, establishing that DD264 or brequinar antiviral activity required IRF1 expression in both cell types (Figure S6A–D). As a control, IRF1 expression levels in siRNA-treated HEK-293T or HeLa cells are presented in Figure S7A–B. This demonstrated the critical role of IRF1 in the antiviral state induced by DD264 or brequinar. Most importantly, this established that pyrimidine synthesis inhibitors, and in particular DHODH inhibitors, prevent viral replication by promoting antiviral gene expression, thus delineating a novel link between this metabolic pathway and innate immunity.

## Discussion

Here, we describe DD264, a molecule that was selected from a high-throughput functional screen for its capacity to stimulate expression of interferon-inducible antiviral genes. This compound demonstrated a strong and broad antiviral activity that correlated with its capacity to amplify cellular response to ssRNA and expression levels of antiviral genes. We further showed that DD264 immuno-stimulatory activity depends on the inhibition of pyrimidine biosynthesis, similarly to brequinar, a well-characterized inhibitor of this metabolic pathway. Altogether, this allowed us to establish a yet unsuspected link between inhibition of pyrimidine biosynthesis pathway and stimulation of innate immunity.

It is surprising that several groups, who recently performed functional screenings for inhibitors of viral growth in infected cells, all independently identified molecules targeting pyrimidine biosynthesis pathway and in particular DHODH [33], [34], [35], [36]. This suggests a strong bias for inhibitors of this enzyme when looking for antiviral molecules with such functional assays. Altogether, our results support the idea that the antiviral activity of pyrimidine synthesis inhibitors, including aforementioned compounds [33], [34], [35], [36], does not simply rely on depriving viral polymerases of nucleosides, but is mediated through amplification of innate immune response. Conversely, other research groups have isolated broad-spectrum antiviral molecules while searching for stimulators of innate antiviral genes much like we did, using cell-based assays with reporter genes [9], [21], [22], [23], [24], [25]. With our results in mind, it should be determined if the antiviral molecules they identified based on their capacity to stimulate interferon-inducible antiviral genes are in fact pyrimidine synthesis inhibitors, or if they stimulate innate immunity through alternative pathway.

Our data show that antiviral activity of pyrimidine synthesis inhibitors such as DD264 or brequinar, although independent on IFN-α/β secretion (Table S1 and S2; Figure S4), critically relies on the expression of cellular genes as assessed by experiments performed with α-amanitin, leptomycin B and siRNA targeting IRF1. IRFs are transcription factors that bind ISRE and closely related IRE elements in the promoter region of target genes. Most IRFs are critically involved in the regulation of immune response [44], [45], [46], [47], [48], [49], [50]. Since IRF1 is a master regulator of antiviral gene expression, as supported by literature [16], [42] and confirmed by our own data, its implication in virus inhibition by DD264 and brequinar is consistent. But how inhibition of pyrimidine synthesis stimulates innate immune response remains a pending question. Interestingly, our data parallel a previous work showing that inhibition of pyrimidine synthesis induces a cellular stress that translates into p53 up-regulation and nuclear accumulation [37]. In this system, NAD(P)H:quinone oxidoreductase 1 (NQO1) and NRH:quinone oxidoreductase 2 (NQO2) induced p53 upregulation by preventing its degradation in the 20S proteasome. We did not detect any upregulation of IRF1, as assessed by qRT-PCR and western-blot (Table S1 and Figure S8A) or modification of its nuclear localization pattern in DD264 or brequinar-treated cells (Figure S8B), which could account for the increased activity of the ISRE promoter. However, IRF1 binds numerous cellular proteins, and these interactions can regulate the transcriptional activity of both IRF1 and associated transcription factors like p53 or NF-kB [51], [52], [53], [54], [55]. Besides, post-translational modifications of IRF1, like ubiquitination, SUMOylation, and acetylation, have been reported to modulate its activity [53], [56], [57]. Associations with cofactors or post-translational modifications could increase IRF1 transcriptional activity in DD264 or brequinar-treated cells, and this could explain the enhanced expression of ISGs when blocking pyrimidine biosynthesis.

Another interesting observation came from the analysis of other nucleoside synthesis inhibitors, in particular mycophenolic acid. This potent inhibitor of inosine monophosphate dehydrogenase (IMPDH) blocks the *de novo* synthesis of purine nucleosides, and is also known for its broad-spectrum antiviral activity since the 60's [38]. In a recent report by van der Laan and colleagues, mycophenolic acid was shown to increase expression of various ISGs, including IRF1, when stimulating cells with low doses of IFN-α [58]. Furthermore, inhibition of hepatitis C virus replication by mycophenolic acid was shown partially mediated by IRF1. These data clearly match our observations on pyrimidine synthesis inhibitors. Altogether, this suggests that inhibition of either purine or pyrimidine synthesis mediates a cellular stress that promotes expression of ISGs and induces a resistance state to infections by RNA viruses. Interestingly, inhibition of pyrimidine synthesis was recently found to reverse the inhibition of mRNA nuclear export by different virulence factors such as influenza virus NS1 or the matrix protein of vesicular stomatitis virus [59]. How this relates to our findings will have to be addressed in the future.

Finally, although DD264 was found to inhibit the replication of various RNA viruses, this compound was inefficient to prevent cellular infection by two related DNA viruses: Herpes simplex virus type 1 (HSV1) and type 2 (HSV2) (Figure S9). This is surprising as pyrimidine biosynthesis inhibition was previously reported to block the replication of other DNA viruses like human adenovirus 5 or vaccinia virus [35]. This suggested that DNA viruses in general are sensitive to inhibitors of pyrimidine biosynthesis pathway, but HSV1 and 2 have apparently evolved some strategy to escape the cellular response induced by this type of drug. Indeed, herpes simplex viruses are well known for their large arsenal of virulence factors that block the antiviral response through different mechanisms [60]. Screening collections of HSV1 or 2 proteins to identify which ones are susceptible to alleviate the antiviral state induced by pyrimidine biosynthesis inhibitors should be relatively straightforward. This is probably a specific feature of *Alphaherpesvirinae* since human Cytomegalovirus (CMV), which belongs to *Betaherpesvirinae*, was found sensitive to the inhibition of pyrimidine biosynthesis [61]. Interestingly, it was also reported that CMV and HSV1 have divergent effects on cellular metabolism. Although CMV enhances lipid biosynthesis, HSV1 rather promotes the synthesis of pyrimidine nucleosides [62]. This could also account for a different sensitivity to pyrimidine biosynthesis inhibitors. Importantly, although pyrimidine biosynthesis inhibitor leflunomide was reported to block HSV1 replication, some off-target effect of this drug on kinases is most likely responsible for this activity [63].

Our data also strongly suggest that DD264 directly inhibits DHODH, the fourth enzyme of pyrimidine biosynthesis metabolic pathway. This mitochondrial enzyme catalyzes dihydroorotate oxidation to orotate via a two-step mechanism that requires FMN and ubiquinone as prosthetic groups [64], [65]. Best-known inhibitors of DHODH such as brequinar or A77-1726, the active form of leflunomide, are inserted within a hydrophobic tunnel of the enzyme where they probably compete with ubiquinone [41]. Interestingly, DD264 exhibits two hydrophobic rings and a planar structure that matches chemical features of DHODH inhibitors targeting the ubiquinone-binding pocket, and this was confirmed by *in silico* docking experiments (Figure S10A–C). This will require confirmation using enzymatic assays and structure analysis.

Whether DHODH represents a viable target for antiviral treatments *in vivo* will need to be determined. Several attempts to use pyrimidine synthesis inhibitors in the treatment of systemic viral infections have failed [33], [34], [36]. This is probably because the high uridine concentration in peripheral blood (about 3–8 µM) compensates for the inhibition of the *de novo* pyrimidine synthesis pathway. However, it has been shown that A77-1726, which blocks both kinase activity and *de novo* pyrimidine synthesis, could be useful in the treatment of airways when infected by respiratory viruses such as human respiratory syncytial virus [66], [67]. This suggests that DHODH inhibitors should be further evaluated *in vivo* in the local treatment of respiratory tract infections.

In conclusion, our data demonstrate that inhibition of pyrimidine synthesis increases expression of antiviral genes, which finally confers resistance to viral infections. In the future, physiological or pathological circumstances activating this transduction pathway will have to be determined.

## Materials and Methods

### Compound libraries and reagents

The compound collection amounts to a total of 41,353 molecules arrayed in 520 96-well microplates. About one third of the collection comprises commercial chemical libraries acquired through Prestwick Chemical (1,120 compounds; www.prestwickchemical.com) and ChemDiv (eu.chemdiv.com), which provided 9,360 compounds from a kinase inhibitor subset library (CDI) and 4,624 compounds from a nucleobase analog subset library (NECAN). The rest of the collection is from the French “Chimiothèque Nationale” [68]. All compounds were stored in DMSO at −20°C. Compounds from Prestwick Chemical and ChemDiv were at 2 mg/ml, which corresponds to 6.32±2.8 mM (Prestwick Chemical), 5.57±0.89 (CDI) and 5.35±1.11 mM (NECAN), respectively. Compounds from the “Chimiothèque Nationale” were at the following concentrations for the different subsets: Université de Lyon at 10 mM, Faculté de Pharmacie de Strasbourg at 2 mg/ml (7.43±2.48 mM), Centre d'Etude et de Recherche sur le Médicament de Normandie (CERMN) at 3.3 mg/ml (10.5±3.35 mM), Institut de Chimie des Substances Naturelles at 1 mg/ml (3.02±1.28 mM), Institut Curie at 2 mg/ml (7.34±2.76 mM), Institut Pasteur-A at 3.3 mg/ml (12.8±3.71 mM), Institut Pasteur-B at 2 mg/ml (6.81±2.13 mM), Mutabilis at 5 mM, and Novexel at 5 mg/ml (11.08±2.31 mM).

Brequinar sodium salt hydrate was from Sigma (SML0113). α-amanitin was from Sigma (A2263). Leptomycin B from Sigma (L2913) was kindly provided by Dr. Agata Budkowska (Institut Pasteur). Short synthetic 5′-triphosphate RNA molecules (ssRNA) were synthesized from pCI-neo vector digested with XbaI using T7 RiboMAX Express large scale RNA production system (Promega), and then purified with a filtering membrane (Millipore). ELISA kits to determine IFN-α and IFN-β levels in culture supernatants were from PBL Biomedical Laboratoris. ELISA kit to determine IFN-γ level in culture supernatants was from eBioscience. Sheep polyclonal antibodies against IFN-α (31100-1) and IFN-β (31400-1) were from PBL Biomedical Laboratories.

### Cell lines, primary cells and viruses

HEK-293T, HeLa and MRC5 cells (ATCC) were maintained in Dulbecco's modified Eagle's medium (DMEM; Gibco-Invitrogen) containing 10% fetal calf serum (FCS), penicillin, and streptomycin at 37°C and 5% CO2. Cellular viability was determined by quantification of adenosine triphosphate (ATP) in culture wells using the CellTiter-Glo Assay (Promega) following manufacturer's recommendations. Apoptosis was detected by TUNEL (terminal deoxynucleotidyltransferase dUTP nick end labeling) using the APO-Direct assay kit from BD. Cell cycling was determined by propidium iodide staining and flow cytometry analysis.

Chikungunya virus (CHIKV) infections were performed with wild-type strain 05115 from La Réunion Island. The recombinant CHIKV strain expressing Renilla luciferase from a coding sequence inserted between nsP3 and nsP4 non-structural proteins has already been described [28]. All CHIKV stocks were produced on VERO cells, and titrated by TCID50 (50% Tissue Culture Infective Dose) on HEK-293T cells. Recombinant measles virus strain expressing firefly luciferase (rMV2/Luc) or EGFP (rMV2/EGFP) from an additional transcription unit were derived from vaccine strain Schwarz, and have been previously described [69], [70]. Virus stocks were produced on VERO cells, and titrated by TCID50 on VERO cells. The highly neurovirulent West Nile virus (WNV) strain IS-98-ST1 was propagated in mosquito AP61 cells and virus titer was determined on primate VERO cells by virus plaque-forming assays. The titer of IS-98-ST1 virus stock was about 10^+10^ plaque-forming units (PFU) per millimeter. Cy3-conjugated antibody against CHIKV E2 protein was previously reported (Clone 3E4) [71]. Cy3-conjugated antibody against WNV E protein (Clone E24) was kindly provided by Dr. MS. Diamond. Coxsackievirus B3 (CVB3, strain Nancy) was kindly provided by Dr. M. Vignuzzi (Institut Pasteur). Virus was amplified on HeLa cells, harvested by one freeze–thaw cycle and titrated by TCID50. Herpes simplex virus type 1 (HSV1, strain KOS) and 2 (HSV2, strain VR734) were kindly provided by Dr. T. Mourez (Assistance Publique – Hôpitaux de Paris). Virus stock were produced on VERO cells, and titrated by TCID50. Anti-herpesvirus antibody was from Argene (Ref 11-090).

### Screening procedure

All robotic steps were performed on a TECAN Freedom EVO platform. All compounds were screened at a 1∶100 dilution of the original stock. Compounds were transferred from mother plates into white, flat bottom, bar-coded tissue culture 96-wells plates (Greiner Bio One): 1 µl of a DMSO solution was spiked into dry wells of daughter plates (80 compounds per plate). For each plate, columns 1 and 12 were dedicated to controls: culture wells were alternatively spiked with 1 µl of DMSO alone (negative control) or supplemented with recombinant IFN-β so that final concentration equals 1,000 IU/ml (positive control). Human HEK-293T cells were transfected in bulk with pISRE-luciferase reporter plasmid (Stratagene, Ref 219089). Cell transfection was performed with a 1 mg/ml poly-ethylenimine (PEI “Max”; Polyscience) solution adjusted at pH = 7. For one 96-well culture plate, 17 µg of plasmid were diluted in 500 µl of DMEM (Gibco-Invitrogen). In parallel, 53 µg of poly(ethyleneimine) from Sigma-Aldrich (PEI) were diluted in 500 µl of DMEM (Gibco-BRL). PEI and plasmids were mixed together and incubated for 30 min at room temperature. This mix was added to 2×10^6^ cells in a final volume of 10 ml of DMEM containing 10% fetal bovine serum, penicillin, and streptomycin. Finally, 100 µl of this cell suspension were added to each well of the microplate already containing one chemical compound. After 24 hours of incubation at 37°C in the presence of 5% CO2, the firefly luciferase substrate (Bright-Glo, Promega) was added directly into the wells (50 µl) and luciferase activity was measured 6 minutes later on a Safire2 (TECAN) using a 100 ms integration time. For each plate, means of luminescence and corresponding standard deviations were calculated for positive and negative controls (μ^+^, σ^+^, μ^−^, and σ^−^, respectively) to determine the signal-to-background ratio (S/B = μ^+^/μ^−^) and the Z′-factor (Z′-factor = 1–3*(σ^+^+σ^−^)/(μ^+^−μ^−^)). Average Z′-factor was determined to be 0.806±0.1 (no value below 0.5; Figure S1) and signal-to-background (S/B) ratio, which corresponds to luciferase signal in the presence of recombinant IFN-β relative to DMSO alone, was >45 for all plates. Altogether, this demonstrated the robustness of our assay, which can be categorized as excellent [72]. For each compound, the induction factor was calculated as the ratio of luminescence signal measured in the corresponding well to the mean of luminescence for negative controls in the same plate.

### Establishment of STING-37 stable cell line

We generated a reporter plasmid carrying both the ISRE-luciferase gene and *neo* as a G418-resistance selection marker. First, pCi-neo plasmid (Promega) was digested with BglII and XbaI enzyme to remove the entire CMV promoter sequence. Plasmid extremities were filled using Pfu polymerase, and Gateway cassette C1 (Invitrogen) was cloned between the two blunt ends to produce a Gateway-compatible destination vector called pCi-neoΔCMV-GW. In parallel, the ISRE-luciferase sequence was amplified by PCR from pISRE-luciferase reporter plasmid (Stratagene, Ref 219089) using Gateway (Invitrogen) primers AttB1-AACGTTATTTTTCACTGCATTCTAG and AttB2-AGATCTCACTGCTCCCATTCATCAG. Corresponding DNA fragment was cloned by *in vitro* recombination (BP reaction) into pDONR207 entry vector. ISRE-luciferase gene was finally recombined from pDONR207 into pCi-neoΔCMV-GW by *in vitro* recombination (LR reaction). This new plasmid was transfected in HEK-293 cells (ATCC) using JetPrime reagent (Polyplus Transfection). Two days later, culture medium was removed and replaced by fresh medium containing G418 at 500 µg/ml. Transfected cells were amplified and subsequently cloned by serial limit dilution. A total of 44 clones were screened for luciferase activity, and STING-37 clone was selected for its optimal signal to background ratio when stimulated or not with recombinant IFN-β.

### DHODH expression vector

DHODH sequence was amplified by PCR (Ex-Taq; Takara) from an IMAGE full-length cDNA clone (IMAGE ID: 6064723), and cloned into pDONR207 using an *in vitro* recombination-based cloning system (Gateway technology; Invitrogen). DHODH encoding sequence was subsequently transferred from pDONR207 into a modified pCI-neo vector (Promega) compatible with the Gateway system. Identical plasmids encoding for either cellular protein UBA3 or nsP4 of chikungunya virus were used as transfection controls (CT1 and CT2, respectively). Transfection was performed with jetPRIME reagent (Polyplus Transfection) following manufacturer's recommendations. Anti-DHODH monoclonal antibody was from Abnova (Clone 6E1).

### IRF1 siRNA procedure

Silencer Select siRNA were purchased from Invitrogen, and transfected in STING-37 cells following manufacturer's recommendations. IRF1 silencing was achieved with a pool of two siRNA (s7502 and s7503), whereas controls correspond to a pool of two siRNA directed against IRF5 (s7513 and s7515). In each well of a 96-well plate, 2 pmol of siRNA were mixed with 20 µL of Opti-MEM (Gibco-Invitrogen) and 0.25 µL of Lipofectamine RNAiMAX transfection reagent (Invitrogen). This mix was incubated for 10 minutes at room temperature, and supplemented with 80 µL of DMEM +10% FCS without penicillin and streptomycin and 15,000 STING-37 cells. Cells were incubated for 48 hours at 37°C and 5% CO2, and then stimulated with DD264 and recombinant IFN-β at 500 IU/ml or transfected with ssRNA molecules with JetPrime. After 24 hours of culture, firefly luciferase activity was determined using the Bright-Glo reagent following manufacturer's recommendations (Promega). Anti-IRF1 antibody was from Cell Signaling (D5E4).

### Quantitative RT-PCR analysis

HEK-293T cells were plated in 24-well plates (2×10^5^ cells per well). One day later, cells were transfected with 50 ng/well of ssRNA molecules. Transfections were performed with JetPrime PEI (Polyplus transfection), and stimulated or not in the presence of DD264. 24 hours later, cells were recovered in PBS and total RNA isolated with the RNeasy Mini Kit (Qiagen) according to manufacturer's protocol. Following elution, RNA yields were evaluated using a Nanodrop spectrophotometer (Nanodrop technologies).

A two-step qRT-PCR (Taqman technology, Applied Biosystems) was performed to measure transcription levels for 11 genes of interest (primer references are indicated): *IFI27* (Hs00271467_m1), *IFI35* (Hs00413458_m1), *IFI44* (Hs00197427_m1), *IFI6* (Hs00242571_m1), *IFIH1* (Hs01070332_m1), *IFIT1* (Hs01911452_s1), *IFIT3* (Hs01922752_s1), *IFITM1* (Hs00705137_s1), *ISG15* (Hs01921425_s1), *MX1* (Hs00895608_m1) and *OAS1* (Hs00973637_m1). Expression levels of four housekeeping genes, including *18S* (Hs99999901_s1), *GAPDH* (Hs99999905_m1), and *HPRT1* (Hs99999909_m1), were also determined and used as internal reference controls. Starting from 1 µg of total RNA, cDNA synthesis was achieved in 20 µL using the SuperScript VILO cDNA Synthesis Kit following manufacturer's recommendations (Life Technologies). Quantitative PCR reactions were performed on 0.6 µL of cDNA synthesis reaction mix using the TaqMan Fast Advanced Master Mix (Applied Biosystems) on a StepOnePlus Real-Time PCR machine (Applied Biosystems). Results were normalized using expression levels of the four housekeeping genes.

Transcription levels of IRF1 and IFN genes presented in Table S1 were determined with a two-step qRT-PCR (SYBR green technology, SABiosciences) using RT^2^ Profiler PCR Array System. Starting from 5 µg of total RNA, cDNA synthesis was achieved using RT^2^ First-Stand cDNA Synthesis Kit following manufacturer's recommendations (SABiosciences). Quantitative PCR reactions were performed using RT^2^ qPCR SYBR Green Master Mix (SABiosciences).

### Metabolite analyses

HEK-293T cells were plated in 6-well plates (1×10^6^ cells per well). One day later, culture medium was supplemented with increasing doses of DD264 or DMSO alone. After an additional 24 hours of culture, cells were harvested, carefully counted and monitored for viability by trypan blue exclusion, and washed with ice-cold phosphate-buffered saline (PBS). All the extraction steps were performed on ice. Cellular pellets were deproteinized with an equal volume of 6% trichloroacetic acid (TCA), vortex-mixed for 20 seconds, ice-bathed for 10 min, and vortex-mixed again for 20 seconds. Acid cell extracts were centrifuged at 13,000 rpm for 10 min at 4°C. The resulting supernatants were supplemented with an equal volume of bi-distilled water, vortex-mixed for 60 seconds, and neutralized by the addition of 5M K_2_CO_3_. Cells extracts were subjected to an optimised SPE procedure as previously described [73]. All fractions were injected into the HPLC system separately and the results pooled to calculate the total amount of nucleotides and nucleosides present in the original samples. HPLC analysis was performed with a Shimadzu HPLC system interfaced to the LabSolution software. Samples were injected onto an ABZ Supelco 5 µm (150×4.6 mm) column (Sigma). The HPLC columns were kept at 40°C in a column oven. The mobile phase was delivered at a flow-rate of 1 ml/min during the analysis using a stepwise isocratic elution with a buffer containing 10 mM acetate ammonium adjusted to pH 6.9. Detection was done with the diode array detector (PDA). The LC Solution workstation chromatography manager was used to pilot the HPLC instrument and to process the data. The products were monitored spectrophotometrically at 254 nm and quantified by integration of the peak absorbance area, employing a calibration curve established with various known concentrations of nucleosides. Finally, a correction coefficient was applied to correct raw data for minor differences in the total number of cells determined in each culture condition.

## Supporting Information

Figure S1 **Evaluation of the screen using the Z′-factor value plotted for each plate.**
(PDF)

Figure S2 **DD264 inhibits the proliferation of HEK-293T cells.** (**A**) HEK-293T cells were incubated with increasing doses of DD264 (10, 20, 40 or 80 µM) or matching volumes of DMSO alone. After 0, 24, 48 and 72 hours of culture, the number of living cells was determined using CellTiter-GLO reagent (Promega). This luciferase-based assay evaluates by ATP quantification the number of metabolically active cells in culture wells. The number of living cells is expressed as a percentage relative to the initial number of living cells at t = 0 hours. (**B**) HEK-293T cells were incubated with DD264 at 80 µM or DMSO alone. After 24 h, cells were stained for DNA fragmentation by TUNEL (Terminal deoxynucleotidyl transferase dUTP nick end labeling). Alternatively, cells were stained with propidium iodide for cell cycle analysis by flow cytometry, and percentage of cells in G2/M phase is indicated.
(PDF)

Figure S3 **DD264 inhibits MV and CVB3 growth.** (**A**) HEK-293T cells were infected with MV (MOI = 0.2) and then cultured for 48 hours with DMSO or DD264 at 80 µM. Cell cultures were harvested by scraping, and then frozen and thawed to release viral particles. Supernatants were recovered, clarified by centrifugation and titrated by TCID50. (**B**) HeLa cells were infected with a recombinant strain of MV expressing EGFP (MOI = 2), and incubated for 48 hours in the presence of DD264 at 40 µM or DMSO alone. (**C**) Same experiment was performed with MRC5 cells. Scale bar = 200 µm. (**D**) HEK-293T cells were infected with CVB3 (MOI = 0.1), and incubated with DMSO alone or DD264 at 40 µM. After 24 hours, cell cultures were harvested after freezing and thawing of the plates. Supernatants were recovered, clarified by centrifugation and titrated by TCID50. Experiment was performed in duplicate, and data represent means ± SD.
(PDF)

Figure S4 **Effects of blocking antibodies against IFN-α/β on DD264-mediated amplification of cellular response to ssRNA and antiviral activity.** (**A**) HEK-293 cells with the ISRE-luciferase reporter gene (STING-37 cells) were transfected with increasing doses of synthetic 5′-triphosphate RNA molecules (ssRNA), and incubated in the presence of DD264 (80 µM) or DMSO alone in 96-well cultures plates. Culture medium was supplemented or not with a cocktail of sheep polyclonal antibodies against IFN-α and β at 2000 and 500 IU/ml, respectively. Such concentrations were sufficient to totally block ISRE-luciferase induction by 1000 IU/ml of recombinant IFN-α or β (data not shown). After 24 hours, luciferase expression was determined. Experiment was performed in duplicate, and data represent means ± SD. (**B**) HEK-293T cells were infected with a recombinant strain of MV expressing EGFP (MOI = 0.1), and incubated for 48 hours in the presence of DD264 at 80 µM or DMSO alone. Culture medium was supplemented or not with anti-IFN-α/β antibodies as described above. Upper panel is showing EGFP expression by fluorescence microscopy whereas lower panels correspond to bright fields. Scale bar = 200 µm.
(PDF)

Figure S5 **IRF1 is required for ISG expression in MV-infected cells.** (**A–L**) HEK-293 cells with the ISRE-luciferase reporter gene (STING-37 cells) were transfected with control siRNA (CT) or siRNA directed against IRF1 and cultured for 48 hours. Cells were infected with MV (MOI = 0.1) and cultured for 24 hours. Total RNAs were extracted, and expression levels of indicated genes were quantified by qRT-PCR. Data were normalized relative to control housekeeping genes (GAPDH, HPRT1, and 18S).
(PDF)

Figure S6 **Antiviral activity of DD264 and brequinar is abrogated when silencing IRF1 expression HeLa cells.** (**A–B**) HeLa cells were transfected with control siRNA (solid colors) or siRNA directed against IRF1 (shaded colors) and cultured for 48 hours. Then, cells were infected with recombinant MV strain expressing luciferase (MOI = 0.1) or CHIKV strain expressing luciferase (MOI = 0.2), and incubated with increasing concentrations of DD264 or DMSO alone. After 24 hours, luciferase expression was determined. Experiment was performed in triplicate, and data represent means ± SD. (**C–D**) Same experiment was performed but cells were treated with increasing concentrations of brequinar instead of DD264. * corresponds to p-values<0.05.
(PDF)

Figure S7 **Validation of IRF1 silencing.** (**A**) HEK-293T or (**B**) HeLa cells were transfected with control siRNA (CT) or siRNA directed against *IRF1* and cultured for 48 hours. *IRF1* silencing was confirmed by western-blot analysis of IRF1 expression levels.
(PDF)

Figure S8 **IRF1 expression level and localization pattern.** (**A**) HEK-293T cells were cultured for 8 or 24 hours with DMSO alone, increasing doses of DD264 or Brequinar, or IFN-γ (100 ng/ml). IRF1 expression levels were determined by western-blot. IRF1 is induced by IFN-γ at 8 hours but expression level is back to normal at 24 hours. (**B**) HeLa cells were cultured for 24 hours with DMSO, DD264 or Brequinar. Subcellular localization of IRF1 was determined by immunostaining and fluorescence microscopy. Cell nuclei were stained with DAPI. Scale bar = 40 µm.
(PDF)

Figure S9 **DD264 does not inhibit herpesvirus replication.** MRC5 cells were infected with HSV1 or HSV2 (MOI = 1), and then cultured with DD264 at 80 µM or DMSO alone (None). After 24 h, herpesvirus antigens were detected by immunostaining and fluorescence microscopy. Scale bar = 200 µm.
(PDF)

Figure S10 **Best conformational docking of DD264 in the hydrophobic tunnel of DHODH where brequinar, A77-1726 and probably ubiquinone bind (pdb id:1D3G).** (**A**) DHODH is shown as a grey ribbon and DD264 is represented as sticks. (**B**) Higher magnification of the tunnel with amino-acid residues invoved in DD264 binding. Structural images were generated using PyMOL (www.pymol.org). (**C**) Data table of energy binding parameters for brequinar, A77-1726 and DD264.
(PDF)

Table S1 **Induction of IFN-α, β, γ and IRF-1 genes in DD264-stimulated cells.** HEK-293T cells were incubated with DMSO or DD264 (80 µM) for 24 hours. Total RNA were extracted and gene expression was determined by qRT-PCR. Experiment was performed three times and data represent mean values.
(PDF)

Table S2 **Expression of IFN-α, β, and γ in DD264-stimulated cells.** HEK-293T cells were incubated with DMSO or DD264 (80 µM) for 24 hours. Cytokine expression levels were determined by ELISA.
(PDF)

Table S3 **Chemical structures of DD264 analogs and corresponding potency to inhibit MV replication as described in** **Figure 2B** **.** Antiviral activity was scored as followed: “−” (IC50>80 µM),“+/−” (IC50 = 40–80 µM), “+” (IC50 = 20–40 µM), “++” (IC50 = 10–20 µM), and “+++” (IC50 = 2–10 µM).
(PDF)

Protocol S1 **Synthetic Experimental Procedures and Characterization.**
(DOC)
