# Supplementary figures and images for: Inhibition of Pyrimidine Biosynthesis Pathway Suppresses Viral Growth through Innate Immunity
Source: PLoS Pathog. 2013 Oct 3;9(10):e1003678. doi: 10.1371/journal.ppat.1003678 (PMC3789760; doi:10.1371/journal.ppat.1003678)

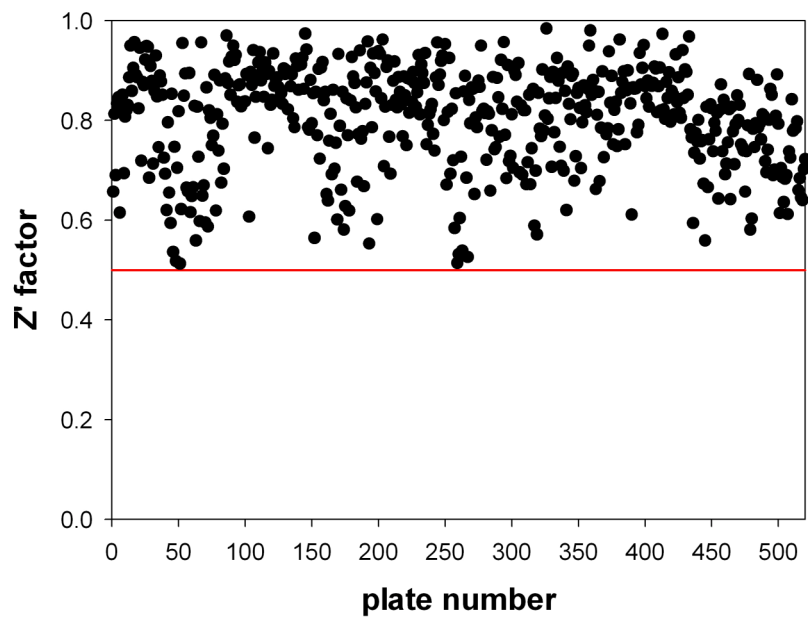

Supplement: Figure S1 — Evaluation of the screen using the Z′-factor value plotted for each plate. (PDF) [file ppat.1003678.s001.pdf]

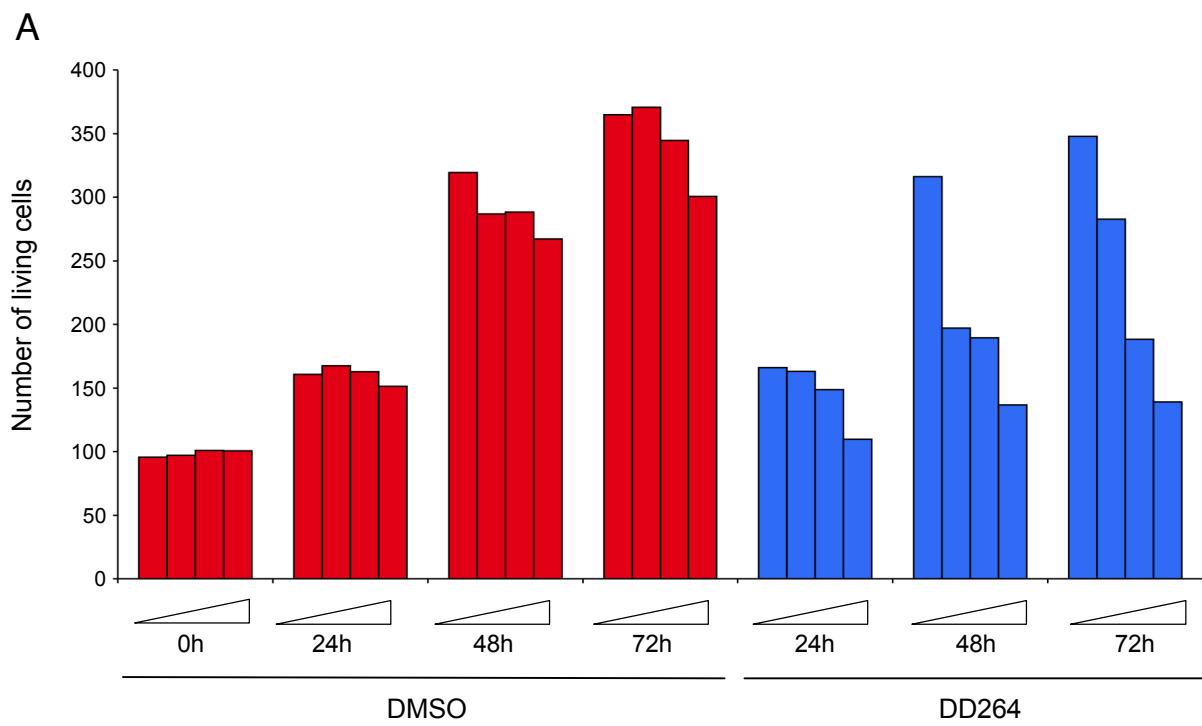

**B**

|                | DMSO           | DD264 (80 $\mu$ M) |
|----------------|----------------|--------------------|
| TUNEL+ (%)     | 0.3 $\pm$ 0.2  | 1.7 $\pm$ 0.9      |
| G2/M phase (%) | 22.5 $\pm$ 1.0 | 4.6 $\pm$ 0.9      |

Supplement: Figure S2 — DD264 inhibits the proliferation of HEK-293T cells. (A) HEK-293T cells were incubated with increasing doses of DD264 (10, 20, 40 or 80 µM) or matching volumes of DMSO alone. After 0, 24, 48 and 72 hours of culture, the number of living cells was determined using CellTiter-GLO reagent (Promega). This luciferase-based assay evaluates by ATP quantification the number of metabolically active cells in culture wells. The number of living cells is expressed as a percentage relative to the initial number of living cells at t = 0 hours. (B) HEK-293T cells were incubated with DD264 at 80 µM or DMSO alone. After 24 h, cells were stained for DNA fragmentation by TUNEL (Terminal deoxynucleotidyl transferase dUTP nick end labeling). Alternatively, cells were stained with propidium iodide for cell cycle analysis by flow cytometry, and percentage of cells in G2/M phase is indicated. (PDF) [file ppat.1003678.s002.pdf]

A

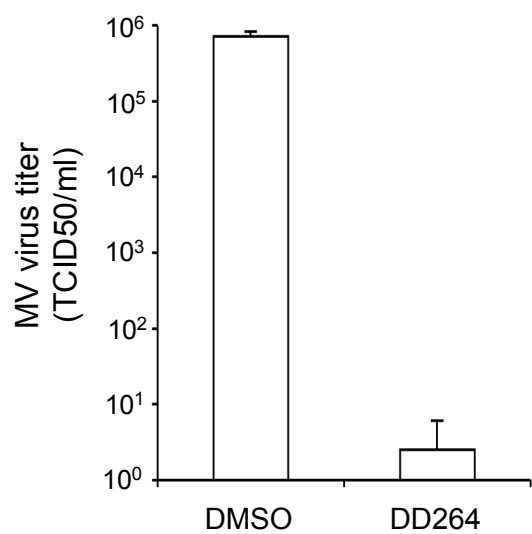

B

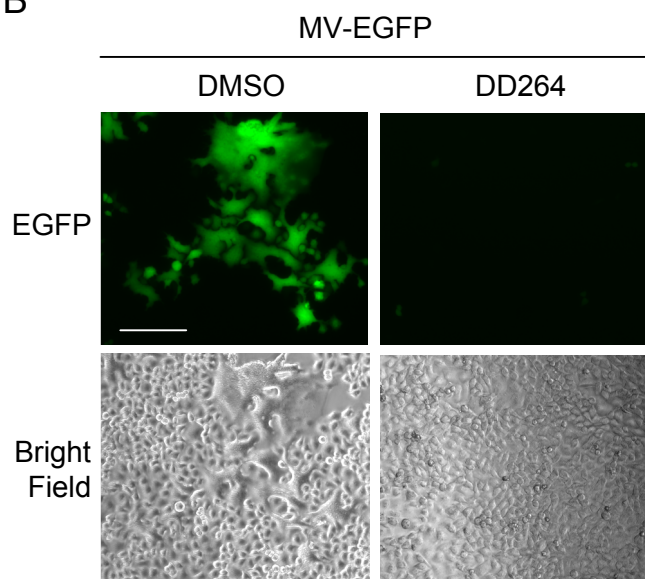

C

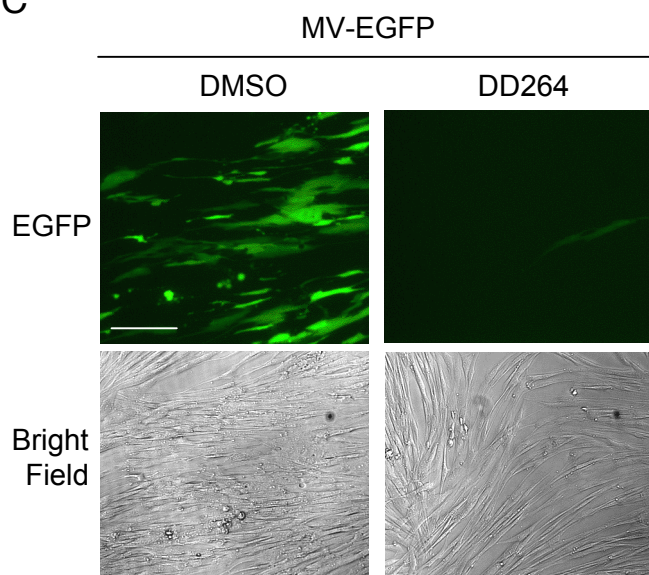

D

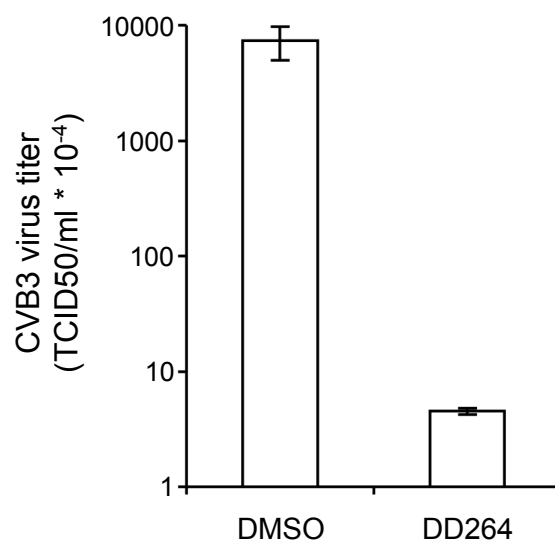

Supplement: Figure S3 — DD264 inhibits MV and CVB3 growth. (A) HEK-293T cells were infected with MV (MOI = 0.2) and then cultured for 48 hours with DMSO or DD264 at 80 µM. Cell cultures were harvested by scraping, and then frozen and thawed to release viral particles. Supernatants were recovered, clarified by centrifugation and titrated by TCID50. (B) HeLa cells were infected with a recombinant strain of MV expressing EGFP (MOI = 2), and incubated for 48 hours in the presence of DD264 at 40 µM or DMSO alone. (C) Same experiment was performed with MRC5 cells. Scale bar = 200 µm. (D) HEK-293T cells were infected with CVB3 (MOI = 0.1), and incubated with DMSO alone or DD264 at 40 µM. After 24 hours, cell cultures were harvested after freezing and thawing of the plates. Supernatants were recovered, clarified by centrifugation and titrated by TCID50. Experiment was performed in duplicate, and data represent means ± SD. (PDF) [file ppat.1003678.s003.pdf]

A

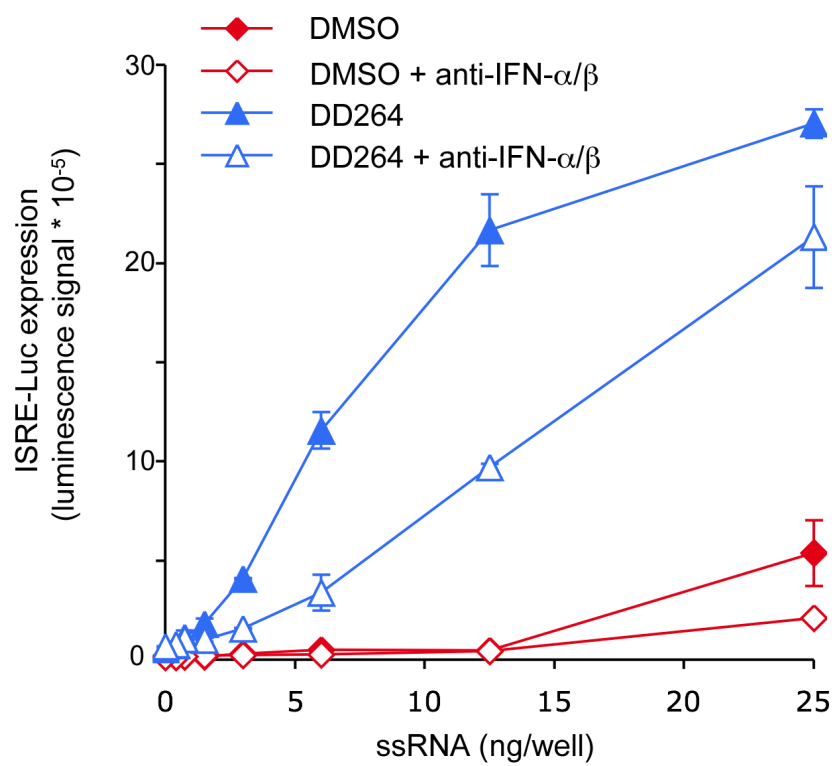

B

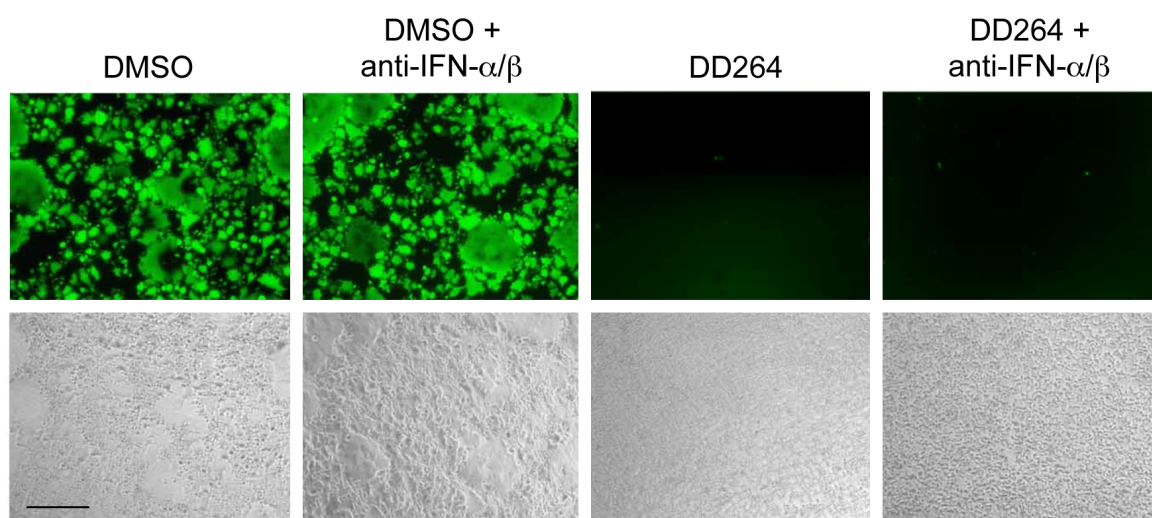

Supplement: Figure S4 — Effects of blocking antibodies against IFN-α/β on DD264-mediated amplification of cellular response to ssRNA and antiviral activity. (A) HEK-293 cells with the ISRE-luciferase reporter gene (STING-37 cells) were transfected with increasing doses of synthetic 5′-triphosphate RNA molecules (ssRNA), and incubated in the presence of DD264 (80 µM) or DMSO alone in 96-well cultures plates. Culture medium was supplemented or not with a cocktail of sheep polyclonal antibodies against IFN-α and β at 2000 and 500 IU/ml, respectively. Such concentrations were sufficient to totally block ISRE-luciferase induction by 1000 IU/ml of recombinant IFN-α or β (data not shown). After 24 hours, luciferase expression was determined. Experiment was performed in duplicate, and data represent means ± SD. (B) HEK-293T cells were infected with a recombinant strain of MV expressing EGFP (MOI = 0.1), and incubated for 48 hours in the presence of DD264 at 80 µM or DMSO alone. Culture medium was supplemented or not with anti-IFN-α/β antibodies as described above. Upper panel is showing EGFP expression by fluorescence microscopy whereas lower panels correspond to bright fields. Scale bar = 200 µm. (PDF) [file ppat.1003678.s004.pdf]

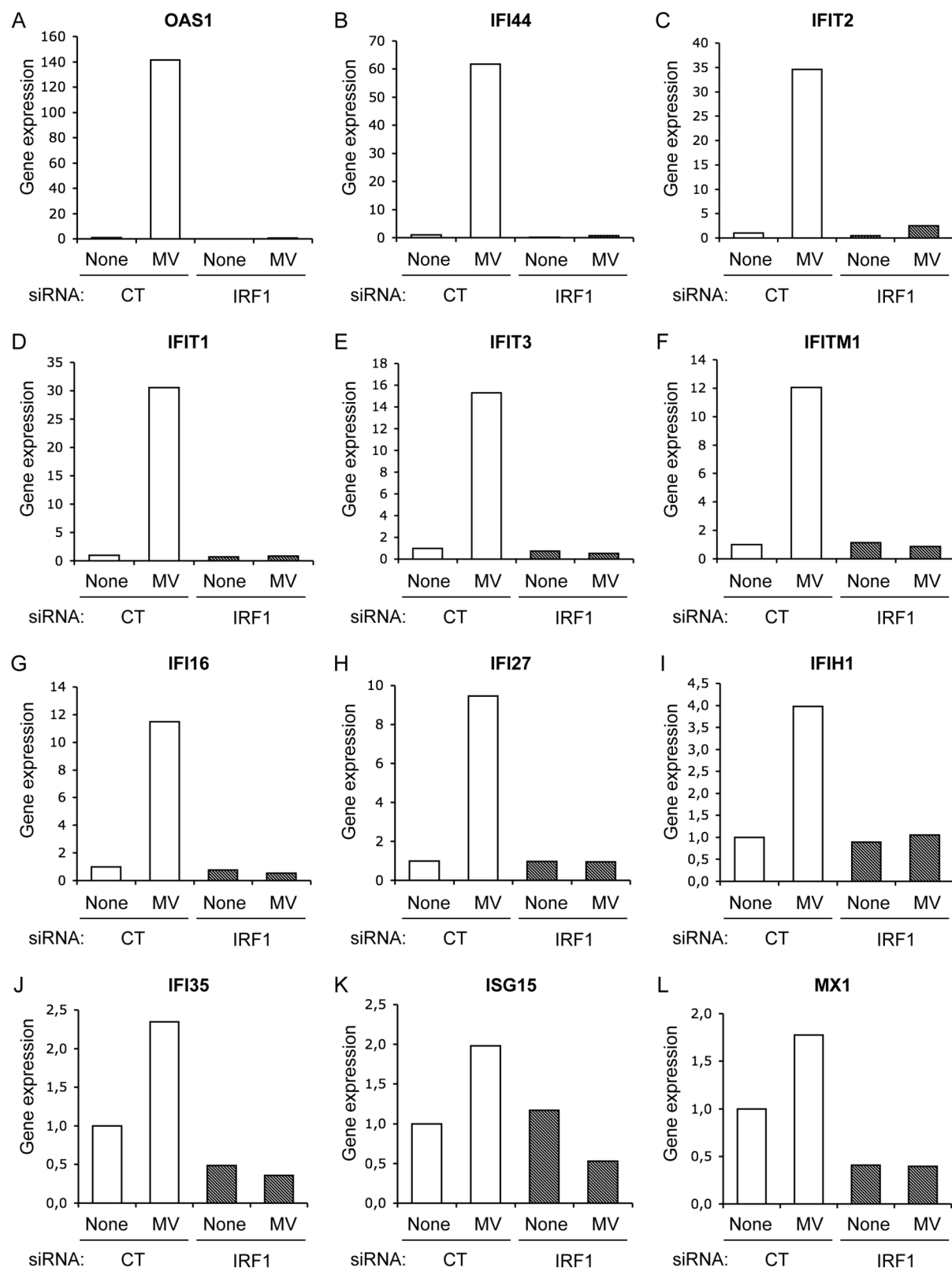

Supplement: Figure S5 — IRF1 is required for ISG expression in MV-infected cells. (A–L) HEK-293 cells with the ISRE-luciferase reporter gene (STING-37 cells) were transfected with control siRNA (CT) or siRNA directed against IRF1 and cultured for 48 hours. Cells were infected with MV (MOI = 0.1) and cultured for 24 hours. Total RNAs were extracted, and expression levels of indicated genes were quantified by qRT-PCR. Data were normalized relative to control housekeeping genes (GAPDH, HPRT1, and 18S). (PDF) [file ppat.1003678.s005.pdf]

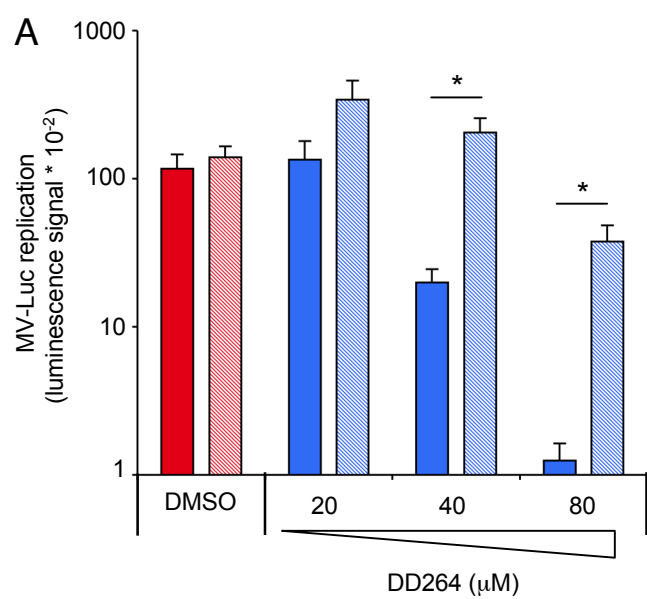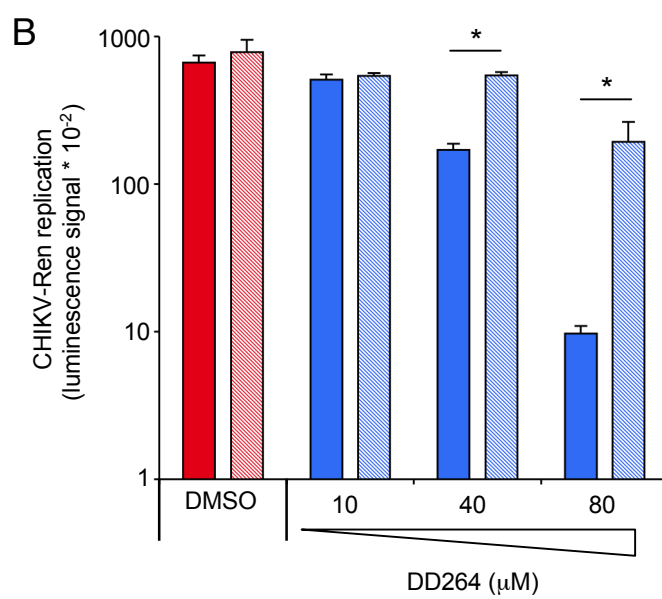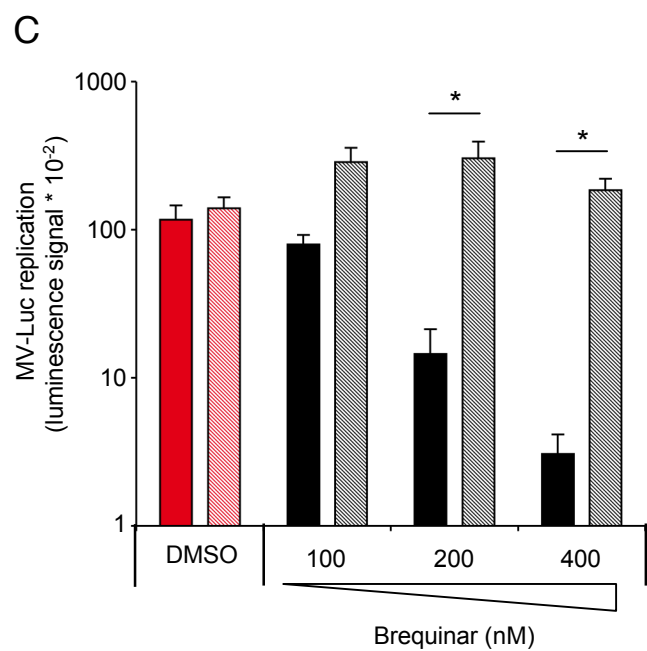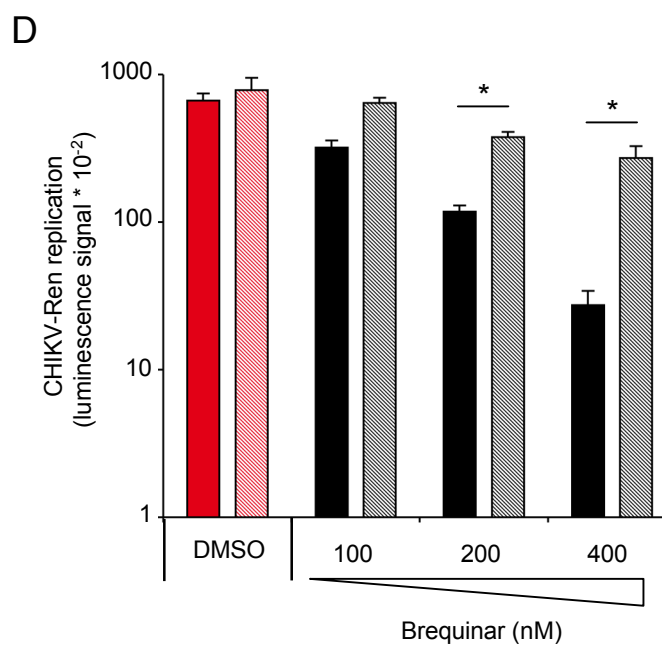

Supplement: Figure S6 — Antiviral activity of DD264 and brequinar is abrogated when silencing IRF1 expression HeLa cells. (A–B) HeLa cells were transfected with control siRNA (solid colors) or siRNA directed against IRF1 (shaded colors) and cultured for 48 hours. Then, cells were infected with recombinant MV strain expressing luciferase (MOI = 0.1) or CHIKV strain expressing luciferase (MOI = 0.2), and incubated with increasing concentrations of DD264 or DMSO alone. After 24 hours, luciferase expression was determined. Experiment was performed in triplicate, and data represent means ± SD. (C–D) Same experiment was performed but cells were treated with increasing concentrations of brequinar instead of DD264. * corresponds to p-values<0.05. (PDF) [file ppat.1003678.s006.pdf]

A

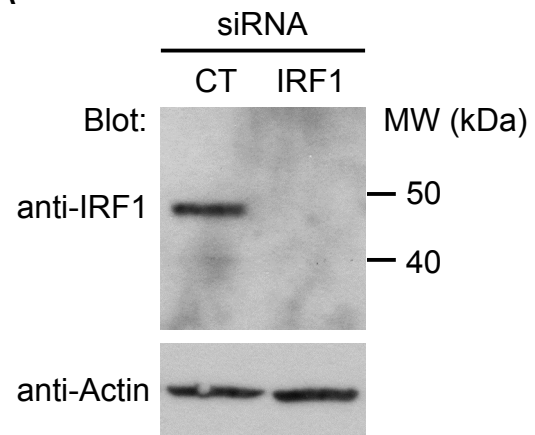

B

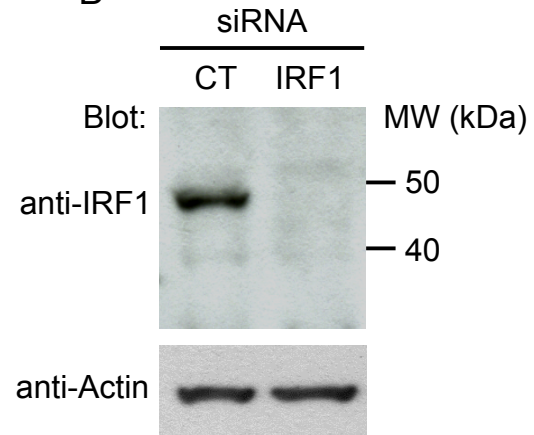

Supplement: Figure S7 — Validation of IRF1 silencing. (A) HEK-293T or (B) HeLa cells were transfected with control siRNA (CT) or siRNA directed against IRF1 and cultured for 48 hours. IRF1 silencing was confirmed by western-blot analysis of IRF1 expression levels. (PDF) [file ppat.1003678.s007.pdf]

A

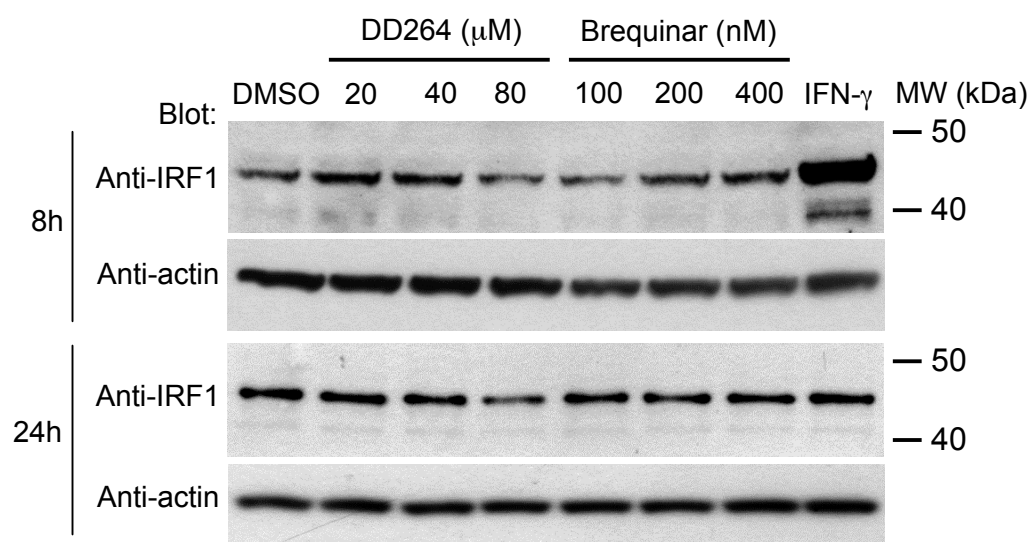

B

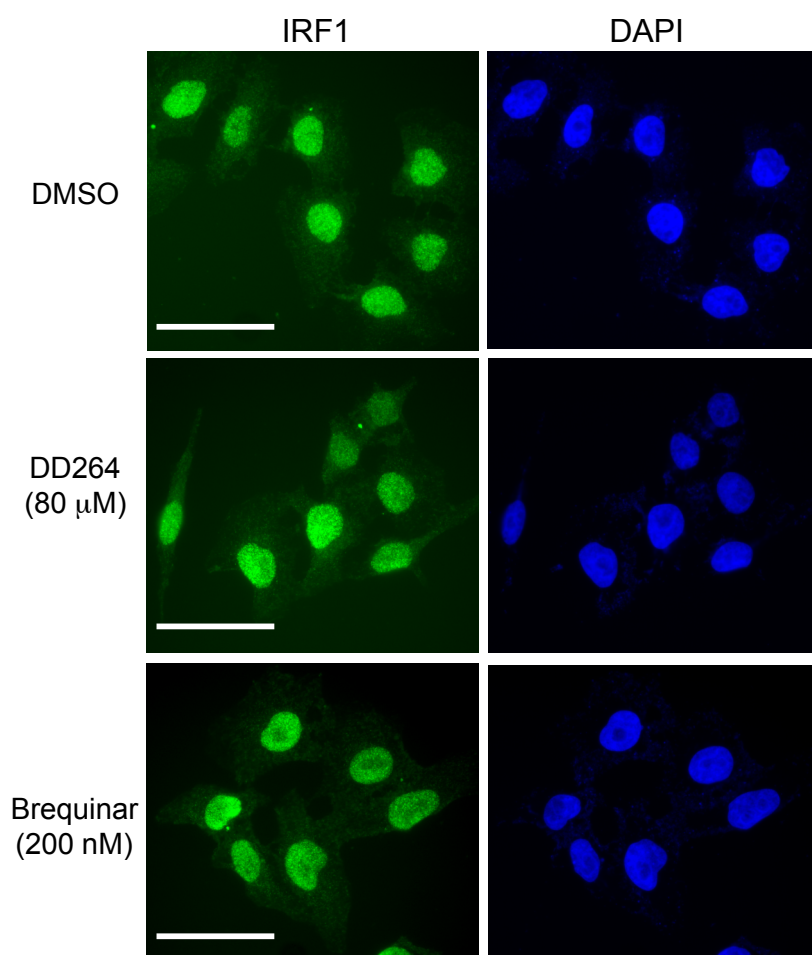

Supplement: Figure S8 — IRF1 expression level and localization pattern. (A) HEK-293T cells were cultured for 8 or 24 hours with DMSO alone, increasing doses of DD264 or Brequinar, or IFN-γ (100 ng/ml). IRF1 expression levels were determined by western-blot. IRF1 is induced by IFN-γ at 8 hours but expression level is back to normal at 24 hours. (B) HeLa cells were cultured for 24 hours with DMSO, DD264 or Brequinar. Subcellular localization of IRF1 was determined by immunostaining and fluorescence microscopy. Cell nuclei were stained with DAPI. Scale bar = 40 µm. (PDF) [file ppat.1003678.s008.pdf]

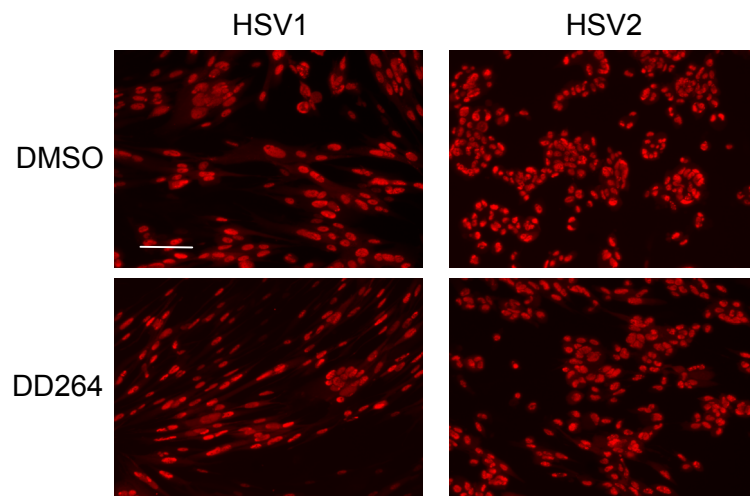

Supplement: Figure S9 — DD264 does not inhibit herpesvirus replication. MRC5 cells were infected with HSV1 or HSV2 (MOI = 1), and then cultured with DD264 at 80 µM or DMSO alone (None). After 24 h, herpesvirus antigens were detected by immunostaining and fluorescence microscopy. Scale bar = 200 µm. (PDF) [file ppat.1003678.s009.pdf]

A

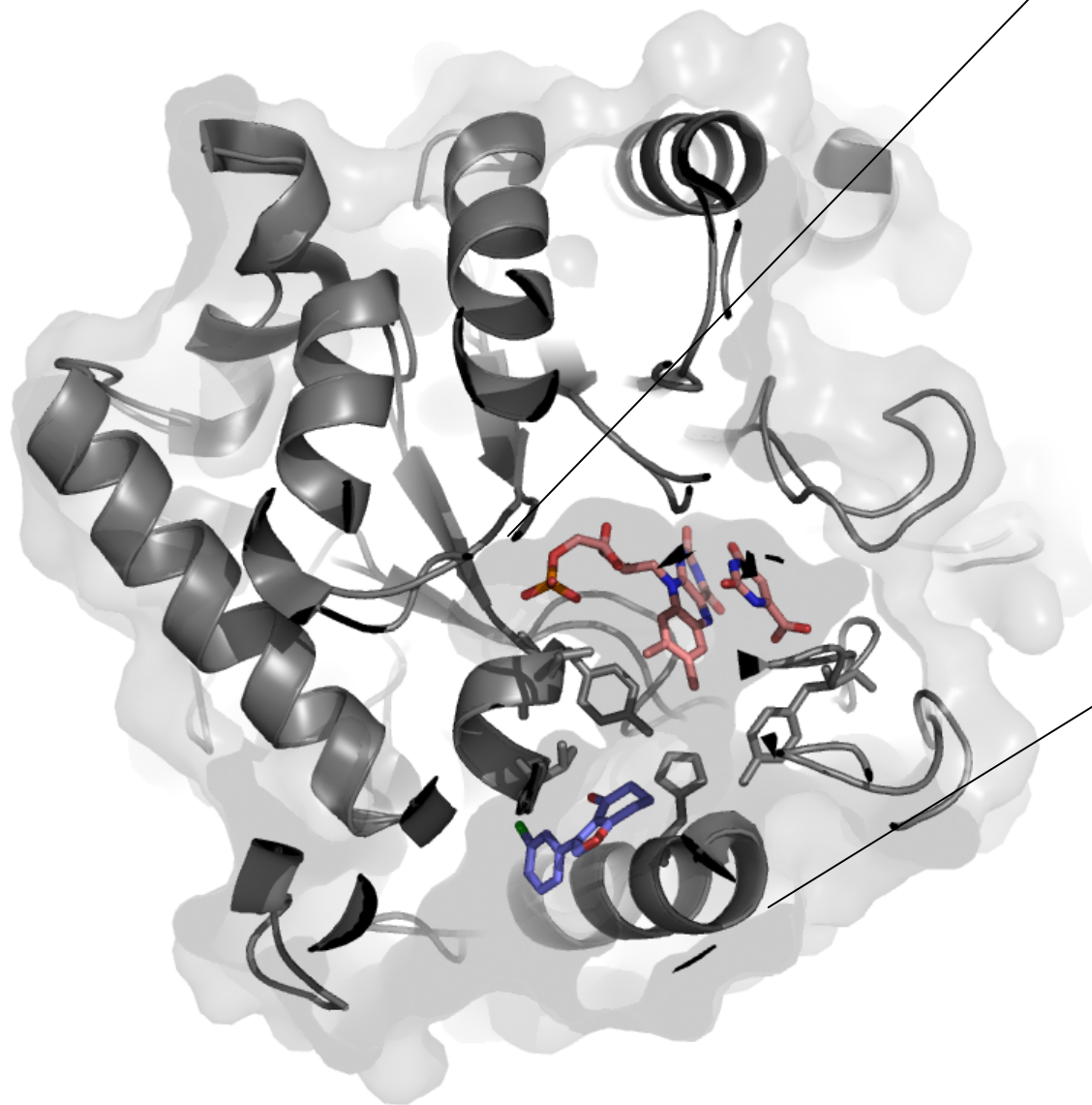

B

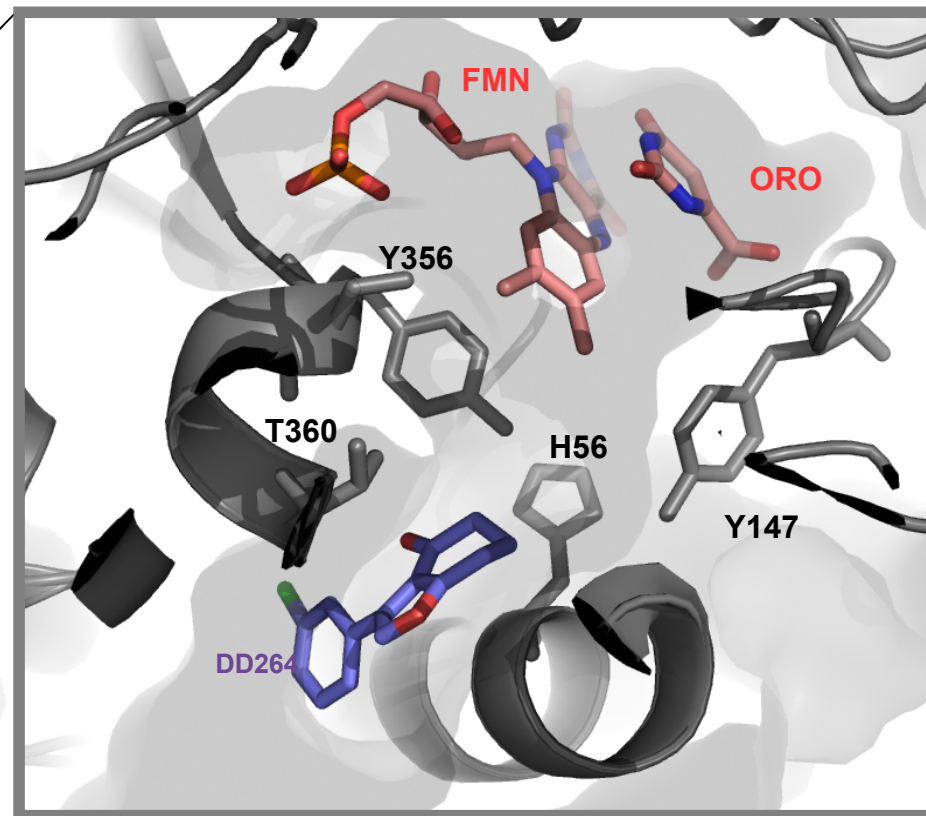

C

|           | Potential energy (kcal/mol) | Interaction energy (kcal/mol) |
|-----------|-----------------------------|-------------------------------|
| Brequinar | -35780                      | -150                          |
| A77-1726  | -37015                      | -58                           |
| DD264     | -35840                      | -101                          |

Supplement: Figure S10 — Best conformational docking of DD264 in the hydrophobic tunnel of DHODH where brequinar, A77-1726 and probably ubiquinone bind (pdb id:1D3G). (A) DHODH is shown as a grey ribbon and DD264 is represented as sticks. (B) Higher magnification of the tunnel with amino-acid residues invoved in DD264 binding. Structural images were generated using PyMOL (www.pymol.org). (C) Data table of energy binding parameters for brequinar, A77-1726 and DD264. (PDF) [file ppat.1003678.s010.pdf]
