# Supplementary material for: Inhibition of Pyrimidine Biosynthesis Pathway Suppresses Viral Growth through Innate Immunity
Source: PLoS Pathog. 2013 Oct 3;9(10):e1003678. doi: 10.1371/journal.ppat.1003678 (PMC3789760; doi:10.1371/journal.ppat.1003678)
Supplement: Table S1 — Induction of IFN-α, β, γ and IRF-1 genes in DD264-stimulated cells. HEK-293T cells were incubated with DMSO or DD264 (80 µM) for 24 hours. Total RNA were extracted and gene expression was determined by qRT-PCR. Experiment was performed three times and data represent mean values. (PDF) [file ppat.1003678.s011.pdf]

Table S1: Induction of IFN-α, β, γ and IRF-1 genes in DD264-stimulated cells.

Gene Symbol	Induction fold
IFN-α1	0.67 +/- 0.07
IFN-α14	ND
IFN-α2	ND
IFN-α21	ND
IFN-α4	ND
IFN-α5	0.93 +/- 0.26
IFN-α6	1.79 +/- 1.26
IFN-α8	ND
IFN-β1	1.01 +/- 0.41
IFN-γ	ND
IRF-1	2.2 +/- 0.92

* ND: not detectable (CT values > 35)
