# Supplementary material for: Inhibition of Pyrimidine Biosynthesis Pathway Suppresses Viral Growth through Innate Immunity
Source: PLoS Pathog. 2013 Oct 3;9(10):e1003678. doi: 10.1371/journal.ppat.1003678 (PMC3789760; doi:10.1371/journal.ppat.1003678)
Supplement: Table S2 — Expression of IFN-α, β, and γ in DD264-stimulated cells. HEK-293T cells were incubated with DMSO or DD264 (80 µM) for 24 hours. Cytokine expression levels were determined by ELISA. (PDF) [file ppat.1003678.s012.pdf]

**Table S2: Expression of IFN-α, β, and γ in DD264-stimulated cells.**

| | DMSO | DD264 | Detection threshold |
|---|---|---|---|
| IFN-α | < 2.5 IU/ml | < 2.5 IU/ml | 2.5 IU/ml |
| IFN-β | < 5 IU/ml | < 5 IU/ml | 5 IU/ml |
| IFN-γ | < 0.09 IU/ml | < 0.09 IU/ml | 0.09 IU/ml |
