# Supplementary material for: Inhibition of Pyrimidine Biosynthesis Pathway Suppresses Viral Growth through Innate Immunity
Source: PLoS Pathog. 2013 Oct 3;9(10):e1003678. doi: 10.1371/journal.ppat.1003678 (PMC3789760; doi:10.1371/journal.ppat.1003678)
Supplement: Table S3 — Chemical structures of DD264 analogs and corresponding potency to inhibit MV replication as described in Figure 2B . Antiviral activity was scored as followed: “−” (IC50>80 µM),“+/−” (IC50 = 40–80 µM), “+” (IC50 = 20–40 µM), “++” (IC50 = 10–20 µM), and “+++” (IC50 = 2–10 µM). (PDF) [file ppat.1003678.s013.pdf]

Table S3. Chemical structures of DD264 analogs and corresponding potency to inhibit MV replication as described in Figure 2B. Antiviral activity was scored as followed: "-" (IC50 > 80 μM),"+/-" (IC50 = 40-80 μM), "+" (IC50 = 20-40 μM), "++" (IC50 = 10-20 μM), and "+++" (IC50 = 2-10 μM).

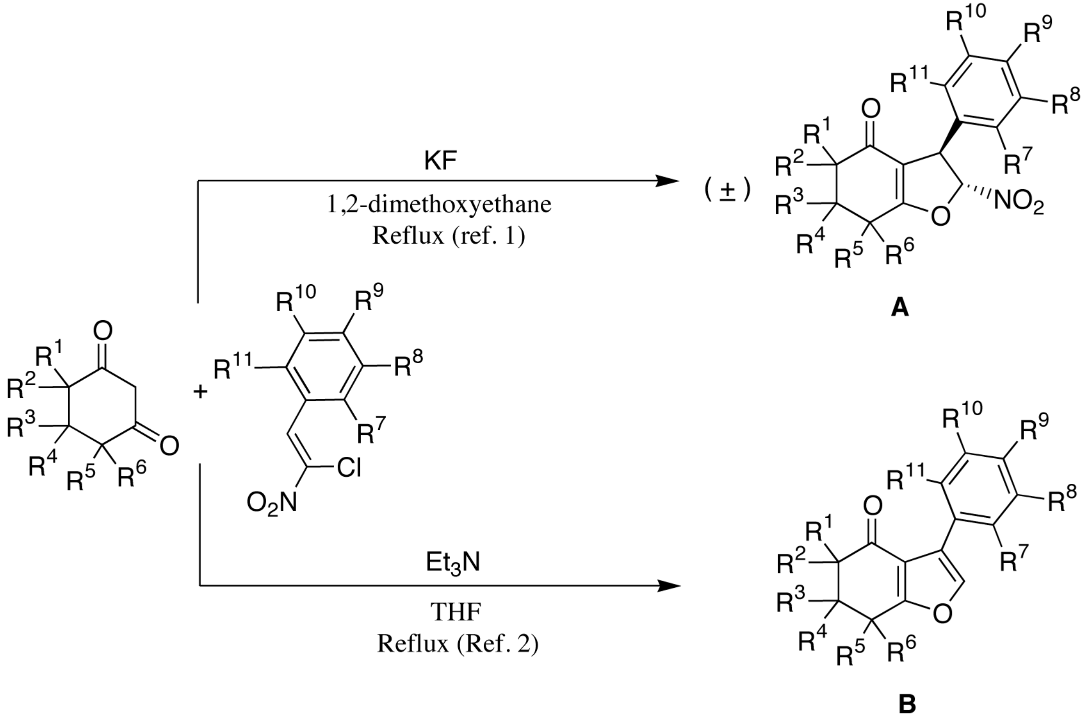

		R^1	R^2	R^3	R^4	R^5	R^6	R^7	R^8	R^9	R^{10}	R^{11}	IC50
**A**	**DD243**	H	H	CH_3	CH_3	H	H	H	H	H	H	H	-
**A**	**DD244**	H	H	CH_3	CH_3	H	H	Cl	H	H	H	H	+/-
**A**	**DD245**	H	H	CH_3	CH_3	H	H	H	Cl	H	H	H	+/-
**A**	**DD246**	H	H	CH_3	CH_3	H	H	H	H	Cl	H	H	+/-
**A**	**DD707**	H	H	H	H	H	H	Cl	H	H	H	H	-
**A**	**GAC33**	H	H	H	H	H	H	H	Cl	H	H	H	-
**A**	**DD698**	H	H	H	H	H	H	H	Cl	H	H	OCH_3	-

**B**	**GAC27**	H	H	H	H	H	H	H	H	H	H	H	-
**B**	**DD706**	H	H	H	H	H	H	Cl	H	H	H	H	+/-
**B**	**DD264**	H	H	H	H	H	H	H	Cl	H	H	H	++
**B**	**DD703**	H	H	H	H	H	H	H	H	Cl	H	H	+/-
**B**	**GAC20**	H	H	H	H	H	H	Cl	Cl	H	H	H	+
**B**	**GAC38**	H	H	H	H	H	H	Cl	H	H	Cl	H	+/-
**B**	**GAC15**	H	H	H	H	H	H	H	Cl	Cl	H	H	+
**B**	**DD711**	H	H	H	H	H	H	H	Cl	H	Cl	H	+/-
**B**	**DD777**	H	H	H	H	H	H	Cl	H	H	H	Cl	-
**B**	**DD697**	H	H	H	H	H	H	H	Cl	H	H	OCH_3	+/-
**B**	**DD277**	H	H	H	H	H	H	H	Cl	H	H	NO_2	+/-
**B**	**DD701**	H	H	H	H	H	H	H	F	H	H	H	+/-
**B**	**DD700**	H	H	H	H	H	H	H	Br	H	H	H	++
**B**	**GAC25**	H	H	H	H	H	H	H	I	H	H	H	++
**B**	**GAC18**	H	H	H	H	H	H	H	I	OCH_3	H	H	-
**B**	**GAC11**	H	H	H	H	H	H	H	CF_3	H	H	H	+/-
**B**	**GAC22**	H	H	H	H	H	H	H	CH_3	H	H	H	+
**B**	**GAC35**	H	H	H	H	H	H	H	NH_2	H	H	H	-
**B**	**DD718**	H	H	H	H	H	H	H	NO_2	H	H	H	+/-
**B**	**DD762**	H	H	H	H	H	H	H	CN	H	H	H	+/-
**B**	**GAC50**	H	H	H	H	H	H	H	$-CH=CH_2$	H	H	H	++
**B**	**DD710**	H	H	H	H	H	H	H	OCH_3	H	H	H	-
**B**	**GAC14**	H	H	H	H	H	H	H	OCH_3	OCH_3	OCH_3	H	-
**B**	**GAC19**	H	H	H	H	H	H	H	$-O-CH_2-O-$		H	H	+/-
**B**	**GAC45**	H	H	H	H	H	H	H	-CH=CH-CH=CH-		H	H	Toxic
**B**	**JP45**	H	H	H	H	H	H	H	OH	H	H	H	-
**B**	**GAC13**	H	H	H	H	H	H	H	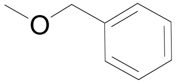	H	H	H	+/-
**B**	**GAC46**	H	H	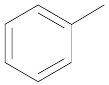	H	H	H	H	Cl	H	H	H	+
**B**	**GAC36**	H	H	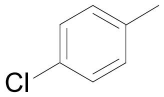	H	H	H	H	Cl	H	H	H	+/-

**B**	**GAC37**	H	H	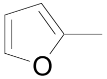	H	H	H	H	Cl	H	H	H	+
**B**	**DD694**	H	H	CH_3	CH_3	H	H	H	Cl	H	H	H	-
**B**	**DD720**	H	H	CH3	H	H	H	H	Cl	H	H	H	-
**B**	**DD772**	CH_3	CH_3	H	H	H	H	H	Cl	H	H	H	+
**B**	**JP14**	H	H	H	H	CH_3	CH_3	H	Cl	H	H	H	+/-
**B**	**DD287**	H	H	CH_3	CH_3	H	H	NO_2	H	H	H	H	Toxic
**B**	**JP20**	H	H	H	H	H	H		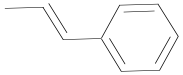	H	H	H	+
**B**	**JP17**	H	H	H	H	H	H	H	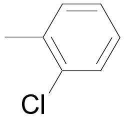	H	H	H	+
**B**	**JP13**	H	H	H	H	H	H	H	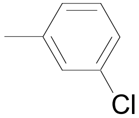	H	H	H	++
**B**	**JP11**	H	H	H	H	H	H	H	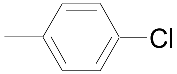	H	H	H	+
**B**	**JP33**	H	H	H	H	H	H	H	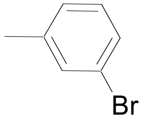	H	H	H	++
**B**	**JP15**	H	H	H	H	H	H	H	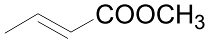	H	H	H	+
**B**	**JP23**	H	H	H	H	H	H	H	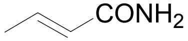	H	H	H	-
**B**	**JP27**	H	H	H	H	H	H	H	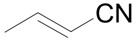	H	H	H	+/-
**B**	**JP30**	H	H	H	H	H	H	H	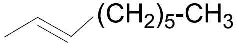	H	H	H	+
**B**	**JP4**	H	H	H	H	H	H	H	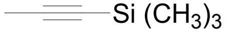	H	H	H	++
**B**	**JP6**	H	H	H	H	H	H	H	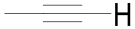	H	H	H	++
**B**	**JP8**	H	H	H	H	H	H	H	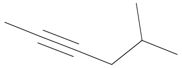	H	H	H	+
**B**	**JP54**	H	H	H	H	H	H	H	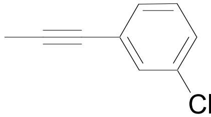	H	H	H	+
**B**	**JP50**	$CH_2CO_2C_2H_5$	H	H	H	H	H	H	Br	H	H	H	+/-

**B**	**JP29**	CH_2CO_2H	H	H	H	H	H	H	Br	H	H	H	-
**B**	**JP61f2**	Br	H	H	H	H	H	H	Cl	H	H	H	+++
**B**	**JP67**	$CH_2N(CH_3)_2$	H	H	H	H	H	H	Cl	H	H	H	Toxic
	**GAC21**												+/-
	**GAC30**												+/-
	**DD765**												-
	**GAC12**												Toxic
	**DD817**												++
	**DD747**												-
	**JP37**												-
	**JP61f1**	See below for structures											+
	**DD789**												-
	**JP55**												+/-
	**DD829**												++
	**DD297**												-

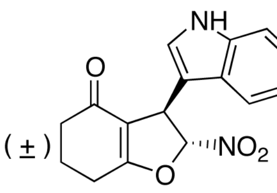

GAC21

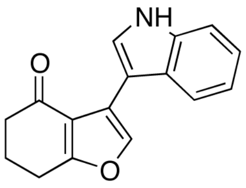

GAC30

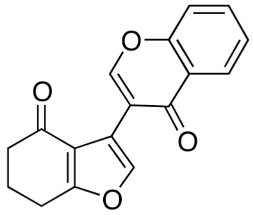

DD765

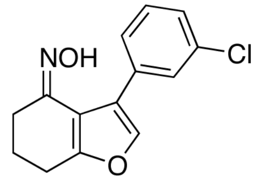

GAC12

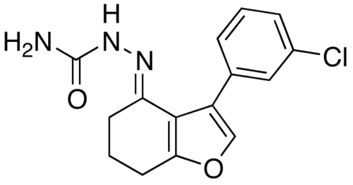

DD817

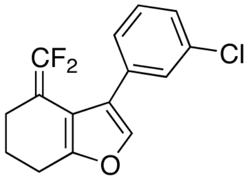

DD747

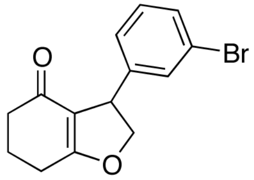

JP37

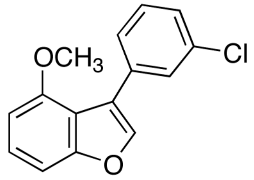

JP61f1

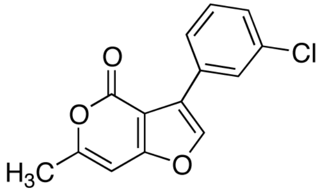

DD789

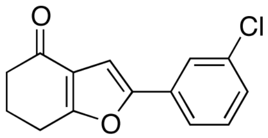

JP55

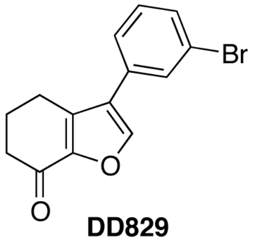

DD829

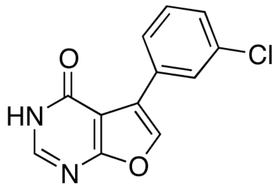

DD297
