# Supplementary material for: Inhibition of Pyrimidine Biosynthesis Pathway Suppresses Viral Growth through Innate Immunity
Source: PLoS Pathog. 2013 Oct 3;9(10):e1003678. doi: 10.1371/journal.ppat.1003678 (PMC3789760; doi:10.1371/journal.ppat.1003678)
Supplement: Protocol S1 — Synthetic Experimental Procedures and Characterization. (DOC) [file ppat.1003678.s014.doc]

**Protocol S1 : Synthetic Experimental Procedures and Characterization**

*Analysis:* Melting points were measured on a Köfler hot stage apparatus and are uncorrected. Infrared spectra were recorded on a Perkin-Elmer RX I spectrometer as deuteriochloroform solutions (or KBr discs) or with a Nicolet Magna 550 FTIR spectrometer fitted with a horizontal Attenuated Total Reflectance (ATR) Durascope Durasampler equipped with a diamond/KRS5 internal reflection element; a DTGS detector was used at a resolution of 4cm-1 . The ^1^H-NMR (300 MHz) were recorded on a Varian AC 300 spectrometer. Chemical shifts are expressed as parts per million downfield from tetramethylsilane. Splitting patterns have been designated as follows: s (singlet), d (doublet), dd (doublet of doublet), ddd (doublet of doublet of doublet), t (triplet), dt (doublet of triplet), q (quadruplet), quint (quintuplet), hept (heptuplet), m (multiplet), br. (broad signal). Coupling constants (*J* values) are listed in hertz (Hz). Mass spectra were obtained with a ZQ 2000 MS spectrometer applying an electrospray (ES)^+^ ionization technique.

*** * ***

- The racemic *trans* 3-aryl-6,6-dimethyl-2-nitro-3,5,6,7-tetrahydro-2*H*-benzofuran-4-one **DD-243, DD244 and DD245** have already been described by one of us [1]. The new derivatives **DD707**, **GAC33** and **DD698** were prepared for the present work on a 5 mmol scale from cyclohexane-1,3-dione and the appropriate (*Z*)-β-chloro-β-nitrostyrenes using the same methodology.

***3-(2-chlorophenyl)-2-nitro-3,5,6,7-tetrahydro-2H-benzofuran-4-one* DD 707**

Yield 87%; mp 162-163°C recrystallized from a benzene/heptane mixture.

**IR (CDCl_3_) ν_max_ (cm^-1^):** 1660, 1573, 1392, 1215

**^1^H NMR (CDCl_3_) δ (ppm):** 2.17 – 2.33 (m, 2H), 2.43 – 2.50 (m, 2H), 2.70 – 2.85 (m, 2H), 5.08 (br d, 1H, J = 2.3 Hz), 5.95 (d, 1H, J = 2.3 Hz), 6.98 (dd, 1H, J = 2.2 Hz and 7.2 Hz), 7.19 – 7.31 (m, 2H), 7.45 (dd, 1H, J = 1.9 Hz and 7.4 Hz).

**MS (*m/z*):** 316-318 [M + Na]^+^.

***3-(3-chlorophenyl)-2-nitro-3,5,6,7-tetrahydro-2H-benzofuran-4-one* GAC33**

Yield 83 %; mp 146-147 °C recrystallized from a benzene/heptane mixture.

**IR (CDCl_3_) ν_max_ (cm^-1^):** 1658, 1573, 1392, 1174

**^1^H NMR (CDCl_3_) δ (ppm):** 2.15 – 2.31 (m, 2H), 2.36 – 2.52 (m, 2H), 2.72 – 2.91 (m, 2H), 4.60 (br d, 1H, J = 2.1 Hz), 5.91 (d, 1H, J = 2.1 Hz), 7.09 – 7.17 (m, 2H), 7.28-7.33 (m, 2H).

**MS (*m/z*):** 316-318 [M + Na]^+^.

***3-(5-chloro-2-methoxyphenyl)-2-nitro-3,5,6,7-tetrahydro-2H-benzofuran-4-one* DD 698**

Yield 76%; mp 171-172°C recrystallized from a benzene/heptane mixture.

**IR (CDCl_3_) ν_max_ (cm^-1^):** 1654, 1571, 1394, 1216

**^1^H NMR (CDCl_3_) δ (ppm):** 2.17 – 2.31 (m, 2H), 2.38 – 2.58 (m, 2H), 2.71 – 2.81 (m, 2H), 3.86 (s, 3H), 4.89 (br s, 1H), 5.88 (d, 1H, J = 2.5 Hz), 6.83 (d, 1H, J = 2.4 Hz), 6.84 (d, 1H, J = 8.7 Hz), 7.25 (dd, 1H, J = 2.4 Hz and 8.7 Hz).

**MS (*m/z*):** 346-348 [M + Na]^+^.

- The 3-aryl-6,7-dihydro-5*H*-benzofuran-4-ones **GAC27**, **DD706**, **DD264, DD703**, **GAC20, GAC38, GAC15**, **DD711**, **DD777,** **DD697**, **DD277**, **DD289**, **DD701**, **DD700**, **GAC25, GAC18, GAC11**, **GAC22**, **GAC35**, **DD718**, **DD762, GAC50,** **DD710**, **GAC14**, **GAC19, GAC45, JP45,** **GAC13**, were prepared on a 5 mmol scale from cyclohexane-1,3-dione and the appropriate (*Z*)-β-chloro-β-nitrostyrenes according to a procedure previously published by one of us [2]. The syntheses of the 6-substituted derivatives **GAC46, GAC36**, **GAC37**, **DD694,** **DD720** were performed starting from (*Z*)-1-Chloro-3-(2-chloro-2-nitroethenyl)benzene and racemic 5-phenylcyclohexane-1,3-dione, racemic 5-(4-chlorophenyl)cyclohexane-1,3-dione, racemic 5-(2-furanyl)cyclohexane-1,3-dione, dimedone or racemic 5-methylcyclohexane-1,3-dione, respectively. Similarly, the 4,4-dimethyl- and 7,7-dimethylbenzofuran-4-ones **DD772** and **JP14** were obtained pure from (*Z*)-1-Chloro-3-(2-chloro-2-nitroethenyl)benzene and 4,4-dimethylcyclohexane-1,3-dione after column chromatography and subsequent recrystallization. The nitro derivative **DD287** was synthesized in an identical way by reaction between (*Z*)-2-(2-chloro-2-nitroethenyl)nitrobenzene and dimedone.

***3-Phenyl-6,7-dihydro-5H-benzofuran-4-one* GAC27**

Yield 78 %; mp 70-72 °C recrystallized from hexane

**IR (CDCl_3_) ν_max_ (cm^-1^):** 1674,1599, 1454, 1411

**^1^H NMR (CDCl_3_) δ (ppm):** 2.21 (quint, 2H, *J* = 6.3 Hz ), 2.55 (br t, 2H, *J* = 6.3 Hz), 2.93 (t, 2H, *J* = 6.3 Hz), 7.28 – 7.41 (m, 3H), 7.43 (s, 1H), 7.59 – 7.65 (m, 2H),.

**MS (*m/z*):** 235-237 [M + Na]^+^.

***3-(2-chlorophenyl)-6,7-dihydro-5H-benzofuran-4-one* DD706**

Yield 77%; mp 147-148°C recrystallized from heptane.

**IR (CDCl_3_) ν_max_ (cm^-1^):** 1672, 1006

**^1^H NMR (CDCl_3_) δ (ppm):** 2.21 (quint, 2H, *J* = 6.4 Hz ), 2.50 (br t, 2H, *J* = 6.4 Hz), 2.95 (t, 2H, *J* = 6.4 Hz), 7.21 – 7.31 (m, 2H), 7.32 – 7.39 (m, 1H), 7.40 (s, 1H),.

**MS (*m/z*):** 247-249 [M + H]^+^, 269-271 [M + Na]^+^.

***3-(3-chlorophenyl)-6,7-dihydro-5H-benzofuran-4-one* DD264**

Yield 72%; mp 87-88°C recrystallized from heptane.

**IR (CDCl_3_) ν_max_ (cm^-1^):** 1675, 1216

**^1^H NMR (CDCl_3_) δ (ppm):** 2.21 (quint, 2H, *J* = 6.3 Hz ), 2.55 (br t, 2H, *J* = 6.3 Hz), 2.94 (t, 2H, *J* = 6.3 Hz), 7.26 – 7.34 (m, 2H), 7.44 (s, 1H), 7.50 – 7.56 (m, 1H), 7.61 (dd, 1H, *J* = 1.0 Hz and 2.1 Hz)

**MS (*m/z*):** 247-249 [M + H]^+^, 269-271 [M + Na]^+^.

***3-(4-chlorophenyl)-6,7-dihydro-5H-benzofuran-4-one* DD703**

Yield 81%; mp 104-105°C (with allotropic change at 100-102°C) recrystallized from heptane.

**IR (CDCl_3_) ν_max_ (cm^-1^):** 1674, 1216

**^1^H NMR (CDCl_3_) δ (ppm):** 2.20 (quint, 2H, *J* = 6.4 Hz ), 2.54 (br t, 2H, *J* = 6.4 Hz), 2.93 (t, 2H, *J* = 6.4 Hz), 7.30 – 7.36 (A part of AA’BB’system, 2H, J_app_ = 8.7 Hz), 7.42 (s, 1H), 7.53 – 7.58 (B part of AA’BB’system, 2H, J_app_ = 8.7 Hz).

**MS (*m/z*):** 247-249 [M + H]^+^, 269-271 [M + Na]^+^.

***3-(2,3-dichlorophenyl)-6,7-dihydro-5H-benzofuran-4-one* GAC20**

Yield 82 %; mp 136-137 °C recrystallized from heptane

**IR (CDCl_3_) ν_max_ (cm^-1^):** 1679, 1453, 1430, 1411

**^1^H NMR (CDCl_3_) δ (ppm):** 2.22 (quint, 2H, *J* = 6.3 Hz ), 2.51 (br t, 2H, *J* = 6.3 Hz), 2.95 (t, 2H, *J* = 6.3 Hz), 7.19 (t, 1H, *J* = 7.8 Hz ), 7.25 (dd, 1H *J* = 2.1 Hz and 7.8 Hz) 7.39 (s, 1H), 7.45 (dd, 1H, *J* = 2.1 and 7.8 Hz)

**MS (*m/z*):** 303-305-307 [M + Na]^+^.

***3-(2,5-dichlorophenyl)-6,7-dihydro-5H-benzofuran-4-one* GAC38**

Yield 79 %; mp 150-151 °C recrystallized from heptane

**IR (CDCl_3_) ν_max_ (cm^-1^):** 1679, 1097

**^1^H NMR (CDCl_3_) δ (ppm):** 2.21 (quint, 2H, *J* = 6.3 Hz ), 2.51 (br t, 2H, *J* = 6.3 Hz), 2.95 (t, 2H, *J* = 6.3 Hz), 7.19 (t, 1H, *J* = 7.8 Hz ), 7.25 (dd, 1H *J* = 2.5 Hz and 8.6 Hz), 7.35 (d, 1H, *J* = 8.6 Hz), 7.36 (d, 1H, *J* = 2.5 Hz), 7.41 (s, 1H).

**MS (*m/z*):** 303-305-307 [M + Na]^+^.

***3-(3,4-dichlorophenyl)-6,7-dihydro-5H-benzofuran-4-one* GAC15**

Yield 91%; mp 122-123°C recrystallized from heptane.

**IR (CDCl_3_) ν_max_ (cm^-1^):** 1674, 1216

**^1^H NMR (CDCl_3_) δ (ppm):** 2.21 (quint, 2H, *J* = 6.3 Hz ), 2.55 (br t, 2H, *J* = 6.3 Hz), 2.94 (t, 2H, *J* = 6.3 Hz), 7.43 (d, 1H, *J* = 8.4 Hz), 7.45 (s, 1H), 7.50 (dd, 1H *J* = 2.1 Hz and 8.4 Hz), 7.73 (d,1H, J = 2.1 Hz).

**MS (*m/z*):** 303-305-307 [M + Na]^+^.

***3-(3,5-dichlorophenyl)-6,7-dihydro-5H-benzofuran-4-one* DD711**

Yield 87%; mp 105-106°C (with allotropic change at 98-100°C) recrystallized from heptane.

**IR (CDCl_3_) ν_max_ (cm^-1^):** 1676, 1216

**^1^H NMR (CDCl_3_) δ (ppm):** 2.21 (quint, 2H, *J* = 6.4 Hz ), 2.56 (br t, 2H, *J* = 6.4 Hz), 2.93 (t, 2H, *J* = 6.4 Hz), 7.29 (t, 1H, *J* = 1.9 Hz), 7.46 (s, 1H), 7.54 (d, 2H *J* = 1.9 Hz).

**MS (*m/z*):** 303-305-307 [M + Na]^+^.

***3-(2,6-dichlorophenyl)-6,7-dihydro-5H-benzofuran-4-one* DD777**

Yield 76 %; mp 139-140°C recrystallized from heptane

**IR (CDCl_3_) ν_max_ (cm^-1^):** 1678, 1558, 1429, 1096, 1006

**^1^H NMR (CDCl_3_) δ (ppm):** 2.24 (quint, 2H, *J* = 6.3 Hz ), 2.51 (br t, 2H, *J* = 6.3 Hz), 2.99 (t, 2H, *J* = 6.3 Hz), 7.20 (dd, 1H *J* = 7.3 Hz and 8.7 Hz), 7.31-7.35 (m, 2H), 7.46 (s, 1H), 7.39 and 7.40 (two s, 1H, two conformers).

**MS (*m/z*):** 281-283-285 [M + H]^+^ 303-305-307 [M + Na]^+^.

***3-( 5-chloro-2-methoxyphenyl)-6,7-dihydro-5H-benzofuran-4-one* DD697**

Yield 65%; mp 136-137°C recrystallized from a benzene/heptane mixture.

**IR (CDCl_3_) ν_max_ (cm^-1^):** 1672

**^1^H NMR (CDCl_3_) δ (ppm):** 2.20 (quint, 2H, *J* = 6.4 Hz ), 2.51 (br t, 2H, *J* = 6.4 Hz), 2.92 (t, 2H, *J* = 6.4 Hz), 3.78 (s,3H), 6.85 (d, 1H, *J* = 8.8 Hz), 7.25 (dd, 1H, *J* = 2.6 Hz and 8.8 Hz), 7.43 (d, 1H, *J* = 2.6 Hz), 7.47 (s, 1H).

**MS (*m/z*):** 277-279 [M + H]^+^, 299-301 [M + Na]^+^.

***3-(5-Chloro-2-nitrophenyl)-6,7-dihydro-5H-benzofuran-4-one* DD277**

Yield 81 %; mp 170-172 °C recrystallized from a benzene/heptane mixture.

**IR (CDCl_3_) ν_max_ (cm^-1^):** 1670, 1524, 1345, 1107, 1007

**^1^H NMR (CDCl_3_) δ (ppm):** 2.19 (quint, 2H, *J* = 6.3 Hz ), 2.45 (br t, 2H, *J* = 6.3 Hz), 2.94 (t, 2H, *J* = 6.3 Hz), 7.38 (d, 1H, *J* = 2.2 Hz), 7.44 (s, 1H), 7.47 (dd, 1H, *J* = 2.2 Hz and 8.7 Hz), 8.09 (d, 1H, *J* = 8.7 Hz).

**MS (*m/z*):** 292-294 [M + H]^+^**,** 314-316 [M + Na]^+^.

***3-(2-nitrophenyl)-6,7-dihydro-5H-benzofuran-4-one* DD289**

Yield 89 %; mp 188-190 °C recrystallized from a benzene/heptane mixture.

**IR (CDCl_3_) ν_max_ (cm^-1^):** 1664, 1523, 1341, 1060

**^1^H NMR (CDCl_3_) δ (ppm):** 2.19 (quint, 2H, *J* = 6.3 Hz ), 2.45 (br t, 2H, *J* = 6.3 Hz), 2.94 (t, 2H, *J* = 6.3 Hz), 7.39 (dd, 1H, *J* = 1.4 Hz and 8.1 Hz), 7.41 (s, 1H), 7.50 (dt, 1H, *J* = 1.5 Hz and 7.6 Hz), 7.59 (dt, 1H, *J* = 1.4 Hz and 7.5 Hz), 8.12 (dd, 1H, *J* = 1.2 Hz and 8.1 Hz).

**MS (*m/z*):** 258 [M + H]^+^**,** 280 [M + Na]^+^.

***3-( 3-fluorophenyl)-6,7-dihydro-5H-benzofuran-4-one* DD701**

Yield 78%; mp 65-66°C recrystallized from heptane.

**IR (CDCl_3_) ν_max_ (cm^-1^):** 1678, 1217

**^1^H NMR (CDCl_3_) δ (ppm):** 2.21 (quint, 2H, *J* = 6.4 Hz ), 2.56 (br t, 2H, *J* = 6.4 Hz), 2.94 (t, 2H, *J* = 6.4 Hz), 6.96 – 7.04 (m, 1H), 7.26 – 7.42 (m, 3H), 7.45 (s, 1H).

**MS (*m/z*):** 231 [M + H]^+^, 253 [M + Na]^+^.

***3-( 3-bromophenyl)-6,7-dihydro-5H-benzofuran-4-one* DD700**

Yield 71%; mp 106-107°C recrystallized from heptane.

**IR (CDCl_3_) ν_max_ (cm^-1^):** 1678, 1220

**^1^H NMR (CDCl_3_) δ (ppm):** 2.20 (quint, 2H, *J* = 6.5 Hz ), 2.55 (br t, 2H, *J* = 6.5 Hz), 2.93 (t, 2H, *J* = 6.5 Hz), 7.24 (t, 1H, *J* = 7.9 Hz), 7.43 (s,1H), 7.44 (ddd, 1H, *J* = 1.1 Hz, 1.8 Hz and 7.9 Hz), 7.58 (dt, 1H, *J* = 1.1 Hz and 7.9 Hz), 7.76 (t, 1H, J = 1.8 Hz).

**MS (*m/z*):** 291-293 [M + H]^+^, 313-315 [M + Na]^+^.

***3-( 3-iodophenyl)-6,7-dihydro-5H-benzofuran-4-one* GAC25**

Yield 79%; mp 135-136°C recrystallized from heptane.

**IR (CDCl_3_) ν_max_ (cm^-1^):** 1675, 1220

**^1^H NMR (CDCl_3_) δ (ppm):** 2.20 (quint, 2H, *J* = 6.4 Hz ), 2.55 (br t, 2H, *J* = 6.4 Hz), 2.93 (t, 2H, *J* = 6.4 Hz), 7.11 (t, 1H, *J* = 7.8 Hz), 7.43 (s,1H), 7.59 – 7.67 (m, 2H), 7.96 (t, 1H, J = 1.7 Hz).

**MS (*m/z*):** 361 [M + Na]^+^.

***3-(3-iodo-4-methoxyphenyl)-6,7-dihydro-5H-benzofuran-4-one* GAC18**

Yield 76%; mp 197-198°C recrystallized from a benzene/heptane mixture.

**IR (CDCl_3_) ν_max_ (cm^-1^):** 1673, 1488, 1215

**^1^H NMR (CDCl_3_) δ (ppm):** 2.20 (quint, 2H, *J* = 6.4 Hz ), 2.54 (br t, 2H, *J* = 6.4 Hz), 2.92 (t, 2H, *J* = 6.4 Hz), 3.89 (s, 3H), 6.84 (d, 1H, J = 8.4 Hz), 7.37 (s, 1H), 7.65 (dd, 1H, *J* = 2.1 Hz and 8.4 Hz), 8.00 (d, 1H, *J* = 2.1 Hz).

**MS (*m/z*):** 391 [M + Na]^+^.

***3-(3-trifluorophenyl)-6,7-dihydro-5H-benzofuran-4-one* GAC11**

Yield 85%; mp 134-135°C recrystallized from heptane.

**IR (CDCl_3_) ν_max_ (cm^-1^):** 1776, 1726, 1678, 1216

**^1^H NMR (CDCl_3_) δ (ppm):** 2.21 (quint, 2H, *J* = 6.4 Hz ), 2.57 (br t, 2H, *J* = 6.4 Hz), 2.95 (t, 2H, *J* = 6.4 Hz), 7.49 (s,1H), 7.45 – 7.60 (m, 2H), 7.84 (d, 1H, *J* = 7.7 Hz), 7.88 (br s, 1H).

**MS (*m/z*):** 303 [M + Na]^+^.

***3-m-tolyl-6,7-dihydro-5H-benzofuran-4-one* GAC22**

Yield 80 %; mp 74-75 °C recrystallized from hexane

**IR (CDCl_3_) ν_max_ (cm^-1^):** 2956, 1675, 1403, 1108

**^1^H NMR (CDCl_3_) δ (ppm):** 2.20 (quint, 2H, *J* = 6.3 Hz ), 2.38 (s, 3H), 2.55 (br t, 2H, *J* = 6.3 Hz), 2.93 (t, 2H, *J* = 6.3 Hz), 7.13 (br d, 1H, *J* = 7.5 Hz), 7.27 (br t, 1H, *J* = 7.5 Hz), 7.39 – 7.44 (m, 2H), 7.41 (s, 1H).

**MS (*m/z*):** 249 [M + Na]^+^.

***3-(3-aminophenyl)-6,7-dihydro-5H-benzofuran-4-one* GAC35**

Yield 96 %; mp 118-119 °C recrystallized from a benzene/heptane mixture.

**IR (CDCl_3_) ν_max_ (cm^-1^):** 3400, 2956, 1674, 1619, 1458, 1409, 1104

**^1^H NMR (CDCl_3_) δ (ppm):** 2.19 (quint, 2H, *J* = 6.3 Hz ), 2.54 (br t, 2H, *J* = 6.3 Hz), 2.92 (t, 2H, *J* = 6.3 Hz), 3.00 – 3.80 (m, 2H, exchangeable with D_2_O), 6.65 (ddd, 1H, *J* = 1.0 Hz, 2.2Hz and 7.9 Hz), 6.97 (ddd, 1H, *J* = 1.0 Hz, 1.5Hz and 7.6 Hz), 7.03 (dd, 1H, *J* = 1.5 Hz and 2.2 Hz), 7.15 (br t, 1H, *J* = 7.7 Hz), 7.41 (s, 1H).

**MS (*m/z*):** 227 [M + H]^+^, 250 [M + Na]^+^.

***3-(3-nitrophenyl)-6,7-dihydro-5H-benzofuran-4-one* DD718**

Yield 63%; mp 94-95°C recrystallized from a benzene/heptane mixture.

**IR (CDCl_3_) ν_max_ (cm^-1^):** 1678, 1529, 1350, 1216

**^1^H NMR (CDCl_3_) δ (ppm):** 2.23 (quint, 2H, *J* = 6.3 Hz ), 2.57 (br t, 2H, *J* = 6.3 Hz), 2.96 (t, 2H, *J* = 6.3 Hz), 7.54 (t, 1H, *J* = 8.0 Hz), 7.55 (s, 1H), 8.01 (dt, 1H, *J* = 1.1 Hz and 8.0 Hz), 8.16 (ddd, 1H, *J* = 1.1 Hz, 2.0 Hz and 8.0 Hz), 8.48 (t, 1H, J = 2.0 Hz).

**MS (*m/z*):** 258 [M + H]^+^, 280 [M + Na]^+^.

***3-(3-cyanophenyl)-6,7-dihydro-5H-benzofuran-4-one* DD762**

Yield 86 %; mp 109-110 °C recrystallized from heptane

**IR (CDCl_3_) ν_max_ (cm^-1^):** 2233, 1676, 1555, 1402, 1108

**^1^H NMR (CDCl_3_) δ (ppm):** 2.22 (quint, 2H, *J* = 6.3 Hz ), 2.56 (br t, 2H, *J* = 6.3 Hz), 2.95 (t, 2H, *J* = 6.3 Hz), 7.47 (s, 1H), 7.48 (t, 1H, *J* = 7.8 Hz), 7.59 (br d, 1H, *J* = 7.8 Hz), 785-7.92 (m, 2H).

**MS (*m/z*):** 238 [M + H]^+^, 260 [M + Na]^+^.

**3-(3-ethenylphenyl)-6,7-dihydro-5H-benzofuran-4-one GAC50**

Yield 78 %; mp 88-89 °C recrystallized from heptane

**IR (CDCl_3_) ν_max_ (cm^-1^):** 2360, 1675, 1411, 1108

**^1^H NMR (CDCl_3_) δ (ppm):** 2.21 (quint, 2H, *J* = 6.3 Hz ), 2.56 (br t, 2H, *J* = 6.3 Hz), 2.94 (t, 2H, *J* = 6.3 Hz), 5.26 (brd, 1H, *J* = 10.9 Hz), 5.79 (dd, 1H, *J* = 0.6 Hz and 17.6 Hz), 6.74 (dd, 1H, *J* = 10.9 Hz and 17.6 Hz), 7.30 – 7.40 (m, 2H), 7.45 (s, 1H), 7.51 (dt, 1H, *J* = 1.8 Hz and 6.8 Hz), 7.68 (br s, 1H).

**MS (*m/z*):** 261 [M + Na]^+^.

***3-(3-methoxyphenyl)-6,7-dihydro-5H-benzofuran-4-one* DD710**

Yield 85%; mp 79-80°C recrystallized from heptane.

**IR (CDCl_3_) ν_max_ (cm^-1^):** 1674, 1221

**^1^H NMR (CDCl_3_) δ (ppm):** 2.20 (quint, 2H, *J* = 6.4 Hz ), 2.55 (br t, 2H, *J* = 6.4 Hz), 2.93 (t, 2H, *J* = 6.4 Hz), 3.85 (s, 3H), 6.87 (ddd, 1H, *J* = 1.0 Hz, 2.6 Hz and 8.2 Hz), 7.17 (dt, 1H, *J* = 1.2 Hz and 7.7 Hz), 7.24 – 7.31 (m, 2H), 7.45 (s, 1H).

**MS (*m/z*):** 243 [M + H]^+^, 265 [M + Na]^+^.

***3-(3,4,5-trimethoxyphenyl)-6,7-dihydro-5H-benzofuran-4-one* GAC14**

Yield 83%; mp 140-141°C recrystallized from a benzene/heptane mixture.

**IR (CDCl_3_) ν_max_ (cm^-1^):** 1663, 1505, 1130

**^1^H NMR (CDCl_3_) δ (ppm):** 2.21 (quint, 2H, *J* = 6.3 Hz ), 2.56 (br t, 2H, *J* = 6.3 Hz), 2.94 (t, 2H, *J* = 6.3 Hz), 3.87 (s, 3H), 3,90 (s,6H), 7.26 (s, 2H), 7.46 (s, 1H).

**MS (*m/z*):** 325 [M + Na]^+^.

***3-benzo[1,3]dioxol-5-yl-6,7-dihydro-5H-benzofuran-4-one* GAC19**

Yield 76 %; mp 99-100 °C recrystallized from a benzene/hexane mixture.

**IR (CDCl_3_) ν_max_ (cm^-1^):** 2957, 2992, 1675, 1503, 1486, 1232, 1042

**^1^H NMR (CDCl_3_) δ (ppm):** 2.19 (quint, 2H, *J* = 6.3 Hz ), 2.54 (br t, 2H, *J* = 6.3 Hz), 2.92 (t, 2H, *J* = 6.3 Hz), 5.97 (s, 2H), 6.81 (d, 1H, *J* = 7.9 Hz), 7.09 (dd, 1H, *J* = 1.8 Hz and 7.9 Hz), 7.12 (d, 1H, *J* = 1.8 Hz), 7.36 (s, 1H).

**MS (*m/z*):** 257 [M + H]^+^, 279 [M + Na]^+^.

***3-naphthalen-2-yl-6,7-dihydro-5H-benzofuran-4-one* GAC45**

Yield 83 %; mp 97-98 °C recrystallized from a benzene/heptane mixture.

**IR (CDCl_3_) ν_max_ (cm^-1^):** 2957, 1674, 1411, 1102

**^1^H NMR (CDCl_3_) δ (ppm):** 2.23 (quint, 2H, *J* = 6.3 Hz ), 2.59 (br t, 2H, *J* = 6.3 Hz), 2.97 (t, 2H, *J* = 6.3 Hz), 7.42-7.51 (m, 2H), 7.70 (dd, 1H, *J* = 1.2 Hz and 8.5 Hz), 7.79-7.90 (m, 3H), 8.17 (s, 1H).

**MS (*m/z*):** 285 [M + Na]^+^.

***3-(3-Hydroxy-phenyl)-6,7-dihydro-5H-benzofuran-4-one* JP45**

Yield 83 %; mp 93-94 °C recrystallized from a benzene/heptane mixture.

**IR (CDCl_3_) ν_max_ (cm^-1^):** 3147, 1650, 1479, 1239, 1102, 1011

**^1^H NMR (CDCl_3_) δ (ppm):** 2.26 (quint, 2H, *J* = 6.4 Hz ), 2.65 (br t, 2H, *J* = 6.4 Hz), 3.00 (t, 2H, *J* = 6.4 Hz), 6.97 (dt, 1H, *J* = 1.2 Hz and 7.4 Hz), 7.14 (dd, 1H, *J* = 1.1 Hz, 8.1 Hz), 7.23 (dd, 1H, *J* = 1.7 Hz and 7.6 Hz), 7.31 (ddd, 1H, *J* = 1.7 Hz, 7.4 Hz and 8.1 Hz), 7.42 (s, 1H), 8.24 (brs, 1H).

**MS (*m/z*):** 251 [M + Na]^+^.

***3-(3-benzyloxyphenyl)-6,7-dihydro-5H-benzofuran-4-one* GAC13**

Yield 74%; yellow oil.

**IR (CDCl_3_) ν_max_ (cm^-1^):** 1674, 1234

**^1^H NMR (CDCl_3_) δ (ppm):** 2.20 (quint, 2H, *J* = 6.4 Hz ), 2.56 (br t, 2H, *J* = 6.4 Hz), 2.93 (t, 2H, *J* = 6.4 Hz), 5.12 (s, 2H), 6.95 (ddd, 1H, *J* = 1.3 Hz, 2.6 Hz and 8.2 Hz), 7.21 (dt, 1H, *J* = 1.3 Hz and 7.8 Hz), 7.24 – 7.50 (m, 8H).

**MS (*m/z*):** 341 [M + Na]^+^.

***3-(3-chlorophenyl)-6-phenyl-6,7-dihydro-5H-benzofuran-4-one* GAC46**

Yield 73 %; mp 115-116 °C recrystallized from a benzene/heptane mixture.

**IR (CDCl_3_) ν_max_ (cm^-1^):** 2360, 1679, 1562, 1440, 1109

**^1^H NMR (CDCl_3_) δ (ppm):** 2.78 - 2.92 (m, 2H), 3.06 - 3.30 (m, 2H), 3.55 – 3.68 (m, 1H), 7.27 - 7.42 (m, 7H), 7.50 (s, 1H), 7.53 – 7.61 (m, 1H), 7.63 – 7.67 (m, 1H).

**MS (*m/z*):** 323-325 [M + H]^+^. 345-347 [M + Na]^+^.

***3-(3-chlorophenyl)-6-(4-chlorophenyl)-6,7-dihydro-5H-benzofuran-4-one* GAC 36**

Yield 85 %; mp 137-138°C recrystallized from heptane

**IR (CDCl_3_) ν_max_ (cm^-1^):** 2359, 1680, 1563, 1493, 1441, 1109

**^1^H NMR (CDCl_3_) δ (ppm):** 2.75 - 2.84 (m, 2H), 3.02 - 3.29 (m, 2H), 3.52 - 3.67 (m, 1H), 7.21 – 7.26 (m, 2H), 7.26 - 7.38 (m, 4H) 7.50 (s, 1H), 7.52 – 7.58 (m, 1H), 7.62 - 7.65 (m, 1H).

**MS (*m/z*):** 379-381-383 [M + Na]^+^.

***3-(3-chlorophenyl)-6-furan-2-yl-6,7-dihydro-5H-benzofuran-4-one* GAC37**

Yield 75 %; mp 105-106 °C recrystallized from heptane

**IR (CDCl_3_) ν_max_ (cm^-1^):** 2360, 1680, 1564, 1442, 1109

**^1^H NMR (CDCl_3_) δ (ppm):** 2.73 - 2.96 (m, 2H), 3.11 - 3.41 (m, 2H), 3.63 - 3.77 (m, 1H), 6.10 – 6.14 (m, 1H), 6.30 – 6.34 (m, 1H), 7.27 - 7.32 (m, 2H), 7.34 - 7.38 (m, 1H) 7.48 (s, 1H), 7.51 – 7.56 (m, 1H), 7.60 - 7.64 (m, 1H).

**MS (*m/z*):** 313-315 [M + H]^+^. 335-337 [M + Na]^+^.

***3-(3-chlorophenyl)-6,6-dimethyl-6,7-dihydro-5H-benzofuran-4-one* DD694**

Yield 77%; mp 104-105°C recrystallized from heptane.

**IR (CDCl_3_) ν_max_ (cm^-1^):** 1677, 1215

**^1^H NMR (CDCl_3_) δ (ppm):** 1.17 (s, 6H), 2.44 (s, 2H), 2.80 (s, 2H), 7.27 – 7.34 (m, 2H), 7.47 (s, 1H), 7.53 – 7.58 (m,1H), 7.64 (dd, 1H, *J* = 0.5 Hz and 1.8 Hz).

**MS (*m/z*):** 275-277 [M + H]^+^, 297-299 [M + Na]^+^.

***3-(3-chlorophenyl)-6-methyl-6,7-dihydro-5H-benzofuran-4-one* DD720**

Yield 81%; mp 121-122°C (with allotropic change at 116-118°C) recrystallized from heptane.

**IR (CDCl_3_) ν_max_ (cm^-1^):** 1676

**^1^H NMR (CDCl_3_) δ (ppm):** 1.20 (d, 3H, J = 6.0 Hz), 2.31 (dd, 1H, *J* = 10.4 Hz and 15.5 Hz), 2.39 – 2.66 (m, 3H), 3.02 (dd, 1H, *J* = 4.5 Hz and 15.9 Hz), 7.24 – 7.34 (m, 2H), 7.45 (s, 1H), 7.49 – 7.57 (m,1H), 7.62 (brs, 1H).

**MS (*m/z*):** 261-263 [M + H]^+^, 283-285 [M + Na]^+^.

***3-(3-chlorophenyl)-5,5-dimethyl-6,7-dihydro-5H-benzofuran-4-one* DD772**

Yield 32 %; mp 172-174 °C recrystallized from heptane

**IR (CDCl_3_) ν_max_ (cm^-1^):** 2957, 1675, 1565, 1438, 1111

**^1^H NMR (CDCl_3_) δ (ppm):** 1.42 (s, 6H), 2.03 (t, 2H, *J* = 6.4 Hz), 2.62 (t, 2H, *J* = 6.4 Hz), 7.27 - 7.32 (m, 2H), 7.42 (s, 1H), 7.47 - 7.53 (m, 1H), 7.57 - 7.61 (m, 1H).

**MS (*m/z*):** 297-299 [M + Na]^+^

***3-(3-chlorophenyl)-7,7-dimethyl-6,7-dihydro-5H-benzofuran-4-one* JP14**

Yield 35 %; colorless oil

**IR (CDCl_3_) ν_max_ (cm^-1^):** 2966, 2939, 1675, 1566, 1436, 1094.

**^1^H NMR (CDCl_3_) δ (ppm):** 1.20 (s, 6H), 2.04 (t, 2H, *J* = 6.4 Hz), 2.95 (t, 2H, *J* = 6.4 Hz), 7.27 - 7.31 (m, 2H), 7.43 (s, 1H), 7.48 - 7.54 (m, 1H), 7.57 - 7.61 (m, 1H).

**MS (*m/z*):** 275-277 [M + H]^+^, 297-299 [M + Na]^+^.

***6,6-dimethyl-3-(2-nitrophenyl)-6,7-dihydro-5H-benzofuran-4-one* DD287**

Yield 79 %; mp 135-136 °C (allotropic change 128-131°C) recrystallized from heptane

**IR (CDCl_3_) ν_max_ (cm^-1^):** 1666, 1519, 1346

**^1^H NMR (CDCl_3_) δ (ppm):** 1.16 (s, 6H), 2.33 (s, 2H), 2.80 (s, 2H), 7.40 (dd, 1H, *J* = 1.6Hz and 7.5 Hz), 7.42 (s, 1H), 7.50 (dt, 1H, *J* = 1.4Hz and 8.0 Hz), 7.59 (dt, 1H, *J* = 1.4 Hz and 7.5 Hz), 8.13 (dd, 1H, *J* = 1.4Hz and 8.0 Hz).

**MS (*m/z*):** 286 [M + H]^+^, 308 [M + Na]^+^.

Compounds **JP20, JP17, JP13, JP11, JP33, JP15, JP23, JP27, JP30, JP4, JP8 and JP54** were synthesized on a 3 mmol scale by applying classical pallado-catalysed methodologies starting from the bromo (**DD700)** or the iodo derivative (**GAC25)** and the appropriate boronic acids [3,4], alkenes [5,6,7] or alkynes [8]. The desilylated derivative **JP6** was easily obtained by the treatment of **JP4** with potassium carbonate at room temperature [8].

***3-(3-styrylphenyl)-6,7-dihydro-5H-benzofuran-4-one* JP20**

Yield 57 %; mp 99-100°C recrystallized from hexane

**IR (CDCl_3_) ν_max_ (cm^-1^):** 2360, 1674, 1454, 1107

**^1^H NMR (CDCl_3_) δ (ppm):** 2.23 (quint, 2H, *J* = 6.3 Hz ), 2.57 (br t, 2H, *J* = 6.3 Hz), 2.95 (t, 2H, *J* = 6.3 Hz), 7.15 (s, 2H), 7.22 - 7.30 (m, 1H), 7.32 - 7.42 (m, 2H), 7.46 - 7.57 (m, 4H), 7.48 (s, 1H), 7.80 (t, 1H, *J* = 1.6 Hz).

**MS (*m/z*):** 315 [M + H]^+^, 337 [M + Na]^+^.

***3-(2'-chlorobiphenyl-3-yl)-6,7-dihydro-5H-benzofuran-4-one* JP17**

Yield 97 %; mp 152-153 °C recrystallized from a benzene/heptane mixture.

**IR (CDCl_3_) ν_max_ (cm^-1^):** 2955, 1674, 1430, 1109

**^1^H NMR (CDCl_3_) δ (ppm):** 2.21 (quint, 2H, *J* = 6.3 Hz ), 2.57 (br t, 2H, *J* = 6.3 Hz), 2.94 (t, 2H, *J* = 6.3 Hz), 7.24 - 7.37 (m, 2H), 7.40 - 7.49 (m, 4H), 7.47 (s, 1H), 7.62-7.71 (m, 2H).

**MS (*m/z*):** 345-347 [M + Na]^+^

***3-(3'-chlorobiphenyl-3-yl)-6,7-dihydro-5H-benzofuran-4-one* JP13**

Yield 87 %; mp 95-97 °C recrystallized from hexane

**IR (CDCl_3_) ν_max_ (cm^-1^):** 2957, 1674, 1437, 1006

**^1^H NMR (CDCl_3_) δ (ppm):** 2.22 (quint, 2H, *J* = 6.3 Hz ), 2.58 (br t, 2H, *J* = 6.3 Hz), 2.96 (t, 2H, *J* = 6.3 Hz), 7.28 - 7.64 (m, 7H), 7.49 (s, 1H), 7.84 -7.88 (m, 1H).

**MS (*m/z*):** 323-325 [M + H]^+^, 345-347 [M + Na]^+^

***3-(4'-chlorobiphenyl-3-yl)-6,7-dihydro-5H-benzofuran-4-one* JP11**

Yield 96 %; mp 81-82 °C recrystallized from hexane

**IR (CDCl_3_) ν_max_ (cm^-1^):** 1673, 1479, 1094, 1014

**^1^H NMR (CDCl_3_) δ (ppm):** 2.23 (quint, 2H, *J* = 6.3 Hz ), 2.58 (br t, 2H, *J* = 6.3 Hz), 2.96 (t, 2H, *J* = 6.3 Hz), 7.36 - 7.62 (m, 7H), 7.49 (s, 1H), 7.90 (br s, 1H).

**MS (*m/z*):** 323-325 [M + H]^+^, 345-347 [M + Na]^+^

***3-(3'-bromobiphenyl-3-yl)-6,7-dihydro-5H-benzofuran-4-one* JP33**

Yield 85 %; mp 105-106°C recrystallized from hexane

**IR (CDCl_3_) ν_max_ (cm^-1^):** 2360, 1674, 1562, 1412, 1109

**^1^H NMR (CDCl_3_) δ (ppm):** 2.23 (quint, 2H, *J* = 6.4 Hz ), 2.58 (br t, 2H, *J* = 6.4 Hz), 2.96 (t, 2H, *J* = 6.4 Hz), 7.31 (t, 1H, *J* = 7.9 Hz), 7.41 - 7.54 (m, 3H), 7.49 (s, 1H), 7.56 - 7.63 (m, 2H), 7.78 (t, 1H, *J* = 1.7 Hz), 7.86 (t, 1H, *J* = 1.3 Hz).

**MS (*m/z*):** 367-369 [M + H]^+^, 389-391 [M + Na]^+^.

***3-[3-(4-oxo-4,5,6,7-tetrahydrobenzofuran-3-yl)phenyl]acrylic acid methyl ester* JP15**

Yield 56 %; mp 109-110 °C recrystallized from heptane

**IR (CDCl_3_) ν_max_ (cm^-1^):** 2953, 1708, 1675, 1640 1437, 1279, 1176, 1108

**^1^H NMR (CDCl_3_) δ (ppm):** 2.22 (quint, 2H, *J* = 6.3 Hz ), 2.56 (br t, 2H, *J* = 6.3 Hz), 2.95 (t, 2H, *J* = 6.3 Hz), 3.81 (s, 3H), 6.49 (d, 1H, *J* = 16.0 Hz), 7.39 (t, 1H, *J* = 7.6 Hz), 7.44 - 7.50 (m, 1H), 7.46 (s, 1H), 7.64 (dt, 1H, *J* = 1.6 Hz and 7.6 Hz), 7.73 (d, 1H, *J* = 16.0 Hz), 7.82 (br t, 1H, *J* = 1.6 Hz).

**MS (*m/z*):** 319 [M + Na]^+^

***3-[3-(4-oxo-4,5,6,7-tetrahydrobenzofuran-3-yl)phenyl]acrylamide* JP23**

Yield 53 %; mp 166-167 °C recrystallized from a benzene/heptane mixture.

**IR (CDCl_3_) ν_max_ (cm^-1^):** 3690, 3528, 3412, 2360, 1678, 1632, 1586, 1373, 1108

**^1^H NMR (CDCl_3_) δ (ppm):** 2.22 (quint, 2H, *J* = 6.3 Hz ), 2.57 (br t, 2H, *J* = 6.3 Hz), 2.97 (t, 2H, *J* = 6.3 Hz), 5.40 -5.75 (massif, 2H), 6.52 (d, 1H, *J* = 15.7 Hz), 7.38 (t, 1H, *J* = 7.6 Hz), 7.44 (dt, 1H, *J* = 1.4 Hz and 7.6 Hz), 7.47 (s, 1H), 7.60 (dt, 1H, *J* = 1.5 Hz and 7.4 Hz), 7.67 (d, 1H, *J* = 15.7 Hz), 7.89 (br s, 1H).

**MS (*m/z*):** 304 [M + Na]^+^

***3-[3-(4-oxo-4,5,6,7-tetrahydrobenzofuran-3-yl)phenyl] acrylonitrile* JP27**

Yield 80 %; colorless oil

**IR (CDCl_3_) ν_max_ (cm^-1^):** 2958, 2221, 1674, 1620, 1436, 1407, 1373, 1108

**^1^H NMR (CDCl_3_) δ (ppm):** 2.23 (quint, 2H, *J* = 6.3 Hz ), 2.57 (br t, 2H, *J* = 6.3 Hz), 2.96 (t, 2H, *J* = 6.3 Hz), 5.95 (d, 1H, *J* = 16.7 Hz), 7.35 – 7.50 (m, 3H), 7.48 (s, 1H), 7.64 (dt, 1H, *J* = 1.8 Hz and 6.8 Hz), 7.83 (br s, 1H).

**MS (*m/z*):** 286 [M + Na]^+^

***3-(3-oct-1-enylphenyl)-6,7-dihydro-5H-benzofuran-4-one* JP30**

Yield 79 %; colorless oil

**IR (CDCl_3_) ν_max_ (cm^-1^):** 2958, 2929, 1674, 1456,1107

**^1^H NMR (CDCl_3_) δ (ppm):** 0.82 – 0.93 (m, 3H), 1.20 -1.62 (m, 8H), 2.13 -2.27 (m, 4H), 2.55 (br t, 2H, *J* = 6.3 Hz), 2.95 (t, 2H, *J* = 6.3 Hz), 6.20 – 6.44 (m, 2H), 7.23 – 7.32 (m, 2H), 7.41 – 7.48 (m, 1H), 7.42 (s, 1H), 7.61 (br s, 1H).

**MS (*m/z*):** 323 [M + H]^+^, 345 [M + Na]^+^

***3-(3-trimethylsilanylethynylphenyl)-6,7-dihydro-5H-benzofuran-4-one* JP4**

Yield 86 %; mp 117-118 °C recrystallized from heptane

**IR (CDCl_3_) ν_max_ (cm^-1^):** 2956, 1678, 1249, 1107

**^1^H NMR (CDCl_3_) δ (ppm):** 0.25 (s, 9H), 2.21 (quint, 2H, *J* = 6.3 Hz), 2.55 (br t, 2H, *J* = 6.3 Hz), 2.93 (t, 2H, *J* = 6.3 Hz), 7.31 (dt, 1H, *J* = 0.5 Hz and 7.7 Hz), 7.41 (dt, 1H, *J* = 1.4 Hz and 7.7 Hz), 7.44 (s, 1H), 7.64 (dt, 1H, *J* = 1.4 Hz and 7.7 Hz), 7.64 – 7.68 (m, 1H).

**MS (*m/z*):** 331 [M + Na]^+^

***3-(3-ethynylphenyl)-6,7-dihydro-5H-benzofuran-4-one* JP6**

Yield 68 %; mp 91-92°C recrystallized from heptane

**IR (CDCl_3_) ν_max_ (cm^-1^):** 3305, 2957, 1675, 1396, 1107

**^1^H NMR (CDCl_3_) δ (ppm):** 2.21 (quint, 2H, *J* = 6.3 Hz), 2.55 (br t, 2H, *J* = 6.3 Hz), 2.94 (t, 2H, *J* = 6.3 Hz), 3.06 (s, 1H), 7.34 (br t, 1H, 7.7 Hz), 7.44 (dt, 1H, *J* = 1.4 Hz and 7.7 Hz), 7.44 (s, 1H), 7.66 (dt, 1H, *J* = 1.4 Hz and 7.7 Hz), 7.72 (br t, 1H, *J* = 1.5 Hz ).

**MS (*m/z*):** 259 [M + Na]^+^

***3-[3-(4-methylpent-1-ynyl)phenyl]-6,7-dihydro-5H-benzofuran-4-one* JP8**

Yield 70 %; mp 41-43 °C recrystallized from hexane

**IR (CDCl_3_) ν_max_ (cm^-1^):** 2960, 1675, 1430, 1107

**^1^H NMR (CDCl_3_) δ (ppm):** 1.04 (d, 6H, *J*= 6.6 Hz), 1.91 (hept, 1H, *J*= 6.6 Hz), 2.20 (quint, 2H, *J* = 6.3 Hz), 2.30 (d, 2H, *J*= 6.6 Hz), 2.55 (br t, 2H, *J* = 6.3 Hz), 2.93 (t, 2H, *J* = 6.3 Hz), 7.24 – 7.38 (m, 2H), 7.43 (s, 1H), 7.54 – 7.63 (m, 2H).

**MS (*m/z*):** 293 [M + H]^+^, 315 [M + Na]^+^

***3-[3-(3-chlorophenylethynyl)phenyl]-6,7-dihydro-5H-benzofuran-4-one* JP54**

Yield 62 %; mp 108-109 °C recrystallized from hexane

**IR (CDCl_3_) ν_max_ (cm^-1^):** 2957, 1674, 1594, 1106

**^1^H NMR (CDCl_3_) δ (ppm):** 2.21 (quint, 2H, *J* = 6.3 Hz ), 2.56 (br t, 2H, *J* = 6.3 Hz), 2.94 (t, 2H, *J* = 6.3 Hz), 7.23 -7.49 (m, 5H), 7.46 (s, 1H), 7.53 (br t, 1H, *J* = 1.7 Hz), 7.65 (dt, 1H, *J* = 1.4 Hz and 7.7 Hz), 7.76 (br t, 1H, *J* = 1.5 Hz).

**MS (*m/z*):** 347-349 [M + H]^+^, 369-371 [M + Na]^+^

The ester **JP50** was synthesized on a 5 mmol scale in a convetional manner from **DD700** and ethyl bromoacetate in anhydrous basic medium [9]. Subsequent saponification of **JP50** provided the acid **JP29.**

**[3-(3-bromophenyl)-4-oxo-4,5,6,7-tetrahydrobenzofuran-5-yl]acetic acid ethyl ester JP50**

Yield 37 %; colorless oil

**IR (CDCl_3_) ν_max_ (cm^-1^):** 2929, 1728, 1681, 1561,1429, 1377, 1190

**^1^H NMR (CDCl_3_) δ (ppm):** 1.27 (t, 3H, *J* = 7.1 Hz), 1.97 – 2.18 (m, 1H), 2.25 - 2.44 (m, 2H), 2.92 – 3.08 (m, 4H), 4.17 (q, 2H, *J* = 7.1 Hz), 7.22 (d, 1H, *J* = 7.8 Hz ), 7.41 - 7.46 (m, 1H), 7.43 (s, 1H), 7.55 (dt, 1H, *J* = 1.4 Hz and 7.7 Hz), 7.71 (t, 1H, *J* = 1.7 Hz).

**MS (*m/z*):** 399-401 [M + Na]^+^.

**[3-(3-bromophenyl)-4-oxo-4,5,6,7-tetrahydrobenzofuran-5-yl]acetic acid JP29**

Yield 72 %; mp 118-119 °C recrystallized from a toluene/heptane mixture.

**IR (CDCl_3_) ν_max_ (cm^-1^):** 3691, 1745, 1689, 1602

**^1^H NMR (CDCl_3_) δ (ppm):** 2.01 - 2.19 (m, 1H), 2.27 - 2.38 (m, 1H), 2.39 – 2.52 (m, 1H), 2.92 – 3.10 (m, 4H), 7.24 (t, 1H, *J* = 7.9 Hz ), 7.40 - 7.47 (m, 1H), 7.43 (s, 1H), 7.55 (br d, 1H, *J* = 7.8 Hz), 7.70 (t, 1H, *J* = 1.7 Hz), the OH proton is indiscernible.

**MS (*m/z*):** 371-373 [M + Na]^+^.

The 5-bromobenzofuranone **JP61f2** was obtained by action of copper(II) bromide in methanol on the chloro derivative **DD264** [10].

***5-bromo-3-(3-chlorophenyl)-6,7-dihydro-5H-benzofuran-4-one* JP61f2**

Yield 42 %; mp120-121 °C recrystallized from hexane

**IR (CDCl_3_) ν_max_ (cm^-1^):** 2924, 1686, 1565, 1445, 1113

**^1^H NMR (CDCl_3_) δ (ppm):** 2.45 - 2.65 (m, 2H), 2.93 (ddd, 1H, *J* = 2.8 Hz, 4.7 Hz and 17.7 Hz), 3.13 – 3.28 (m, 1H), 4.58 (t, 1H, *J* = 3.8 Hz ), 7.27 - 7.55 (m, 2H), 7.44 - 7.53 (m, 1H), 7.49 (s, 1H), 7.57 – 7.60 (m, 1H).

**MS (*m/z*):** 347-349-351 [M + Na]^+^.

The 5-dimethylaminomethylfuranone **JP67** was prepared on a 3 mmol scale from **DD264**  by treatment with dimethylammonium chloride and 1,3,5-trioxane in refluxing isopropanol [11].

***3-(3-chlorophenyl)-5-dimethylaminomethyl-6,7-dihydro-5H-benzofuran-4-one* JP67**

Yield 31 %; pale yellow oil

**IR (CDCl_3_) ν_max_ (cm^-1^):** 2946, 1676, 1565, 1456, 1436, 1114

**^1^H NMR (CDCl_3_) δ (ppm):** 1.85 - 2.14 (m, 1H), 2.27 (s, 6H), 2.34 - 2.59 (m, 2H), 2.60 - 2.80 (m, 2H), 2.85 – 3.12 (m, 2H), 7.23 - 7.34 (m, 2H), 7.43 (s, 1H), 7.46 - 7.55 (m, 1H), 7.59 (br s, 1H).

**MS (*m/z*):** 304-306 [M + H]^+^.

The indole derivative **GAC21** was prepared on a 5 mmol scale starting from cyclohexane-1,3-dione and 3-(2-Chloro-2-nitroethenyl)-1*H*-indole following our formerly decribed procedure [1].

***3-(1H-Indol-3-yl)-2-nitro-3,5,6,7-tetrahydro-2H-benzofuran-4-one* GAC21**

Yield 80 %; mp 191-192 °C recrystallized from a benzene/heptane mixture.

**IR (CDCl_3_) ν_max_ (cm^-1^):** 3476, 2959, 1656, 1570, 1393, 1177

**^1^H NMR (CDCl_3_) δ (ppm):** 2.12 - 2.33 (m, 2H), 2.47 (br t, 2H, *J* = 6.4 Hz), 2.72 – 2.92 (m, 2H), 4.92 (br s, 1H), 6.09 (d, 1H, *J* = 2.2 Hz), 6.91 (d, 1H, *J* = 2.2 Hz), 7.14 - 7.27 (m, 2H), 7.38 (d, 1H, *J* = 7.8 Hz), 7.69 (dd, 1H, *J* = 0.6 Hz and 7.8 Hz ), 8.30 (br s, 1H).

**MS (*m/z*):** 321 [M + Na]^+^.

The 3-heteroaryl-6,7-dihydro-5*H*-benzofuran-4-ones **GAC30** and **DD765** were prepared on a 5 mmol scale from cyclohexane-1,3-dione and the appropriate (*Z*)-β-chloro-β-nitrostyrenes according to the procedure previously published [2].

***3-(1H-Indol-3-yl)-6,7-dihydro-5H-benzofuran-4-one* GAC30**

Yield 61 %; mp 185-186 °C recrystallized from a benzene/heptane mixture.

**IR (CDCl_3_) ν_max_ (cm^-1^):** 3477, 2956, 1671, 1434, 1086

**^1^H NMR (CDCl_3_) δ (ppm):** 2.22 (quint, 2H, *J* = 6.3 Hz), 2.59 (br t, 2H, *J* = 6.3 Hz), 2.96 (t, 2H, 6.3 Hz), 7.15 - 7.27 (m, 2H), 7.42 (dd, 1H, *J* = 1.8 Hz and 7.8 Hz ), 7.75 (dd, 1H, *J* = 1.8 Hz and 7.1 Hz ), 7.86 (s, 1H), 8.29 (d, 1H, *J* = 2.5 Hz), 8.30 – 8.50 (massif, 1H).

**MS (*m/z*):** 274 [M + Na]^+^.

***3-(4-oxo-4,5,6,7-tetrahydrobenzofuran-3-yl)chromen-4-one* DD765**

Yield 77 %; mp 222-224 °C recrystallized from a benzene/heptane mixture.

**IR (CDCl_3_) ν_max_ (cm^-1^):** 1700, 1675, 1608, 1542, 1469, 1281, 1217

**^1^H NMR (CDCl_3_) δ (ppm):** 2.26 (quint, 2H, *J* = 6.3 Hz), 2.78 (br t, 2H, *J* = 6.3 Hz), 3.08 (t, 2H, 6.3 Hz), 7.26 (s,1H), 7.45 - 7.55 (m, 2H), 7.81 (ddd, 1H, *J* = 1.7 Hz, 7.2 Hz and 8.6 Hz ), 8.34 (dd, 1H, *J* = 1.5 Hz and 7.9 Hz ), 9.15 (s, 1H).

**MS (*m/z*):** 303 [M + Na]^+^.

The oxime **GAC12** and the semicarbazone **DD817** were synthesized in a conventional way on a 2.5 mmol scale from the ketone **DD264** and hydroxylamine hydrochloride [12] or semicarbazide hydrochloride [13], respectively.

***3-(3-Chlorophenyl)-6,7-dihydro-5H-benzofuran-4-one oxime* GAC12**

Yield 92%; mp 157-158°C recrystallized from heptane.

**IR (CDCl_3_) ν_max_ (cm^-1^):** 3306, 3019, 1602, 1566, 1425, 1216

**^1^H NMR (CDCl_3_) δ (ppm):** 2.00 (quint, 2H, *J* = 6.5 Hz ), 2.68-2.82 (m, 4H), 7.23-7.28 (m, 2H), 7.29 (s, 1H), 7.33 – 7.40 (m, 1H), 7.50 (br s, 1H).

**MS (*m/z*):** 262-264 [M + H]^+^, 284-286 [M + Na]^+^.

***3-(3-Chlorophenyl)-6,7-dihydro-5H-benzofuran-4-one semicarbazone* DD817**

Yield 78 %; mp 147-148 °C recrystallized from heptane

**IR (CDCl_3_) ν_max_ (cm^-1^):** 3529, 3397, 1689, 1560, 1424, 1138

**^1^H NMR (CDCl_3_) δ (ppm):** 2.07 (quint, 2H, *J* = 6.3 Hz ), 2.48 (br t, 2H, *J* = 6.3 Hz), 2.78 (t, 2H, *J* = 6.3 Hz), 4.30 - 4.90 (massif, 1H), 5.00 - 5.80 (massif, 1H), 7.25 – 7.31 (m, 2H), 7.29 (s, 1H). 7.34 – 7.39 (m, 1H), 7.51 (dd, 1H, *J* = 1.4 Hz and 2.4 Hz), 8.45 (s, 1H).

**MS (*m/z*):** 304-306 [M + H]^+^, 326-328 [M + Na]^+^.

The gem-difluoro derivative **DD747** was synthesized on a 4 mmol scale from the ketone **DD264** and dibromodifluoromethane in the presence of zinc in hexamethylphosphotriamide [14].

***3-(3-chlorophenyl)-4-difluoromethylene-4,5,6,7-tetrahydrobenzofuran* DD747**

Yield 34 %; pale yellow oil

**IR (CDCl_3_) ν_max_ (cm^-1^):** 2932, 1745, 1597, 1283, 1251, 1127

**^1^H NMR (CDCl_3_) δ (ppm):** 1.93 – 2.05 (m, 2H), 2.37 – 2.47 (m, 2H), 2.76 (t, 2H, *J* = 6.4 Hz), 7.15 - 7.23 (m, 1H), 7.23 - 7.33 (m, 3H), 7.29 (s, 1H).

**MS (*m/z*):** 303-305 [M + Na]^+^.

The 3-(3-bromophenyl)-3,5,6,7-tetrahydro-2*H*-benzofuran-4-one (**JP37)** was easily obtained on a 5 mmol scale by Michaël condensation between 1,3-cyclohexanedione and (*E*)-1-bromo-3-(2-nitroethenyl)benzene [15].

***3-(3-bromophenyl)-3,5,6,7-tetrahydro-2H-benzofuran-4-one* JP37**

Yield 64 %; orange oil

**IR (CDCl_3_) ν_max_ (cm^-1^):** 2956, 1630, 1400, 1233, 1180, 1061

**^1^H NMR (CDCl_3_) δ (ppm):** 2.10 (quint, 2H, *J* = 6.4 Hz ), 2.31-2.40 (m, 2H), 2.47-2.64 (m, 2H),4.30 - 4.46 (m, 2H, AB part of ABC system), 4.79-4.88 (t, 1H, *J_app_* = 9.8 Hz, C part of ABC system), 7.10 -7.15 (m, 1H), 7.17 (t, 1H, *J* = 7.6 Hz), 7.30 – 7.37 (m, 2H).

**MS (*m/z*):** 293-295 [M + H]^+^, 315-317 [M + Na]^+^.

The 4-methoxybenzofurane **JP61f1** was isolated as a side product in the synthesis of **JP61f2** (*vide supra*) [10].

***3-(3-chlorophenyl)-4-methoxybenzofuran* JP61f1**

Yield 37 %; colorless oil

**IR (CDCl_3_) ν_max_ (cm^-1^):** 2930, 1600, 1500, 1268, 1114

**H NMR (CDCl_3_) δ (ppm):** 3.86 (s, 3H), 6.72 (d, 1H, *J* = 7.9 Hz), 7.17 (dd, 1H, *J* = 0. 8 Hz and 8.3 Hz), 7.24-7.35 (m, 3H), 7.47 - 7.54 (m, 1H), 7.59 (s, 1H), 7.66 (dd, 1H, *J* = 0. 8 Hz and 1.7 Hz).

**MS (*m/z*):** 281-283 [M + Na]^+^.

The furopyranone **DD789** was prepared on a 5 mmol scale starting from 4-hydroxy-6-methylpyran-2-one and 1-chloro-3-(2-chloro-2-nitroethenyl)benzene according to a known procedure [2].

***3-(3-chlorophenyl)-6-methyl-3a,7a-dihydrofuro[3,2-c]pyran-4-one*** **DD789**

Yield 88 %; mp 153-154 °C recrystallized from a benzene/heptane mixture.

**IR (CDCl_3_) ν_max_ (cm^-1^):** 3140, 1734, 1629, 1451, 1229

**^1^H NMR (CDCl_3_) δ (ppm):** 2.36 (d, 3H, *J* = 0.8 Hz), 6.44 (d, 1H, *J* = 0. 8 Hz), 7.29-7.41 (m, 2H), 7.61 (s, 1H), 7.67 – 7.74 (m, 2H).

**MS (*m/z*):** 261-263 [M + H]^+^, 283-285 [M + Na]^+^.

The 2-(3-chlorophenyl)-6,7-dihydro-5*H*-benzofuran-4-one (**JP55**) was synthesized on a 3 mmol scale from cyclohexane-1,3-dione and 1-Chloro-3-ethynylbenzene in the presence of ammonium cerium (IV) nitrate in basic medium following a described procedure [16].

***2-(3-chlorophenyl)-6,7-dihydro-5H-benzofuran-4-one* JP55**

Yield 51 %; mp112-113°C recrystallized from hexane

**IR (CDCl_3_) ν_max_ (cm^-1^):** 2957, 1674, 1609, 1456, 1133

**^1^H NMR (CDCl_3_) δ (ppm):** 2.22 (quint, 2H, *J* = 6.3 Hz ), 2.53 (br t, 2H, *J* = 6.3 Hz), 2.96 (t, 2H, *J* = 6.3 Hz), 6.91 (s, 1H), 7.23 - 7.29 (m, 1H), 7.32 (t, 1H, *J* = 7.8 Hz ), 7.51 (dt, 1H, *J* = 1.4 Hz and 7.6 Hz), 7.64 (t, 1H, *J* = 1.7 Hz).

**MS (*m/z*):** 247-249 [M + H]^+^, 269-271 [M + Na]^+^.

The 3-(3-bromophenyl)-5,6-dihydro-4*H*-benzofuran-7-one(**DD 829**) was prepared on a 5mmol scale following a recently described methodology [17].

***3-(3-bromophenyl)-5,6-dihydro-4H-benzofuran-7-one* DD829**

Yield 66 %; colorless oil.

**IR (CDCl_3_) ν_max_ (cm^-1^):** 3140, 1735, 1630, 1450, 1230

**^1^H NMR (CDCl_3_) δ (ppm):** .2.21 (quint, 2H, *J* = 6.0 Hz ), 2.63 (br t, 2H, *J* = 6.0 Hz), 2.90 (t, 2H, *J* = 6.0 Hz), 7.25 - 7.37 (m, 2H), 7.49 (dt, 1H, *J* = 1.8 Hz and 7.2 Hz), 7.55 (br t, 1H, *J* = 1.6 Hz), 7.76 (s, 1H).

**MS (*m/z*):** 291-293 [M + H]^+^, 313-315 [M + Na]^+^.

The 5-(3-chlorophenyl)-3*H*-furo[2,3-*d*]pyrimidin-4-one (**DD297**) has been previously prepared and described by one of us [18].

**REFERENCES**

1. Dauzonne D, Demerseman P (1990) Potassium Fluoride-Promoted Reaction of (2-Chloro-2-nitroethenyl)benzenes with 1,3-Dicarbonyl Compounds - A General-Synthesis of 6,6-Dimethyl-2-nitro-3-phenyl-3,5,6,7-tetrahydro-4(2*H*)benzofuranones and Some Analogs. Journal of Heterocyclic Chemistry 27: 1581-1584.

2. Dauzonne D, Josien H, Demerseman P (1990) (2-Chloro-2-nitroethenyl)benzenes as Synthons - a General-Method for the Preparation of 2,3-Dihydro-2-nitro-3-phenyl-4*H*-furo[3,2-*c*][1]benzopyran-4-ones and 3-Phenyl-4*H*-furo[3,2-*c*][1]benzopyran-4-ones. Tetrahedron 46: 7359-7371.

3. Li JH, Liu WJ (2004) Dabco as an Inexpensive and Highly Efficient Ligand for Palladium-Catalyzed Suzuki−Miyaura Cross-Coupling Reaction. Organic Letters 6: 2809-2811.

4. Li JH, Zhu QM, Xie YX (2006) Pd(OAc)_2_/DABCO-Catalyzed Suzuki–Miyaura Cross-Coupling Reaction in DMF. Tetrahedron 62: 10888-10895.

5. Tian J, Moeller KD (2005) Electrochemically Assisted Heck Reactions. Organic Letters 7: 5381-5383.

6. Li HJ, Wang DP, Xie YX (2005) Pd(OAc)_2_/DABCO as a Highly Active Catalytic System for the Heck Reaction. Synthesis 13: 2193-2197.

7. Li HJ, Wang L (2006) Triethanolamine as an Efficient and Reusable Base, Ligand and Reaction Medium for Phosphane-Free Palladium-Catalyzed Heck Reactions. European Journal of Organic Chemistry 22: 5099-5102.

8. Marchal E, Uriac P, Legouin B, Toupet L, van de Weghe P (2007) Cycloisomerization of γ- and δ-Acetylenic Acids Catalyzed by Gold(I) Chloride. Tetrahedron 63: 9979-9990.

9. Gopalan A, Magnus P (1984) Studies on Terpenes. VIII. Total Synthesis of (±)-Linderalactone, (±)-Isolinderalactone, and (±)-Neolinderalactone, Germacrane Furanosesquiterpenes. Journal of Organic Chemistry 49: 2317-2321.

10. Yamaguchi S, Yamamoto K, Ueda T, Morikawa T, Kawase Y (1989) A New Preparative Method of 4-Hydroxybenzofuran-2-carboxylic Acid Derivatives. Bulletin of the Chemical Society of Japan 62 : 4066-4068.

11. Bhandari K, Shipra S, Girija S, Chandishwar N (2006) Synthesis and Appetite Suppressant Activity of 1-Ayloxy-2-substituted Aminomethyltetrahydronaphthalenes as Conformationally Rigid Analogues of Fluoxetine. Bioorganic and Medicinal Chemistry. 14: 2535-2544.

12. Pinna GA, Sechi M, Paglietti G, Pirisi MA (2003) Addition Reactions of Acetylenic Esters to 6,7-Dihydrobenzo[*b*]furan-4(5*H*)-one, 6,7-Dihydroindol-4(5*H*)-one, 5,6-Dihydrobenzo[*b*]furan-7(6*H*)-one and 5,6-Dihydroindol-7(6*H*)-one Ketoximes. Formation of Reduced Furo[*g*]- and Pyrrol[*g*]-indoles. Journal of Chemical Research 3: 118-120.

13. Shafiee A, Jalilian AR, Rezaei M (2000) Selenium Heterocycles XLIV. Syntheses of 8,9-Dihydro-1,2,3-thiadiazolo[4,5-*a*]-4,7-dihydroxynaphthalene and 1,2,3-Selenadiazolo[4,5-*a*]-4,7-dimethoxynaphthalene. Journal of Heterocyclic Chemistry 37: 1325-1327.

14. Qiu XL, Qing FL (2004) Synthesis of 2′,3′-Dideoxy-2′-difluoromethyl azanucleosides. Synthesis 3: 334-340.

15. Wu MY, Wang MQ, Li K, Feng XW, He T et al. (2011) An Efficient, Transition-Metal-Free Process for the Synthesis of Substituted Dihydrofurans via a Michael/Cyclization Tandem Reaction. Tetrahedron Letters. 52: 679-683.

16. Lampe T, Kast R, Beck H, Stoll F, Becker EM et al. (2010) US Patent. US**2010**/261736 A1.

17. Raimondi W, Dauzonne D, Constantieux T, Bonne D, Rodriguez J (2012) Expeditious, Metal-Free, Domino, Regioselective Synthesis of Highly Substituted 2-Carbonyl- and 2-Phosphorylfurans by Formal [3+2] Cycloaddition. European Journal of Organic Chemistry 31: 6119-6123.

18. Dauzonne D, Adamlaunay A (1992) A Convenient Procedure for the Preparation of 5,6-Dihydro-6-nitro-5-phenylfuro[2,3-*d*]pyrimidin-4(3*H*)-ones and 5-Phenylfuro[2,3-*d*]pyrimidin-4(3*H*)-ones. Tetrahedron 48: 3069-3080.

*** * ***
